# Precision dendritic-supramolecular glycan assemblies for probing multivalent lectin interactions†

**DOI:** 10.1039/d5sc03534a

**Published:** 2025-06-25

**Authors:** Tanvi M. Bhide, Garrett J. Musil, Wade Shipley, Emerson Hall, Alex J. Guseman, Andrea R. Tao, Julia M. Stauber

**Affiliations:** a Department of Chemistry and Biochemistry, University of California, San Diego La Jolla California 92093 USA jstauber@ucsd.edu; b Materials Science and Engineering Program, University of California, San Diego La Jolla California 92093 USA; c Department of Nano and Chemical Engineering, University of California, San Diego California 92093 USA

## Abstract

Multivalent glycan–protein binding events participate in various physiological functions including cell signaling, immune response, and pathogen-host recognition, among others. The complexity of these processes has driven sustained interest in developing densely functionalized glycan nanoassemblies to probe and modulate these important interactions. While synthetic glyconanomaterials have demonstrated significant promise in nanomedicine and biotechnology applications, achieving precise control over their architecture remains challenging. To address this limitation, we present a new method of synthesizing molecular glycan nanoassemblies by integrating the dense functionalization of dendritic architectures with the preorganized scaffolding of metallosupramolecular frameworks. A family of Fe(ii)-anchored superassemblies featuring 24, 36, and 72 peripheral mannosides was prepared and characterized by spectroscopic, microscopic, and light scattering techniques. These assemblies demonstrate strong binding with the lectins Concanavalin A (Con A) and Griffithsin (GRFT), as evaluated by isothermal titration calorimetry (ITC), with the 72-mannose derivative exhibiting low nanomolar binding affinity (*K*_d_ = 28 ± 4 nM for Con A; 12 ± 1 nM for GRFT). The high binding strength of these assemblies highlights the potential of integrating dendritic architectures with rigid metallosupramolecular cores to enhance lectin recognition. Our findings present a new framework for probing glycan protein interactions and offer insights into the design of hybrid glycoassemblies as biomedically-relevant tools.

## Abstract 2

Structurally-defined dendritic Fe(ii)-based glycan nanoassemblies featuring 24, 36, and 72 mannose units enable nanomolar binding with lectin targets.

## Graphical abstract

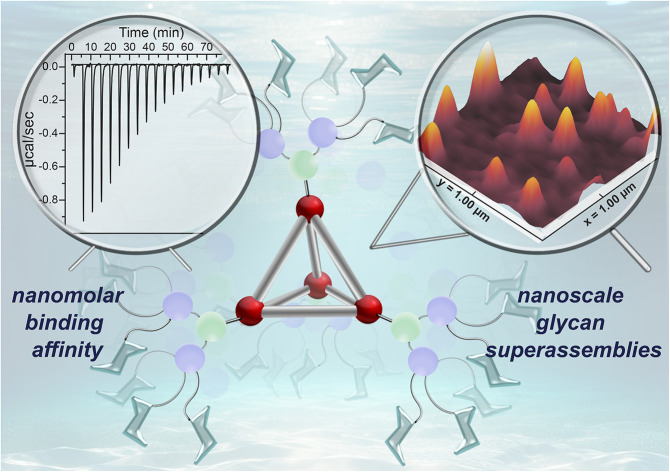

## Introduction

Protein–glycan interactions play key roles in many biological processes including molecular recognition events, cellular communication, and signal transduction, among others.^1–3^ The importance of these biomacromolecular interactions has driven sustained interest across multiple scientific disciplines toward uncovering and understanding the nature of these events, as demonstrated by approaches spanning chemical biology,^4,5^ materials science,^6–8^ and chemical synthesis.^9–11^ Probing these interactions has become increasingly important in biomedical and biotechnology applications,^12–17^ as these processes are central to many diseases and signalling pathways.^18–20^ At the molecular level, protein–glycan recognition occurs through multivalent binding, where multiple weak, non-covalent, and reversible interactions work cooperatively to result in enhanced functional affinity that exceeds the binding strength of the single carbohydrate–protein interaction.^21^ This fundamental principle enables nature to achieve remarkably strong and selective molecular recognition, as evidenced by protein–glycan interactions that typically display micromolar to nanomolar binding affinities (*K*_d_ ∼ 10^−6^ to 10^−9^ M).^12,22^ A detailed understanding of the macromolecular mechanisms underlying carbohydrate recognition is therefore essential for driving the development of innovative tools, treatments, and biomedical agents.

Synthetic platforms that effectively mimic and probe these molecular recognition events hold potential in therapeutic and bioengineering applications, especially in physiological processes where protein–glycan interactions are critical mediators. As the synthesis of glycan assemblies continues to evolve, sustained progress is guided by the need for precise control over molecular architecture and the demand for access to complex structures reminiscent of nature's sophisticated biological ligands.^11,23,24^ A variety of platforms have been used to this end, including dendrimers,^25^ small organic and inorganic molecules,^26–31^ polymers,^15,32,33^ peptides,^34^ nanoparticles,^35^ and templated systems that pre-organize glycans on protein or synthetic scaffolds.^36–38^ However, despite considerable advances in synthetic methodology, examples of structurally well-defined glycoassemblies remain scarce and many approaches lack the generalizable synthetic versatility needed to systematically investigate the relationships governing molecular architecture and binding efficiency.^39–41^ While many conventional platforms like polymers or nanoparticles achieve high affinity, these glycan arrays frequently exhibit intrinsic polydispersity in scaffold size and ligand valency, which can lead to unpredictable binding behaviours and reproducibility challenges.^31,32,42,43^ Monodisperse and structurally tunable ligand assemblies remain underexplored in glycomaterial development, however, despite their importance in the rational design of new multivalent glycomimetics. Motivated by this need, we sought to employ synthetic supramolecular systems that can probe these important recognition events while offering molecular precision and versatility.

**Fig. 1:**
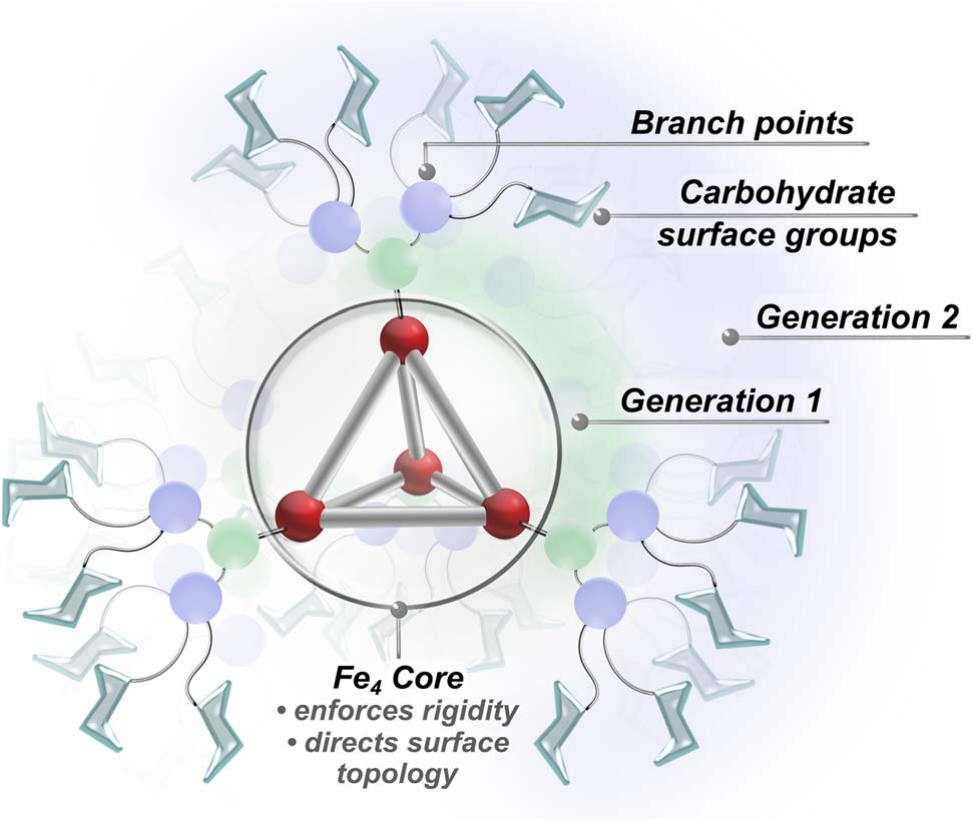
Superassembly framework showing the core structure, dendron generations, and terminal carbohydrate units.

Our approach leverages the complementary advantages of dendritic architectures and metallosupramolecular scaffolding to introduce well-defined hybrid glycan systems with high lectin binding affinity. Recent advances in dendrimer chemistry have provided powerful tools for achieving dense surface functionalization, which allows for the systematic incorporation of saccharide units at specifically defined positions.^34,44–47^ Unlike traditional polymer and nanoparticle platforms, dendrimers offer higher levels of structural precision through their step-wise synthesis. Although dendrimer scaffolds provide valuable architectural control, they often suffer from high conformational flexibility and limited structural preorganization, which is typically needed to maximize lectin binding efficiency.^11^ To address these limitations, we incorporated coordination-driven subcomponent self-assembly in our approach. Coordination-driven subcomponent self-assembly is a powerful synthetic strategy that employs molecular synthons and non-covalent interactions to construct rigid and complex architectures while maintaining the same degree of precision and control attainable in molecular synthesis.^48,49^ While metal-anchored supramolecular architectures provide well-defined frameworks with structured spatial arrangements of surface-grafted elements,^50–53^ these systems typically offer limited peripheral functionalization compared to dendritic platforms. By preserving the advantages of both approaches, this work integrates glycosylated dendritic fragments within rigid metal–organic polyhedral frameworks to introduce a family of densely grafted, architecturally precise superassemblies (Fig. 1) that demonstrate exceptionally strong lectin binding affinity.

## Results and discussion

We recently reported work highlighting the versatility of coordination-driven subcomponent self-assembly^54^ in the synthesis of structurally well-defined saccharide-grafted Fe(ii)-anchored supramolecular assemblies.^55^ This synthetic approach allowed for molecular-level control^48,49^ over assembly size, shape, topology, saccharide surface density, and charge.^56^ Depending on the number of iron centres present in the structure, up to twelve saccharides were introduced on the assembly periphery in a straightforward manner, which enabled precise tuning of the structural valency. Building upon key design principles established from our work, the present study employs diethyleneglycol (DEG) linkages to tether saccharide units to supramolecular frameworks, as these linkers offered optimal carbohydrate spatial arrangements and flexibility that enhanced lectin binding affinity.^56^

**Fig. 2:**
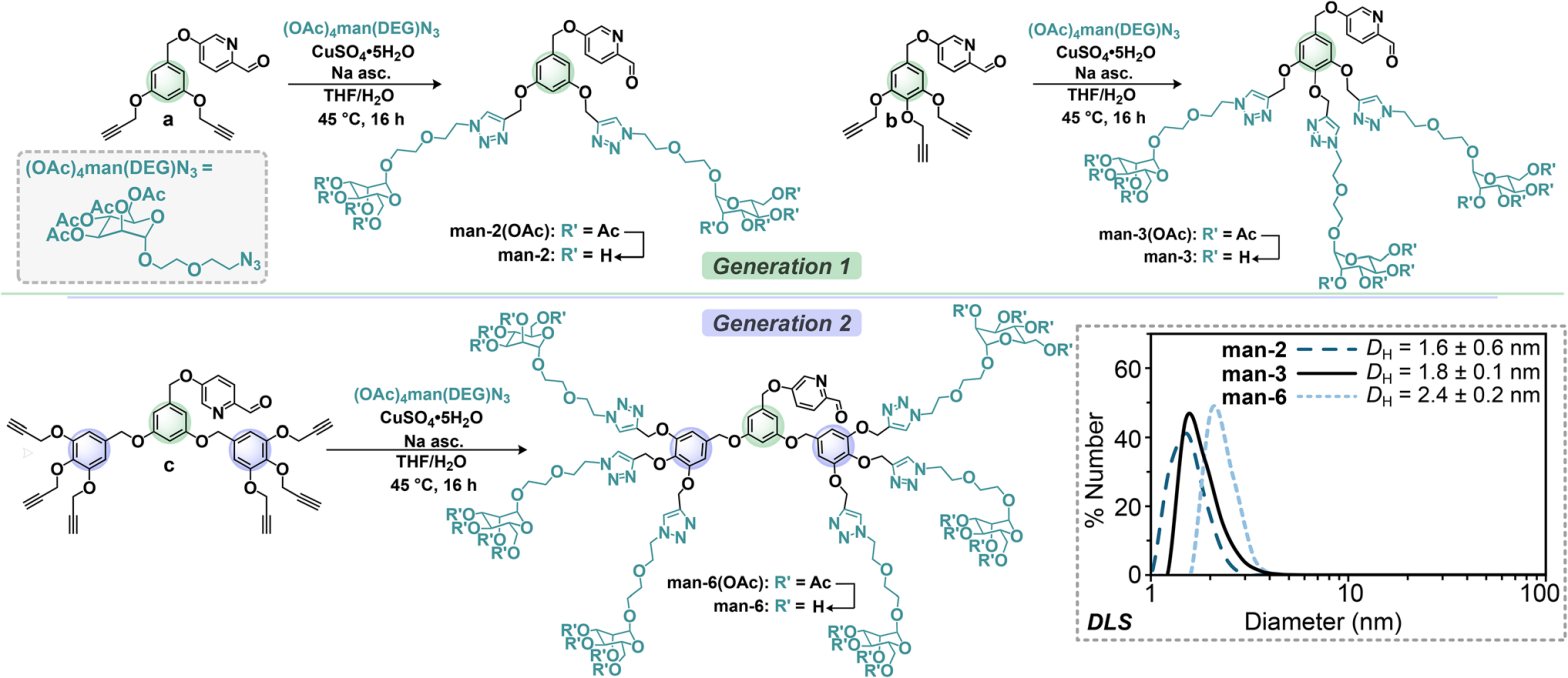
Synthetic schemes for the preparation of dendritic subcomponents man-2, man-3, and man-6 from a, b, and c precursors, respectively, with corresponding DLS data (bottom right, measured in H_2_O, 1 mM).

**Scheme 1:**
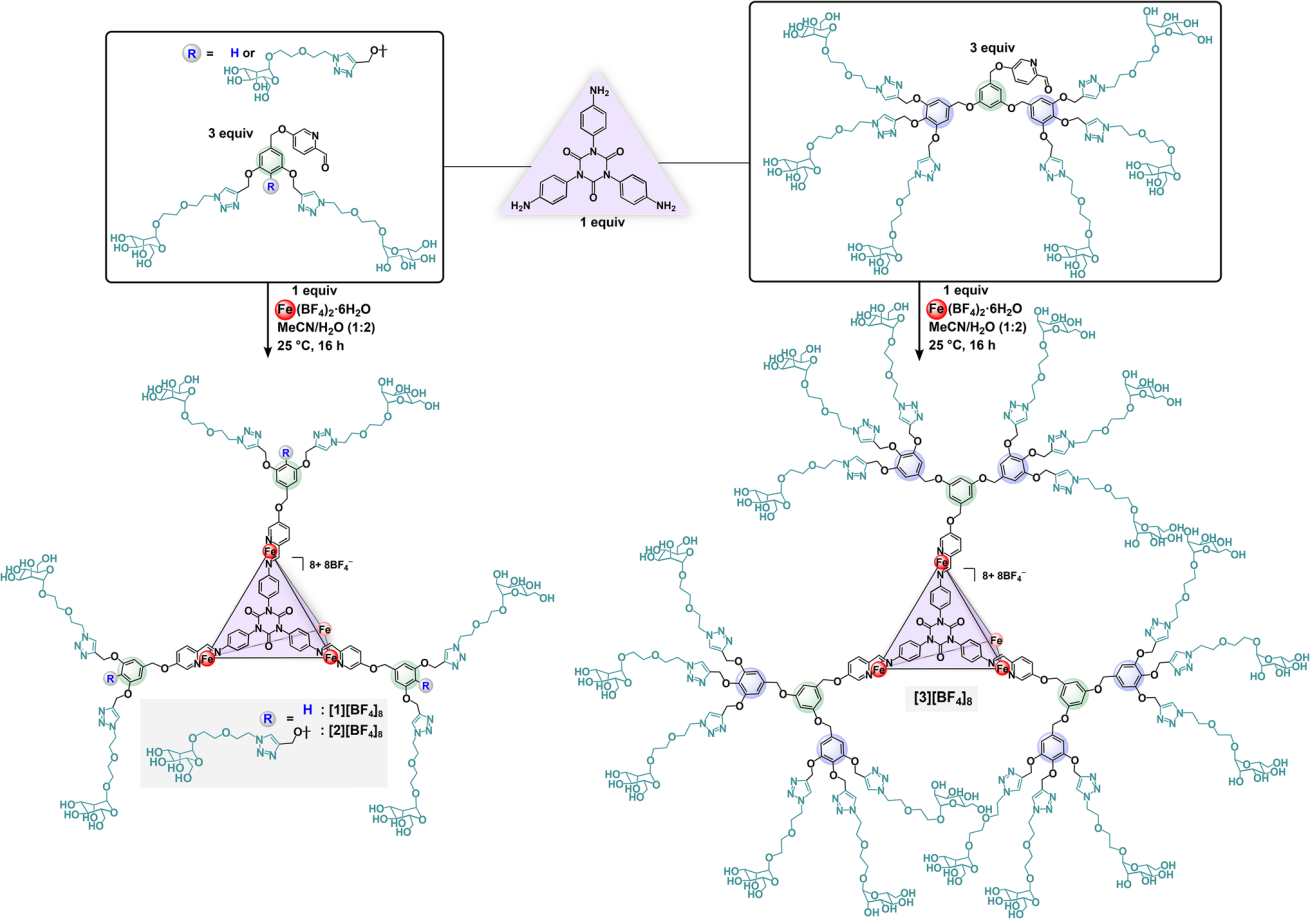
Fe(ii)-directed subcomponent self-assembly reactions to prepare [1][BF_4_]_8_, [2][BF_4_]_8_, and [3][BF_4_]_8_. Only one of four face-capping ligands is shown for clarity.

Here, we have expanded the synthetic landscape by introducing nanoassemblies with significantly higher valencies than previously reported. This report presents a new series of dendritic glycan picolinaldehyde ligands bearing two, three, and six mannose residues that were prepared through copper-catalysed azide–alkyne cycloaddition chemistry (CuAAC, Fig. 2). When paired with a tritopic amine subcomponent that enforces a tetrahedral, tetrametallic core, these combinations gave rise to imino-pyridine supported glycan superassemblies bearing 24, 36, and 72 mannosides through an iron(ii)-templated synthesis (Scheme 1). Evaluation of these hybrid glycan assemblies revealed strong binding affinity toward the lectins Concanavalin A (Con A) and Griffithsin (GRFT), with dissociation constants reaching low nanomolar levels. Notably, binding to Con A surpassed that of our previously reported systems by over two orders of magnitude.^56^

### Dendron ligand synthesis

The first generation of di- and tri-substituted glycodendrimer picolinaldehyde ligands was synthesized starting from commercially available 3,5-dihydroxymethylbenzoate and methyl gallate reagents, respectively. These precursors were treated with propargyl bromide, followed by reduction with LiAlH_4_. Bromination of the benzyl alcohol products with PBr_3_ yielded the corresponding benzyl bromide analogues, which were treated with 5-hydroxypicolinaldehyde to afford the “clickable” terminal alkyne ligand precursors (a, b, Fig. 2). These products were subsequently treated with an excess of α-d-mannopyranosyl(diethyleneglycol) azide under CuAAC conditions with CuSO_4_·5H_2_O as the catalyst and sodium ascorbate as the reducing agent. Flash liquid chromatography purification provided the acetate-protected di- (man-2(OAc)) and tri-mannoside (man-3(OAc)) picolinaldehyde derivatives in 73 and 79 percent yields, respectively. Characterization of these products by ^1^H NMR spectroscopy demonstrated complete conversion of the terminal alkyne units of a and b into triazole moieties as evidenced by the disappearance of the alkyne resonances (*δ* 2.52 (a); 2.49, 2.46 (b) ppm in CDCl_3_), and the appearance of triazolyl resonances located at *δ* 7.77 and 7.84 ppm for man-2(OAc) and man-3(OAc), respectively. In the ^13^C NMR spectrum, the signals located at *δ* 144.4 and 124.0 ppm for man-2(OAc), and *δ* 144.4, 143.5, 124.6, and 124.3 ppm for man-3(OAc) additionally confirmed the presence of triazolyl carbons.^9,57^ The ^1^H and ^13^C NMR spectra reveal single species in solution that exhibit two-fold rotational symmetry, consistent with the proposed molecular structures.

The Fourier-transform infrared (FTIR) spectra of both subcomponents lack the characteristic signatures of alkyne or azide functional groups, which supports covalent attachment of the saccharide units to the picolinaldehyde anchor (ESI Section S2.1†), and clean isolation of the 1,2,3-triazole glycosides. Quantitative de-*O*-acetylation under standard Zemplén conditions (cat. NaOMe, MeOH, Fig. 2)^58^ yielded the deprotected α-mannopyranoside derivatives, man-2 and man-3, after neutralization with the acidic Amberlyst-15® ion-exchange resin. Full deprotection of the mannopyranose groups was confirmed by ^1^H NMR spectroscopy based on the disappearance of acetate resonances located between *δ* 1.8–2.1 ppm (D_2_O). Electrospray ionization mass spectrometry (ESI-MS(+)) analyses of the products in positive mode displayed signals corresponding to the protonated molecular ions [M + *n*H]^*n*+^, which confirmed both the empirical formula and integrity of the species. Dynamic light scattering (DLS) measurements provided further characterization and offered insights into the molecular dimensions of these compounds by determining their hydrodynamic particle size distributions in solution. Measurements conducted in aqueous media (1 mM) demonstrated well-defined size distributions for both man-2 and man-3, with hydrodynamic diameters (*D*_h_) of 1.6 ± 0.6 and 1.8 ± 0.1 nm respectively (Fig. 2). These dimensions align with the expected values for non-aggregated single particles, providing strong evidence for the structural integrity and monodispersity of these species in solution.

We next focused on expanding this platform by preparing a second-generation dendron ligand based on a branched hexaalkynylated picolinaldehyde core that would provide access to a hexa-mannoside subcomponent. The synthesis of hexa-functionalized dendritic architecture, man-6, mirrored the procedure described for the syntheses of man-2 and man-3 following the initial coupling of 3,4,5-trispropargyloxybenzyl bromide (two equiv.) with 3,5-dihydroxymethylbenzoate in the presence of K_2_CO_3_ and 18-crown-6.^59^ Reduction of the methyl ester with LiAlH_4_ followed by bromination provided the hexapropargyloxy benzylbromide dendrimer core, which enabled installation of the picolinaldehyde unit *via* S_N_2 substitution chemistry. With this building block, the targeted sixfold-substituted glycan subcomponent, man-6(OAc), was synthesized through a hexa-click reaction following the same CuAAC parameters outlined for the first-generation derivatives (*vide supra*). Characterization of man-6(OAc) by ^1^H and ^13^C NMR spectroscopy revealed signatures consistent with those observed for the man-2(OAc) and man-3(OAc) analogues, supporting successful synthesis and isolation of the desired two-fold symmetric glycoconjugate. FTIR spectroscopy was employed to confirm the absence of alkyne and azide functional groups, which further substantiated the identity of the desired compound. ESI-MS(+) analysis of the deprotected man-6 glycan was consistent with the protonated [M + 3H]^3+^ ion, thereby confirming the identity and molecular composition of the ligand. The ^1^H NMR spectrum of deprotected man-6 displayed broadened signals when recorded at ambient temperature at concentrations suitable for spectroscopic analysis (10–30 mM), which is consistent with the tendency of densely functionalized glycan assemblies to form clustered aggregates in aqueous solution.^9^ Collection of ^1^H NMR data in CD_3_ OD, however, led to sharper signals and reduced aggregation, likely due to the disruption of carbohydrate–aromatic π interactions that occur in aqueous solution.^60^ At significantly lower concentrations (1 mM) in water, however, a hydrodynamic diameter aligning with a single particle measuring *D*_h_ = 2.4 ± 0.2 nm was observed (Fig. 2), further demonstrating the architectural integrity of the product.

### Superassembly synthesis

With man-2, man-3, and man-6 subcomponents in hand, we focused on preparing saccharide grafted supramolecular assemblies utilizing our established Fe(ii)-driven subcomponent self-assembly methodology.^55,56^ The glycan ligands were paired with the isocyanurate-based tritopic amine subcomponent shown in Scheme 1. This subcomponent was selected due to its water-solubilizing polar groups that impart the aqueous compatibility required for this work when paired with a water-soluble iron(ii) salt. Furthermore, we demonstrated^56^ that this tritopic amine subcomponent directs the formation of a face-capped Fe_4_L_4_ structural motif,^61–63^ where four Fe(ii) centres occupy the vertices with pseudo-octahedral geometry, while trigonally-symmetric iminopyridine ligands span the faces.^64,65^ Since each iron centre coordinates three bidentate ligands based on single-crystal X-ray diffraction studies of structurally analogous M_4_L_4_ (M = Fe(ii), Co(ii), Zn(ii)) assemblies,^50,64–67^ the resulting complexes supported by man-2, man-3, and man-6 give rise to glycan superassemblies grafted with 24, 36, and 72 saccharides, respectively. This structural configuration provides access to architectures decorated with significantly higher saccharide loadings that are two-, three-, and six-fold greater than those of our previously reported systems.^55,56^ This enhanced functionalization offers potential for both stronger lectin binding affinity and broader applications for these constructs.

**Fig. 3:**
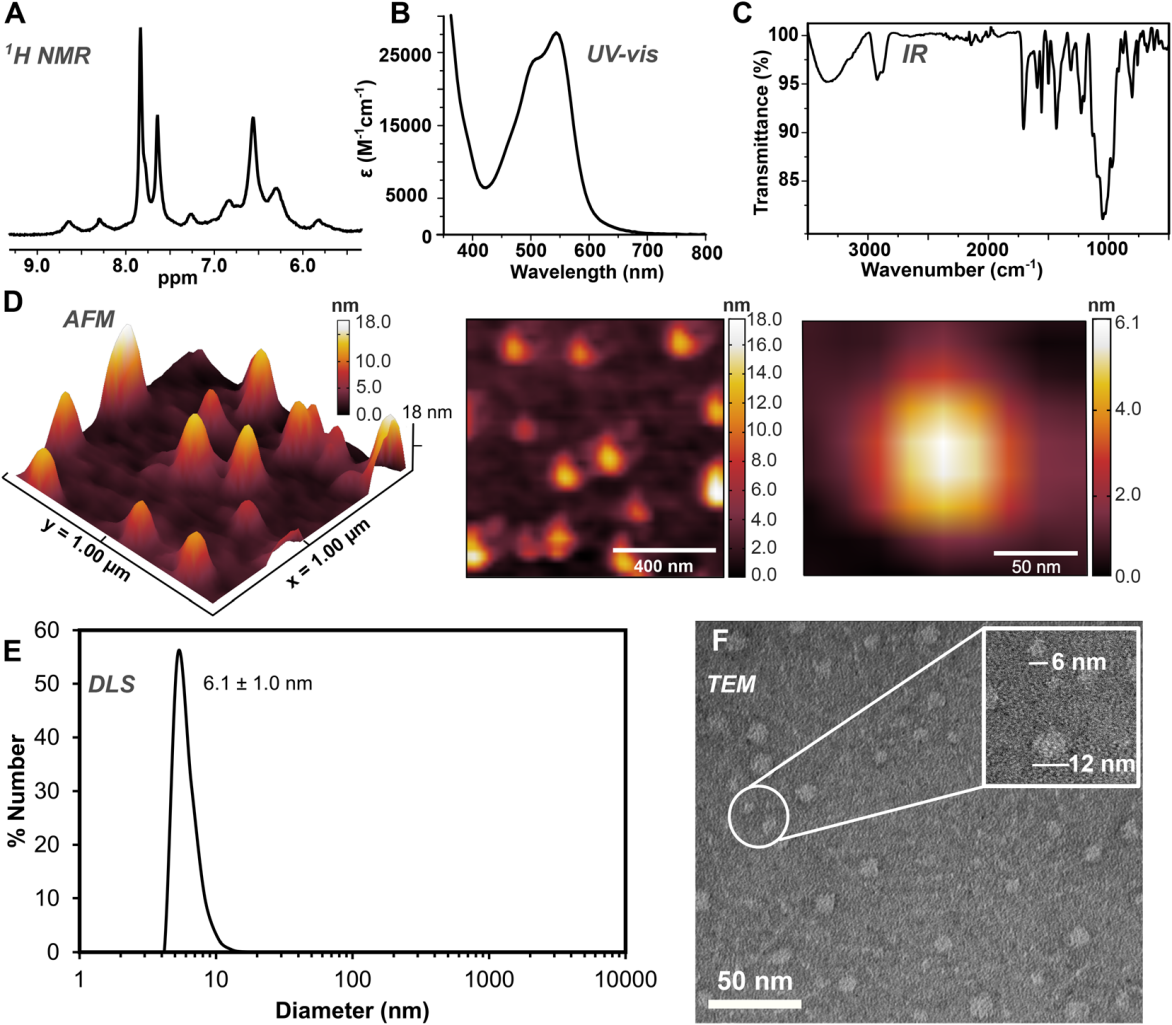
Characterization data of [3][BF_4_]_8_: (A) ^1^H NMR spectrum (D_2_O, 45 °C); (B) UV-vis spectrum (H_2_O, 50 μM); (C) ATR-IR spectrum; (D) AFM images and height profiles (tapping mode, air, 298 K) of a drop-cast H_2_O solution (0.2 μM) on mica; (E) DLS measurement (50 μM, 85% v/v DMSO/H_2_O); (F) negative-contrast electron micrograph (uranyl acetate).

Accordingly, treatment of triamine isocyanurate (1 equiv.) with subcomponents man-2, man-3, and man-6 (3 equiv.) in the presence of Fe(BF_4_)_2_·6H_2_O (1 equiv.) in H_2_O/MeCN (2 : 1) solvent mixtures at 25 °C afforded the 24- ([1][BF_4_]_8_), 36- ([2][BF_4_]_8_), and 72-fold ([3][BF_4_]_8_) mannose-substituted assemblies, respectively (Scheme 1), which were subsequently purified *via* centrifugal ultrafiltration. To provide a thorough analysis of their structural and molecular properties, the three superassemblies were characterized by spectroscopic, microscopic and light scattering techniques. The ^1^H NMR spectra of [1]^8+^, [2]^8+^, and [3]^8+^ collected in D_2_O at 25 °C revealed broadened resonances with increased linewidths, which is attributed to slow dynamic motion of the cages in solution, and is typical of large molecular-weight molecules (MW [1][BF_4_]_8_ = 13 200, [2][BF_4_]_8_ = 17 400, [3]^8+^ = 32 400 g mol^−1^) that display short *T*_2_ relaxation times.^26,68–70^ We therefore collected ^1^H NMR spectra of these systems at slightly elevated temperatures to increase the overall molecular tumbling rate, which resulted in moderately sharpened and more well-defined signals for all three complexes (ESI Fig. S44, S47 and S50†). However, due to the limited thermal stability of these systems, we were unable to collect ^1^H NMR data at temperatures exceeding 45 °C. Despite these limitations, the ^1^H NMR spectra of [1]^8+^, [2]^8+^, and [3]^8+^ display signals that are consistent with single, *T*-symmetric structures in solution, which enable confirmation of the structural integrity of the species. Imine-bond formation during the self-assembly reactions was confirmed by the disappearance of aldehyde resonances located at *δ* 9.55, 9.79, and 9.97 ppm (D_2_O), concomitant with the appearance of new, upfield-shifted signals attributed to the imine protons, located at *δ* 9.15, 8.85, and 8.77 ppm for complexes [1][BF_4_]_8_, [2][BF_4_]_8_, and [3][BF_4_]_8_, respectively. When measured at 45 °C, the ^1^H NMR spectra of [2][BF_4_]_8_ and [3][BF_4_]_8_ (Fig. 3A) each displayed two distinct singlets (*δ* 7.99, 7.74 ppm for [2][BF_4_]_8_; *δ* 7.96, 7.77 ppm for [3][BF_4_]_8_), corresponding to the two chemically unique 1,2,3-triazole methine protons present in each complex. In contrast, the ^1^H NMR spectrum of [1][BF_4_]_8_ contains a single resonance located at *δ* 8.21 ppm, which is consistent with one unique triazolyl methine proton.

Additional spectroscopic characterization was performed by UV-vis analysis (50 μM, H_2_O) for all superassemblies, which displayed characteristic metal-to-ligand charge transfer excitations for low-spin iron(ii) in an iminopyridine coordination environment (*λ*_max_ = 505, 550 nm, Fig. 3B), which align well with the data reported for our first series of Fe(ii) glycan assemblies.^55,56^ The FTIR spectra of complexes [1][BF_4_]_8_, [2][BF_4_]_8_, and [3][BF_4_]_8_ provide further evidence supporting the formation of an imine bond during self-assembly reactions, as indicated by the absence of aldehyde bands and the presence of strong vibrations ascribed to the imino C=N stretching mode ([3][BF_4_]_8_*ν*_C=N_ 1558 cm^−1^, Fig. 3C).^71^

To characterize the solution-phase behaviour of the assemblies, DLS measurements were employed to determine their hydrodynamic diameters. Regardless of the concentrations used in water, we observed size distributions for all three systems that exceed the size of a single particle (5 μM: [1]^8+^: 77.1 ± 8.7 nm, [2]^8+^: 60.5 ± 6.5 nm, [3]^8+^: 42.4 ± 3.6 nm, ESI Section S3†). These findings are representative of weak aggregation in water and are consistent with behaviour observed for other densely functionalized glycomolecules in aqueous solution.^9,72^ Alternatively, in mixed DMSO/H_2_O (85% v/v) solutions, single size distributions were observed for [1]^8+^, [2]^8+^, and [3]^8+^, measuring 3.8 ± 0.8, 4.1 ± 0.6, and 6.1 ± 1.0 nm (Fig. 3E), respectively. These data align with the presence of non-aggregated single particles in solution, and reflect similar aggregation behaviour observed for the closely-related mannosylated fullerenes reported by Martín *et al.*^9^ To provide visualization of the nanoassemblies and to offer further support for the size of the single particles observed by DLS, we conducted transmission electron microscopy (TEM) measurements. Due to the limited stability of these superassemblies in DMSO (*ca.* 15 min at 5 μM), sample preparation for TEM analysis required the use of aqueous solutions. The TEM images reveal the presence of small spherical clusters consisting of aggregates of two to three individual molecules (*ca.* 9–20 nm), and discrete, spherically shaped non-aggregated single particles. Analysis of the electron micrographs demonstrates individual non-aggregated particles with diameters of approximately 5, 5, and 6 nm for [1]^8+^, [2]^8+^, and [3]^8+^, respectively, as displayed by the representative data for [3]^8+^ shown in Fig. 3F. These dimensions are in excellent agreement with measurements obtained from DLS measurements and independently confirm the nanoassembly sizes. Atomic force microscopy (AFM) was used to further validate the molecular sizes of the three systems. Fig. 3D provides a representative AFM image of complex [3]^8+^ that reveals distinct populations of individual particles with diameters of *ca.* 6 nm, in addition to collections of clustered aggregates measuring *ca.* 18 nm. These findings provide support for the particle dimensions determined by TEM and DLS analyses, establishing consistent nanoscale size distributions across multiple analytical techniques.

Unfortunately, the molecular ions of these products could not be detected *via* ESI-MS(+) or MALDI-TOF-MS analyses. Reports indicate similar difficulties detecting molecular ion peaks for glycoclusters of comparable size, as transferring high molecular weight glycoassemblies into the gas phase during matrix-assisted laser desorption/ionization-time-of-flight mass spectrometry can prove very difficult due to high degrees of fragmentation and the formation of matrix adducts.^9,73,74^ Additionally, MALDI-TOF-MS analyses in this study were further complicated by the high 8+ charges of the systems.

After confirming structural integrity and nanoscale dimensions by multiple analytical techniques, we evaluated the stability of this platform under biologically relevant conditions. UV-vis spectra of [3]^8+^ recorded across a pH range of 5–10, in PBS and Tris buffers, and in cell media at 37 °C did not display significant changes over a 24 h period, with no shift in *λ*_max_ or significant loss of intensity (ESI Section S6†). These results indicate that [3]^8+^ retains its structural integrity and coordination environment under physiological conditions and supports the suitability of this platform for biological applications.

### Evaluation of superassembly lectin recognition

We next investigated the protein recognition capabilities of each assembly with Con A. Con A is a well-studied α-mannose-binding lectin derived from jack bean (*Canavalia ensiformis*)^75^ seeds that exists as a homotetramer at physiological pH and a homodimer at pH < 6.^76^ Due to its structural similarity to many bacterial and animal lectins, Con A serves as a model system for investigating protein–carbohydrate interactions that govern important processes including cell adhesion, cell signalling, and immune response.^77–79^

The binding capabilities of superassemblies [1]^8+^, [2]^8+^, and [3]^8+^ with Con A were evaluated by isothermal titration calorimetry (ITC). It is well-known that the precipitation of cross-linked aggregates during ITC experiments has a significant negative effect on the reliability of thermodynamic data.^80,81^ Therefore, in order to minimize the potential of precipitation during measurements, all experiments in this study were performed at low protein concentration, low salt concentrations (NaCl, CaCl_2_, MnCl_2_), and in acidic conditions (pH 4.8) with Con A in its predominantly dimeric form. As the separation between individual monomeric binding sites on Con A (*ca.* 8 nm)^82^ prevents chelation of the dimeric lectin by the glycoassemblies (4–6 nm), isotherm data were fitted using a single-site binding model based on monomeric Con A. The dissociation constants (*K*_d(avg)_) for the interactions of [1]^8+^ and [2]^8+^ with Con A were determined to be 1.1 ± 0.1 μM and 290 ± 4 nM (Table 1), respectively, through three independent titrations of each complex. The *K*_d_ values illustrate significant affinity enhancements, with 17- and 67-fold increases compared to the binding of our previously reported twelve-mannose analogue with Con A ([Fe_4_L_4_-12][BF_4_]_8_; *K*_d_ = 18.9 ± 2.5 μM).^56^ We attribute the increase in binding affinity to the enhanced cluster glycoside effect^83–85^ resulting from the significantly higher density of peripheral saccharides.

**Table 1:** Dissociation constants (*K*_d_) determined for the interaction between [Fe_4_L_4_-12][BF_4_]_8_,^56^ [1][BF_4_]_8_, [2][BF_4_]_8_, and [3][BF_4_]_8_ with Con A, and GRFT, and respective valencies (*n*) of each assembly. Per-mannose normalized *K*_d_ values (*K*_d_/valency) are shown in the row below each entry to illustrate the mannose binding enhancement gained through the multivalent effect

Assembly	*K*_d_ [Con A]	*K*_d_ [GRFT]	Valency (*n*)
[Fe_4_L_4_-12][BF_4_]_8_	18.9 ± 2.5 μM (ref. 56)	251 ± 19 nM	12
	1.58 ± 0.21 μM per man	21 ± 2 nM per man	
[1][BF_4_]_8_	1.1 ± 0.1 μM	160 ± 21 nM	24
	46 ± 4 nM per man	7 ± 1 nM per man	
[2][BF_4_]_8_	290 ± 4 nM	63 ± 4 nM	36
	8.1 ± 0.1 nM^a^ per man	1.8 ± 0.1 nM^a^ per man	
[3][BF_4_]_8_	28 ± 4 nM	12 ± 1 nM	72
	390 ± 60 pM^a^ per man	170 ± 10 pM^a^ per man	

a For per-mannose *K*_d_ values below 1 nM, additional decimal places are reported to avoid rounding artifacts that obscure sub-nanomolar values.

**Fig. 4:**
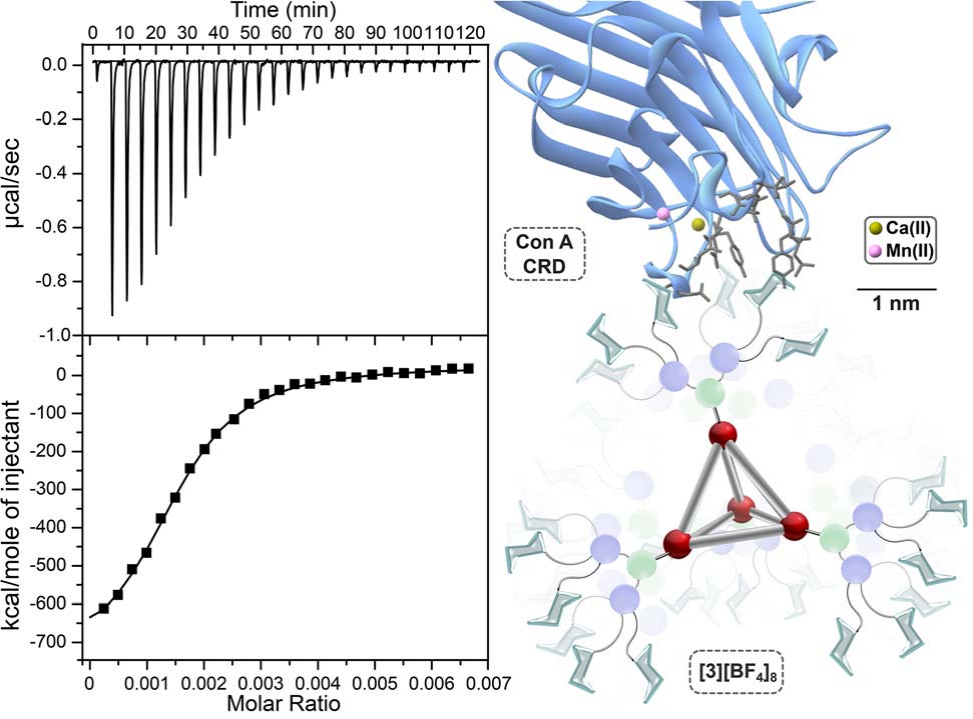
Left: ITC thermogram (top) and fitted binding isotherm (bottom) for the binding of [3][BF_4_]_8_ to dimeric Con A. Right: Model of the binding interaction showing the Con A CRD (PDB code 1QDC).

Complex [3]^8+^ demonstrated the strongest binding to Con A among the superassembly family, with a calculated dissociation constant of 28 ± 4 nM (Fig. 4). This binding strength surpasses α-d-mannose (470 μM),^86,87^ by approximately four orders of magnitude, representing the strongest interaction observed for any supramolecular glycoassembly in our current library of 17-mannosylated complexes.^55,56^ The binding affinity demonstrated by [3]^8+^ approaches levels typically observed for densely-functionalized nanoparticle and polymer-based platforms with Con A, with only a few examples reaching such interaction strengths to our knowledge.^86–89^ Furthermore, when normalized on a per-saccharide basis, the immobilization of mannose units on the superassembly framework increases the binding strength of each mannoside by a factor of 231. This outcome is particularly noteworthy, as [3]^8+^ exhibits a greater binding enhancement on a per-sugar basis than many existing polymeric, dendritic, and nanoparticle systems, which typically require significantly higher carbohydrate densities to achieve comparable affinity,^86,87,90^ which highlights the structural efficiency of this superassembly design.

Since the separation between dimeric Con A binding sites precludes chelation by the glycoassemblies, the enhanced affinity of these systems is more likely attributed to statistical rebinding and crosslinking multivalent mechanisms. These operating mechanisms are well-precedented for other multivalent platforms,^91^ and can substantially enhance apparent affinity without requiring simultaneous engagement of multiple sites on a single lectin. This interpretation aligns with our prior work on the lower-valent, Fe_4_L_4_ dodeca-functionalized analogues,^56^ where extensive DLS experiments provided evidence consistent with crosslinking and statistical rebinding.

We further attribute the enhanced binding capabilities of these systems to the preorganized supramolecular framework, which combines the structural rigidity of the inorganic cage core with the flexibility of the dendritic glycan ligand, enabling these systems to achieve a similar binding strength to systems with much higher valencies. Although the Fe_4_ core is positioned away from the binding site (*ca.* 2 nm), its structural rigidity pre-organizes the terminal mannoside groups, likely reducing the configurational entropic penalty typically observed in flexible multivalent systems such as conventional glycodendrimers. It is well-documented that preorganized glycan architectures can enhance lectin binding while minimizing overall entropy cost.^92,93^ The combination of the rigid Fe_4_L_4_ core with the flexible dendron ligand fragments has the potential to simultaneously provide flexibility for the systems to access and adapt to the lectin CRD (carbohydrate recognition domain) while reasonably minimizing the conformational enthalpy penalty. Striking the right balance between rigidity and flexibility in multivalent glycosystems has become a central focus within the expanding field of glyconanotechnology, as architectural optimization has been shown to increase avidity by several orders of magnitude beyond what is possible through statistical rebinding events alone.^11,94,95^

Building on these findings, we expanded this work to investigate the interactions between our superassembly platform and GRFT, a broad-spectrum antiviral lectin that specifically recognizes high-mannose glycans.^96^ Consistent with the binding trend observed for the assemblies with Con A, glycocluster association with monomeric GRFT also displayed clear valency-dependent binding, with complex [3]^8+^ exhibiting the highest affinity (*K*_d_ = 12 ± 1 nM). This binding strength represents a significant enhancement over that of monovalent α-d-mannose, which binds GRFT with a *K*_d_ value of approximately 100 μM,^97^ representing a nearly 8000-fold increase in overall binding strength, and a 110-fold enhancement per saccharide. These results demonstrate that the multivalent presentation of mannosides on the dendritic metallosupramolecular framework not only enhances affinity toward canonical targets like Con A but also extends to therapeutically relevant lectins such as GRFT. The ability of these systems to engage both lectins with nanomolar affinity highlights the potential utility of this approach as a broadly applicable glycan display platform for probing and modulating diverse carbohydrate–protein interactions.

We acknowledge that DLS, TEM, NMR, and AFM analyses collectively reveal some degree of aggregation of the supramolecular constructs in water, which likely stems from weak intermolecular carbohydrate–aromatic π-interactions rather than structural decomposition of the underlying assembly framework. This interpretation is supported by retained imine proton resonances in the ^1^H NMR spectra in aqueous solution (*δ* 9.15–8.77 ppm) and unchanged UV-vis profiles (*λ*_max_ 505–550 nm) under various conditions, confirming the integrity of the metal–ligand bonds within the complexes. While these assemblies are architecturally well-defined and monodisperse under mixed solvent conditions, aggregation in aqueous environments may affect the apparent glycan valency during lectin binding and should be taken into consideration when interpreting affinity measurements. Since aggregation may lead to an underestimation of the effective valency, the *K*_d_ values must be interpreted with some caution, as the structural valency may not be entirely representative of monomeric binding. These assemblies, however, remain fully soluble in water, suggesting they remain dynamic and accessible for molecular interactions. Similar behaviour has been observed in other densely functionalized glycan architectures, where self-aggregation does not preclude their use in biomedical applications but rather represents an intrinsic structural feature that can influence binding interactions.^9,72^ Nevertheless, these supramolecular architectures represent unique and well-characterized examples of discrete glycoassemblies capable of high-affinity interactions, offering a versatile platform for further development in biomedical and therapeutic contexts.^9,72^

## Conclusions

The molecular recognition of carbohydrates in biological systems is directly tied to both the chemical composition and spatial presentation of surface glycans. Motivated by the need to understand and probe these complex interactions, we designed a new class of glycan superassemblies by introducing a synthetic strategy that merges dendritic architectures with coordination-driven self-assembly. This approach enabled the isolation of molecular glycoassemblies exhibiting exceptional lectin binding capabilities. Picolinaldehyde di-, tri-, and hexa-substituted mannoside subcomponents were synthesized employing CuAAC click–chemistry reactions and used to prepare molecular assemblies bearing 24, 36, and 72 saccharides. The three superassemblies were characterized by standard spectroscopic techniques (^1^H NMR, FTIR, UV-vis), as well as DLS, TEM and AFM measurements. All three assemblies were evaluated as multivalent binders to Con A and GRFT, revealing *K*_d_ values in the low micromolar to nanomolar range. Notably, the 72-mannoside derivative demonstrated exceptionally strong binding, which likely stems from intentional architectural design that balances scaffold rigidity with localized flexibility, establishing important principles for developing increasingly sophisticated molecular systems capable of engaging in biomolecular recognition events. The versatile nature of this synthetic strategy allows for the systematic exploration and expansion of diverse scaffold architectures and functionalities as we aim to enhance our fundamental understanding of multivalent glycan recognition and provide synthetic approaches that leverage supramolecular chemistry as a biomedically-relevant tool.^98,99^

## Author contributions

J. M. S. designed the project and experiments. J. M. S. and T. M. B. carried out all synthetic work and the characterization of compounds. J. M. S. performed ITC binding studies, and G. J. M. performed TEM and DLS characterization. W. S. and G. J. M. performed AFM experiments, which were supervised by A. R. T. The GRFT protein was expressed by E. H. under the supervision of A. J. G. The manuscript was written by J. M. S., and all authors gave final approval.

## Conflicts of interest

There are no conflicts to declare.

## Supplementary Material

SC-016-D5SC03534A-s001

## Data Availability

The data that support the findings of this study are available in the ESI† of this article.
