# Supplementary material for: Precision dendritic-supramolecular glycan assemblies for probing multivalent lectin interactions
Source: Chem Sci. 2025 Jun 25;16(30):13636–45. doi: 10.1039/d5sc03534a (PMC12236194; doi:10.1039/d5sc03534a)
Supplement: SC-016-D5SC03534A-s001 [file SC-016-D5SC03534A-s001.pdf]

Supplementary Information (SI) for Chemical Science.
This journal is © The Royal Society of Chemistry 2025

Supporting information for the paper entitled,

Precision Dendritic-Supramolecular Glycan Assemblies for Probing Multivalent Lectin Interactions

Tanvi M. Bhide,[1] Garrett J. Musil,[1] Wade Shipley,[2] Emerson Hall,[1] Alex J. Guseman,[1] Andrea R. Tao,[1,2,3] and Julia M. Stauber*[1]

Affiliations:

[1]*Department of Chemistry and Biochemistry, University of California, San Diego, La Jolla, California, 92093, United States*

[2]*Materials Science and Engineering Program, University of California, San Diego, La Jolla, California, 92093, United States*

[3]*Department of Nano and Chemical Engineering, University of California, San Diego, California, 92093, United States*

*Correspondence to: jstauber@ucsd.edu

S1. General considerations 4
S1.1. Materials 4
S1.2. Methods 4
S2. Synthetic procedures and characterization data for all compounds 5
S2.1. Organic subcomponents 5
S2.1.1. 2-(2-bromoethoxy)ethanol 5
S2.1.2. 2-(2-azidoethoxy)ethyl$(OAc)_4$-α-D-mannose 6
S2.1.3. 3,5-dihydroxybenzyl alcohol 8
S2.1.4. 3,5-bis(propynyloxy)benzoate 9
S2.1.5. 5-(hydroxymethyl)-1,3-bis(propynyloxy)benzene 10
S2.1.6. 5-(bromomethyl)-1,3-bis(propynyloxy)benzene 11
S2.1.7. 5-((3,5-bis(propynyloxy)benzyl)oxy)picolinaldehyde (a) 12
S2.1.8. Man-2(OAc) 14
S2.1.9. Man-2 17
S2.1.10. Methyl 1,2,3-tris(propynyloxy)benzoate 19
S2.1.11. 5-(hydroxymethyl)-1,2,3-tris(propynyloxy)benzene 20
S2.1.12. 5-(bromomethyl)-1,2,3-tris(propynyloxy)benzene 21
S2.1.13. 5-((1,2,3-tris(propynyloxy)benzyl)oxy)picolinaldehyde (b) 22
S2.1.14. Man-3(OAc) 25
S2.1.15. Man-3 28
S2.1.16. (3,5-bis((3,4,5-tris(propynyloxy)benzyl)oxy)phenyl)methanol 30
S2.1.17. (5-(bromomethyl)-1,3-phenylene)bis(1,2,3-tris(propynyloxy)benzene) 32
S2.1.18. 5-(1,3-bis(3,4,5-tris(propynyloxy)))picolinaldehyde (c) 34
S2.1.19. Man-6(OAc) 37
S2.1.20. Man-6 40
S2.2. Fe(II) mannosylated self-assembly complexes 43
S2.2.1. [**1**]$[BF_4]_8$ 44
S2.2.2. [**2**]$[BF_4]_8$ 47
S2.2.3. [**3**]$[BF_4]_8$ 50
S3. Dynamic Light Scattering (DLS) 53
S3.1. Man-2 53
S3.1.1. Measurement in H_2O (1 mM) 53
S3.2. Man-3 53
S3.2.1. Measurement in H_2O (1 mM) 53
S3.3. Man-6 54

S3.3.1. Measurement in H_2O (1 mM)....54
S3.4. [**1**][BF_4]$_8$....54
S3.4.1. Measurement in H_2O (5 μM)....54
S3.4.2. Measurement in DMSO/H_2O (85% v/v, 50 μM)....54
S3.5. [**2**][BF_4]$_8$....55
S3.5.1. Measurement in H_2O (5 μM)....55
S3.5.2. Measurement in DMSO/H_2O (85% v/v, 50 μM)....55
S3.6. [**3**][BF_4]$_8$....56
S3.6.1. Measurement in H_2O (5 μM)....56
S3.6.2. Measurement in DMSO/H_2O (85% v/v, 50 μM)....56
S4. Transmission Electron Microscopy (TEM)....57
S5. Atomic Force Microscopy (AFM)....58
S5.1. [**1**][BF_4]$_8$....58
S5.2. [**2**][BF_4]$_8$....58
S5.3. [**3**][BF_4]$_8$....58
S6. Stability Studies of [3][BF_4]$_8$ to various buffers, and pH values.....59
S6.2. DMEM cell medium, 10% fetal bovine serum at 37 °C....59
S6.3. Carbonate buffer, pH 10....59
S6.4. PBS buffer, pH 7.4....60
S6.5. Tris buffer, pH 7.4....60
S6.6. Acetate buffer, pH 5.0....61
S7. ITC binding studies....62
S7.1. Concanavalin A (Con A)....62
S7.1.1. [**1**][BF_4]$_8$....63
S7.1.2. [**2**][BF_4]$_8$....64
S7.1.3. [**3**][BF_4]$_8$....65
S7.2. Griffithsin (GRFT)....66
S7.2.1. [**Fe_4L_4-12**][BF_4]$_8$....66
S7.2.2. [**1**][BF_4]$_8$....67
S7.2.3. [**2**][BF_4]$_8$....68
S7.2.4. [**3**][BF_4]$_8$....69
S8. Griffithsin Protein Expression and Purification....70
S9. References....71

S1. General considerations

S1.1. Materials

All manipulations performed under an inert atmosphere of N_2 were conducted in a Vacuum Atmospheres NexGen glovebox or using standard Schlenk techniques, and all other manipulations were performed under open atmosphere conditions in a fume hood. All reagents were purchased from ChemImpex, TCI, Thermo Fisher, AA Blocks, or Alfa Aesar, and used as received unless otherwise indicated. Tetrahydrofuran (THF) was purified on a JC MeyerGlass Contour Solvent Purification System, and all other solvents (dichloromethane (DCM), acetone, methanol (MeOH), *N*,*N*-dimethylformamide (DMF), ethyl acetate (EtOAc), hexanes, diethyl ether (Et_2O)) were used as received without further purification. 1,3,5-tris(4-aminophenyl)-1,3,5-triazinane-2,4,6-trione,[1] α/β-D-$(OAc)_5$-mannose,[2] and $(OAc)_4$-α-D-mannose(diethyleneglycol)Br[1] were prepared following previously reported procedures. Deuterated solvents ($CDCl_3$, DMSO-d_6, D_2O) were purchased from Cambridge Isotope Laboratories and used as received. Concanavalin A (Con A) was purchased from Thermo Scientific and stored at -20 °C prior to use. ITC buffers were prepared using Milli-Q water and solutions were filtered through a Corning® 0.22 µm pore sized syringe filter prior to use.

S1.2. Methods

All NMR spectra were obtained on 300 MHz Bruker AVA or 400 MHz JEOL FT NMR spectrometers, and ^1H and $^{13}C\{^1H\}$ NMR spectra were referenced to residual protio solvent signals. High-resolution mass spectrometry (HR-MS) analyses were performed at the Molecular MS Facility at UC San Diego using an Agilent 6230 time-of-flight mass spectrometer (TOFMS) with a Jet Stream electrospray ionization (ESI) source. The Jet Stream ESI source was operated with the following parameters: VCap: 3500 V; fragmentor voltage: 165 V; drying gas temperature: 325 °C, sheath gas temperature: 325 °C, drying gas flow rate: 7.0 L/min; sheath gas flow rate: 10 L/min; nebulizer pressure: 40 psi. Low-resolution mass spectrometry analyses were performed using a Waters Acquity UPLC Plus instrument equipped with a quadrupole time-of-flight mass spectrometer. UV-vis measurements were conducted using an Agilent Cary 60 UV-vis spectrophotometer equipped with a Xenon flashlamp (80 Hz) light source. Flash chromatography was performed on a CombiFlash NexGen 300+ system using 24 g columns packed with 230-400 mesh grade 60 silica. UV-vis measurements were carried out using quartz cuvettes (1 cm path length) and conducted at 25 °C with solution samples at 50 µM concentration unless otherwise noted. ATR-IR spectra were collected on an Agilent Cary 630 FTIR spectrometer. ITC measurements were performed on a VP-ITC (MicroCal Inc.) instrument and experimental details are provided in Section S6. Dynamic light scattering (DLS) measurements were conducted at 25 °C using a DynaPro Plate Reader III (Wyatt Technology) with a 150° scattering angle and 817 nm laser. Transmission electron microscopy (TEM) measurements were performed at the UCSD Cellular and Molecular Medicine Electron Microscopy Facility and experimental parameters are described in Section S4. Atomic force microscopy (AFM) measurements were acquired in tapping mode on a Bruker Innova instrument with a 160AC-NA cantilever (MikroMasch), and experimental parameters are described in Section S5.

S2. Synthetic procedures and characterization data for all compounds

S2.1. Organic subcomponents

S2.1.1. 2-(2-bromoethoxy)ethanol

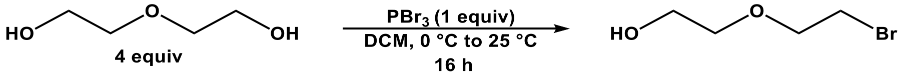

The preparation of 2-(2-bromoethoxy)ethanol was adapted from a literature procedure,[1] and spectroscopic characterization of the isolated product match the reported data.

In a 100 mL two-neck flask, to a solution of diethylene glycol (9.000 g, 84.81 mmol, 4.000 equiv) in DCM (20 mL) was added PBr_3 (2.0 mL, 21 mmol, 1.0 equiv) dropwise over the course of 20 min at 0 °C under an atmosphere of N_2. The reaction mixture was allowed to warm to ambient temperature and stirred for an additional 15 h. The solution was diluted with DCM (150 mL) and then washed with a saturated solution of $NaHCO_3$ (2 x 100 mL) followed by a saturated solution of NaCl (3 x 75 mL). The organic phase was removed and dried over $MgSO_4$. The solution was then filtered through a pad of Celite, and all volatiles were removed under reduced pressure. The crude product was purified by flash liquid chromatography on silica gel eluting with a solvent gradient of 70:30 to 50:50 hexanes/EtOAc to afford the pure product as a colorless liquid (yield: 1.600 g, 9.467 mmol, 68%). ^{1}H NMR (300 MHz, 25 °C, $CDCl_3$) δ: 3.83 (t, 2H, ^3J = 5.9 Hz), 3.76 (t, 2H, ^3J = 4.2 Hz), 3.63 (t, 2H, ^3J = 4.2 Hz), 3.50 (t, 2H, ^3J = 5.9 Hz), 1.93 (s, 1H, O*H*) ppm.

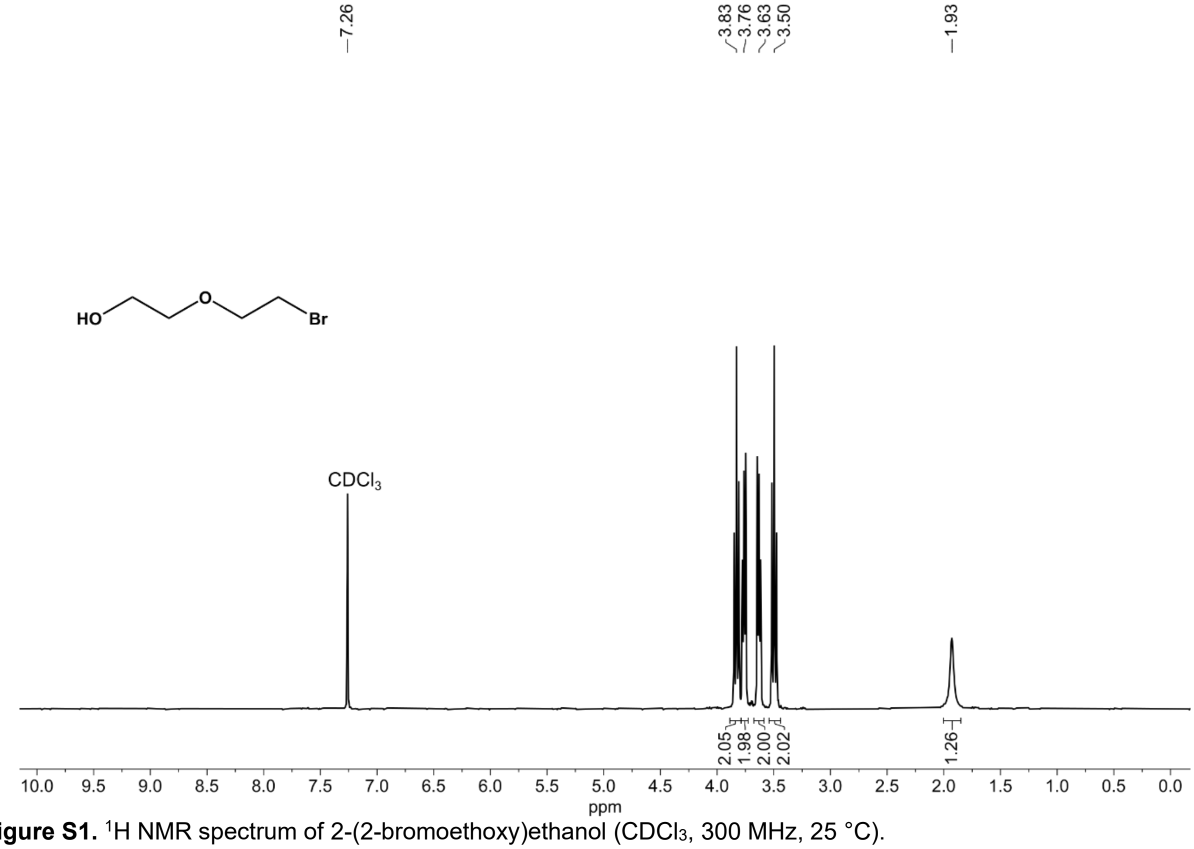

Figure S1. ^{1}H NMR spectrum of 2-(2-bromoethoxy)ethanol ($CDCl_3$, 300 MHz, 25 °C).

S2.1.2. 2-(2-azidoethoxy)ethyl(OAc)$_4$-α-D-mannose

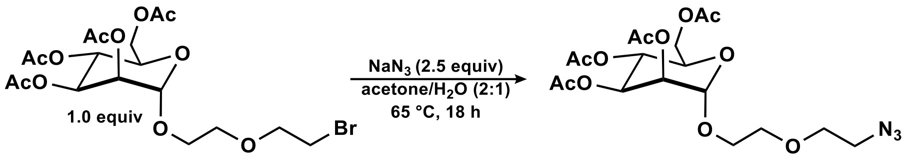

The preparation of 2-(2-azidoethoxy)ethyl(OAc)$_4$-α-D-mannose was adapted from the literature,[3] and spectroscopic characterization of the isolated product match the reported data.[4]

In a 200 mL round bottom flask, a solution of (OAc)$_4$-α-D-mannose(diethyleneglycol)Br (2.451 g, 5.545 mmol, 1.000 equiv) and NaN_3 (902 mg, 13.9 mmol, 2.50 equiv) in a 3:1 mixture of acetone/H_2O (80 mL total) was refluxed for 18 h under an atmosphere of N_2. The reaction mixture was allowed to cool to ambient temperature, diluted with EtOAc (150 mL), and washed with a saturated aqueous solution of NaCl (3 x 150 mL). The organic phase was dried over $MgSO_4$, and the solvent was evaporated under reduced pressure to yield the product as a colorless oil, which was used without further purification (2.220 g, 4.770 mmol, 86%). ^{1}H NMR (400 MHz, 25 °C, $CDCl_3$) δ: 5.37 (dd, 1H, J = 10.0, 3.2 Hz, H-3), 5.29 (t, 1H, J = 10.0 Hz, H-4), 5.28 (dd, 1H, J = 3.6, 1.6 Hz, H-2), 4.88 (d, 1H, J = 1.6 Hz, H-1), 4.29 (dd, 1H, J = 12.9, 5.3 Hz, H-7a), 4.12–4.06 (m, 2H, H-7b, H-5 signals overlapping), 3.87–3.79 (m, 1H, H-6$_a$), 3.69–3.66 (m, 5H, H-6$_b$, H-8, H-9), 3.39 (m, 2H, J = 4.9 Hz, H-10), 2.15 (s, 3H, OAc), 2.10 (s, 3H, OAc), 2.04 (s, 3H, OAc), 1.99 (s, 3H, OAc) ppm. ATR-IR (ν): 2937, 2881, 2110 (N_3), 1741 (C=O), 1439, 1368, 1251, 1133, 1081, 1044, 977, 921, 790, 600 cm^{-1}.

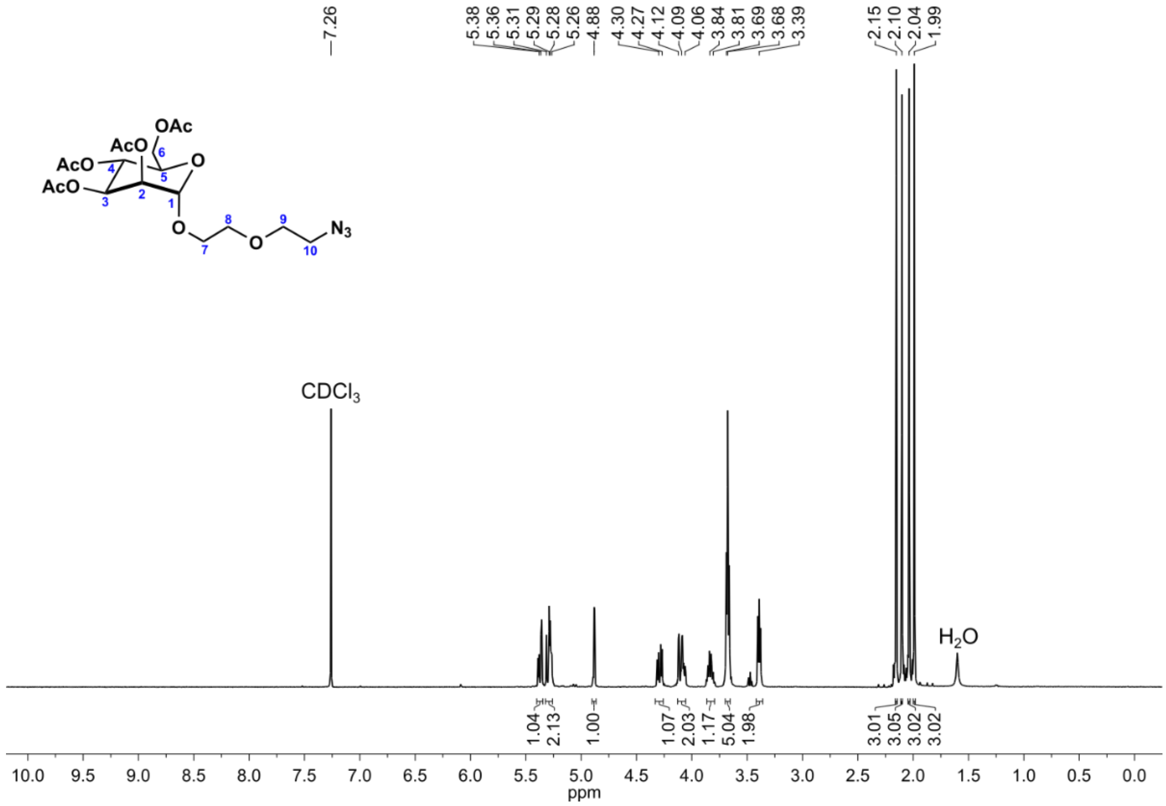

Figure S2. ^{1}H NMR spectrum of 2-(2-azidoethoxy)ethyl(OAc)$_4$-α-D-mannose ($CDCl_3$, 400 MHz, 25 °C).

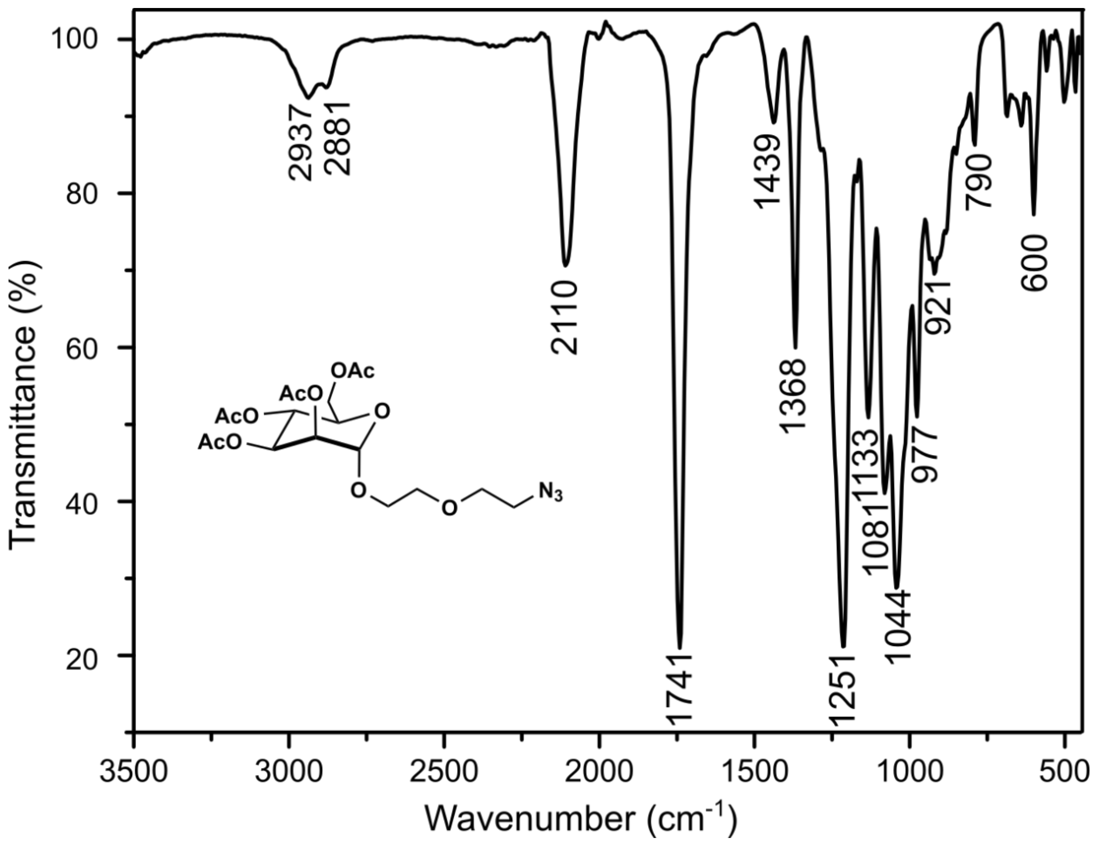

Figure S3. ATR-IR spectrum of 2-(2-azidoethoxy)ethyl(OAc)$_4$-α-D-mannose.

S2.1.3. 3,5-dihydroxybenzyl alcohol

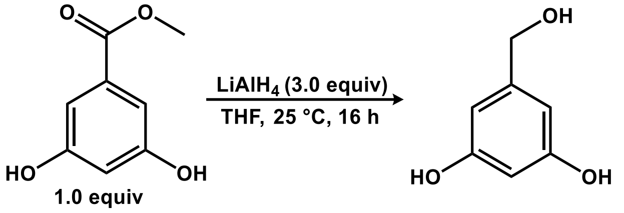

The synthesis of 3,5-dihydroxybenzyl alcohol was adapted from a procedure reported in the litertature,[5] and spectroscopic characterization of the product match the reported data.[6]

In a 200 mL Schlenk flask in the glovebox, a solution of methyl 3,5-dihydroxymethylbenzoate (3.000 g, 17.85 mmol, 1.000 equiv) in THF (50 mL) was added dropwise to a suspension of $LiAlH_4$ (2.000 g, 53.56 mmol, 3.000 equiv) in THF (20 mL) over the course of 20 min at -35 °C. The reaction was allowed to warm to glovebox temperature and stirred for a total of 16 h, at which point the $LiAlH_4$ was quenched with H_2O (3 mL) and then neutralized with HCl (1 M). To the mixture was added EtOAc (200 mL), and the organic phase was washed with a saturated aqueous solution of NaCl (3 x 150 mL). T he organic phase was dried over $MgSO_4$, filtered through a pad of Celite and evaporated to dryness. The crude product was suspended in a solution of 60/40 EtOAc/hexanes and isolated by filtration to yield 3,5-dihydroxybenzyl alcohol as a colorless solid (2.100 g, 14.99 mmol, 84%). M.p. 184–187 °C. ^1H NMR (300 MHz, 25 °C, DMSO-d_6) δ: 9.10 (s, 2H, Ar-O*H*), 6.15 (d, 2H, J = 1.9 Hz, *o*-Ar-*H*), 6.04 (t, 1H, J = 1.9 Hz, *p*-Ar-*H*), 5.01 (t, 1H, J = 5.9 Hz, CH_2-O*H*), 4.29 (d, 2H, J = 5.8 Hz, C*H*$_2$-OH) ppm.

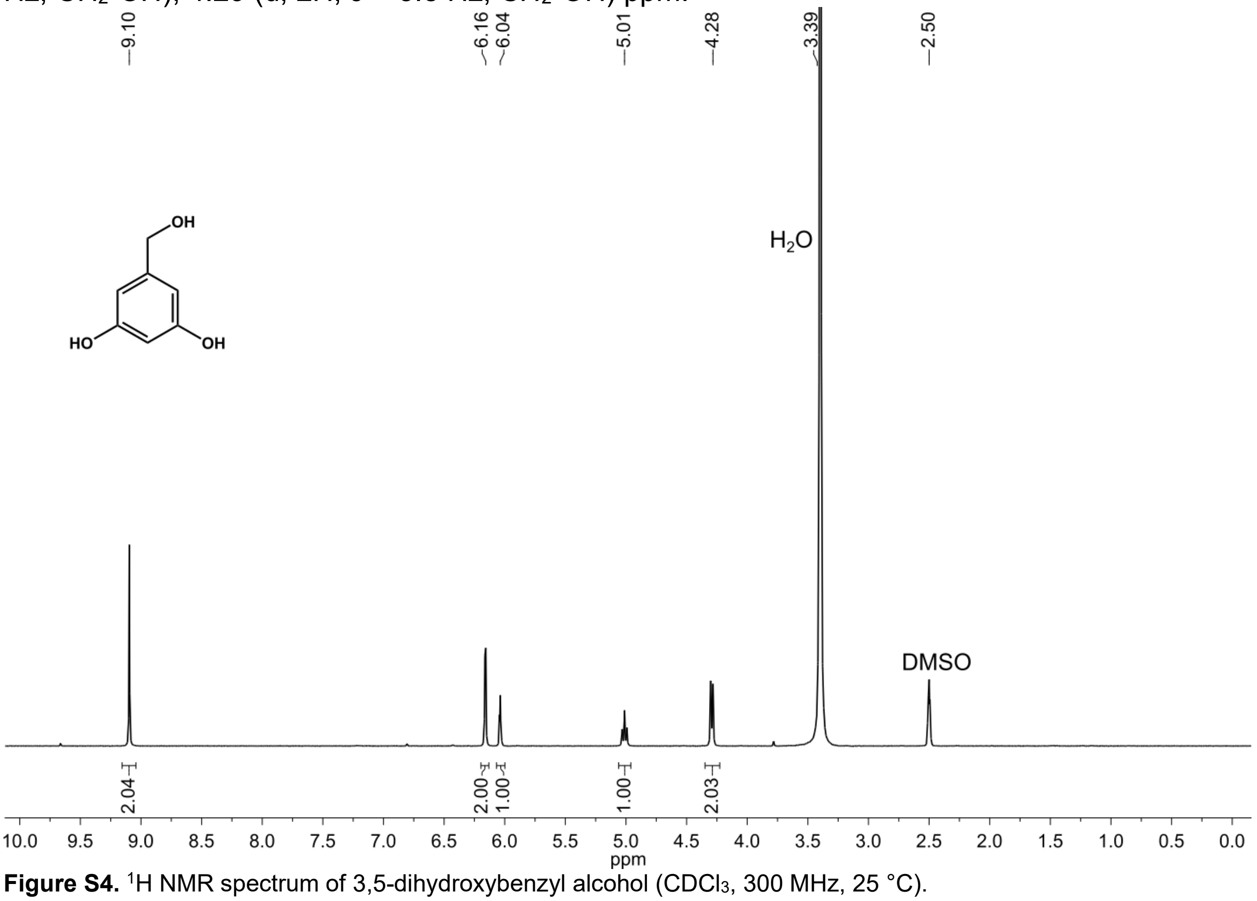

Figure S4. ^1H NMR spectrum of 3,5-dihydroxybenzyl alcohol ($CDCl_3$, 300 MHz, 25 °C).

S2.1.4. 3,5-bis(propynyloxy)benzoate

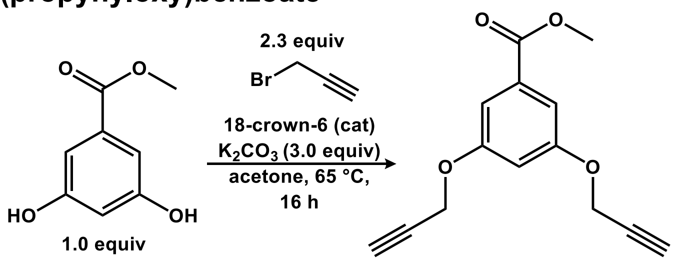

The synthesis of methyl 3,5-bis(propynyloxy)benzoate was adapted from the literature,[7] and spectroscopic characterization of the product match the reported data.[8]

To a solution of methyl 3,5-dihydroxybenzoate (4.000 g, 23.79 mmol, 1.000 equiv), K_2CO_3 (19.700 g, 142.74 mmol, 6.000 equiv) and 18-crown-6 (314 mg, 1.19 mmol, 0.05 equiv) in acetone (70 mL) was added a solution of propargyl bromide (5.95 mL of a 9.2 M solution, 54.7 mmol, 2.30 equiv) and the suspension was heated at reflux for 16 h. The reaction mixture was then allowed to cool to ambient temperature and diluted with EtOAc (250 mL). The mixture was washed with a saturated aqueous solution of NaCl (3 x 150 mL), and the organic phase was dried over $MgSO_4$ and filtered through a pad of Celite. The filtrate was evaporated to dryness to yield methyl 3,5-bis(propynyloxy)benzoate as a colorless powder, which was used in the next step without further purification (5.624 g, 23.04 mmol, 97%). M.p. 108–111 °C. ^{1}H NMR (300 MHz, 25 °C, $CDCl_3$) δ: 7.30 (d, 2H, *J* = 2.4 Hz, *o*-Ar-*H*), 6.81 (t, 1H, *J* = 2.4 Hz, *p*-Ar-*H*), 4.72 (d, 4H, *J* = 2.4 Hz, C*H*$_2$), 3.91 (s, 3H, C*H*$_3$), 2.54 (t, 2H, *J* = 2.4 Hz, C≡C*H*) ppm.

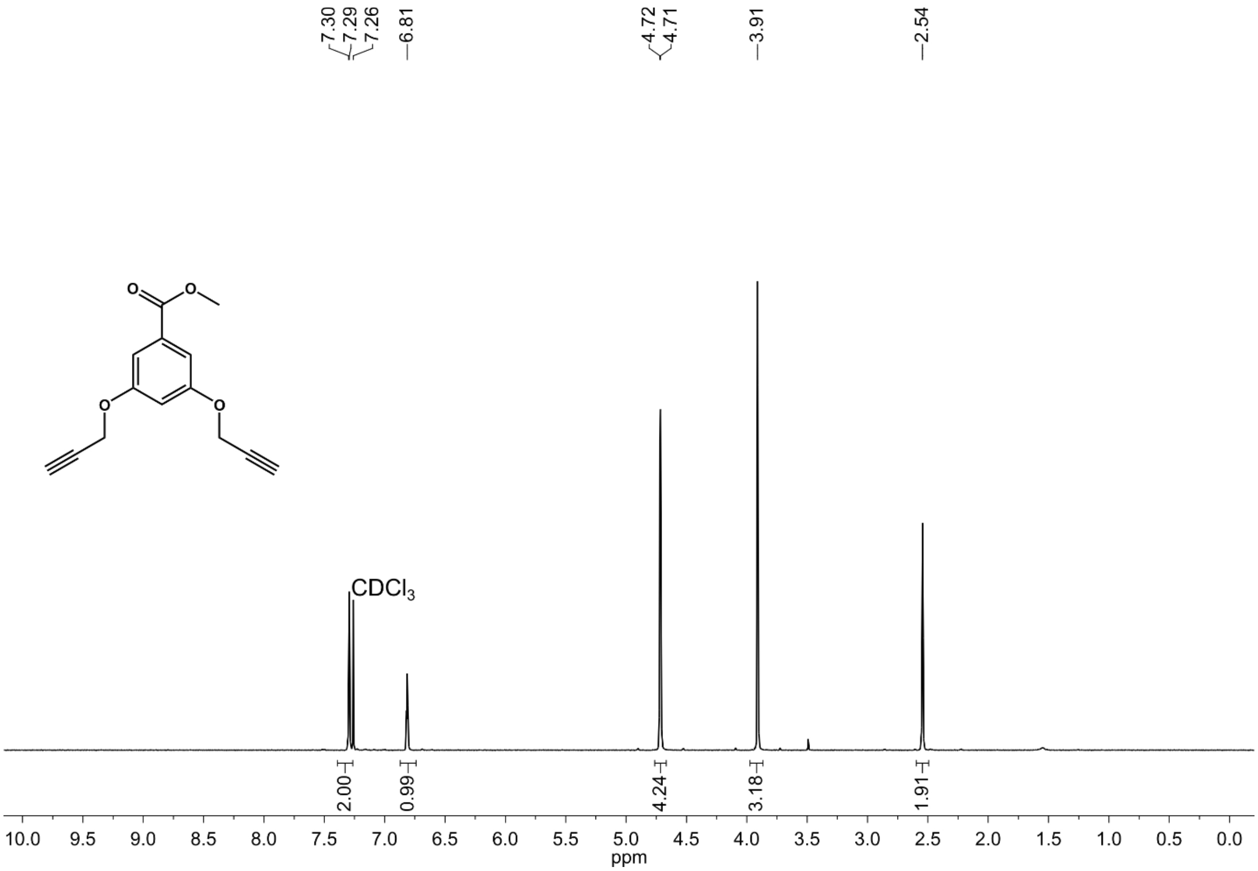

Figure S5. ^{1}H NMR spectrum of 3,5-bis(propynyloxy)benzoate ($CDCl_3$, 400 MHz, 25 °C).

S2.1.5. 5-(hydroxymethyl)-1,3-bis(propynyloxy)benzene

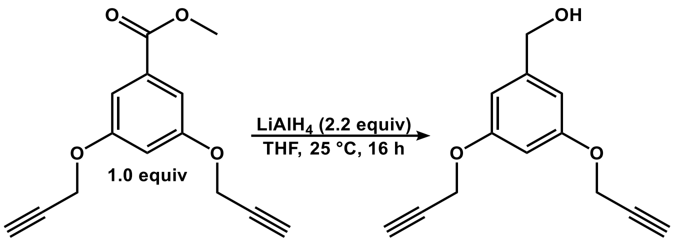

The synthesis of 5-(hydroxymethyl)-1,3-bis(propynyloxy)benzene was adapted from the literature, and spectroscopic characterization of the product match the reported data.[9]

In a 200 mL Schlenk flask in the glovebox, a solution of methyl 3,5-bis(propynyloxy)benzoate (5.624 g, 23.03 mmol, 1.000 equiv) in THF (30 mL) was added dropwise to a suspension of $LiAlH_4$ (1.925 g, 50.66 mmol, 2.200 equiv) in THF (50 mL) over the course of 20 min at -35 °C. The reaction was allowed to warm to glovebox temperature and stirred for a total of 16 h, at which point the $LiAlH_4$ was quenched with H_2O (1 mL), NaOH (1 mL of a 15 wt% solution), and H_2O (10 mL) sequentially. To the mixture was added EtOAc (200 mL), and the organic phase was washed with a saturated aqueous solution of NaCl (3 x 150 mL). The organic phase was dried over $MgSO_4$, filtered through a pad of Celite and evaporated to dryness to yield the crude product as a brown oil, which was purified by flash liquid chromatography on silica gel eluting with a solvent gradient of 30:70 to 50:50 EtOAc/hexanes to yield 5-(hydroxymethyl)-1,3-bis(propynyloxy)benzene as a colorless powder (3.937 g, 16.12 mmol, 70%). M.p. 98–101 °C. ^{1}H NMR (300 MHz, 25 °C, $CDCl_3$) δ: 6.63 (d, 2H, *J* = 2.1 Hz, *o*-Ar-*H*), 6.54 (t, 2H, *J* = 2.1 Hz, *p*-Ar-*H*), 4.68 (d, 2H, *J* = 2.4 Hz, OC*H*$_2$), 4.65 (s, 2H, *J* = 5.9 Hz, C*H*$_2$OH), 2.53 (t, 2H, *J* = 2.4 Hz, C≡*H*), 1.69 (s, 1H, CH_2O*H*) ppm.

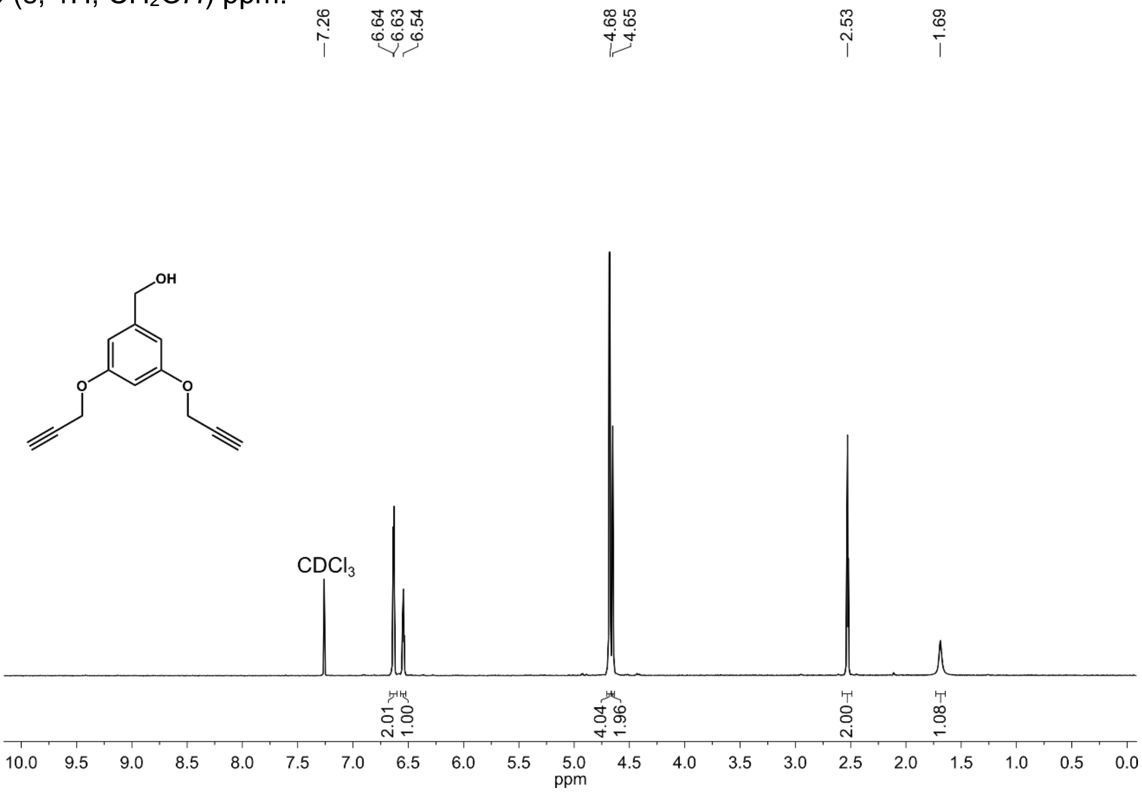

Figure S6. ^{1}H NMR spectrum of 5-(hydroxymethyl)-1,3-bis(propynyloxy)benzene ($CDCl_3$, 300 MHz, 25 °C).

S2.1.6. 5-(bromomethyl)-1,3-bis(propynyloxy)benzene

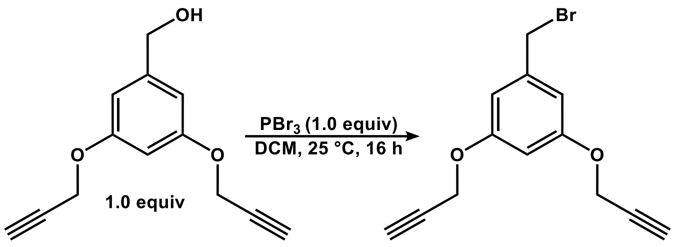

The synthesis of 5-(bromomethyl)-1,3-bis(propynyloxy)benzene was adapted from the literature, and spectroscopic characterization of the product match the reported data.[10]

In a 200 mL two-neck flask under an atmosphere of N_2, PBr_3 (2.2 mL, 23 mmol, 1.0 equiv) was added to a solution of 5-(hydroxymethyl)-1,3-bis(propynyloxy)benzene (4.941 g, 22.85 mmol, 1.000 equiv) in DCM (100 mL) at 0 °C dropwise over the course of 20 min. The reaction mixture was allowed to warm to ambient temperature, and then stirred for an additional 16 h. The mixture was diluted with DCM (150 mL) and washed with a saturated aqueous solution of $NaHCO_3$ (2 x 150 mL), followed by a saturated aqueous solution of NaCl (3 x 150 mL). The organic phase was dried over $MgSO_4$, filtered through a pad of Celite and evaporated to dryness. The crude product was redissolved in a minimal amount of DCM and precipitated with hexanes to yield 5-(bromomethyl)-1,3-bis(propynyloxy)benzene as a colorless solid (6.378 g, 15.92 mmol, 77%). M.p. 66–68 °C. ^1H NMR (300 MHz, 25 °C, $CDCl_3$) δ: 6.65 (d, 2H, J = 2.2 Hz, *o*-Ar-*H*), 6.56 (t, 2H, J = 2.2 Hz, *p*-Ar-*H*), 4.68 (d, 2H, J = 2.4 Hz, OCH_2), 4.42 (s, 2H, CH_2Br), 2.54 (t, 2H, J = 2.4 Hz, C≡*H*) ppm.

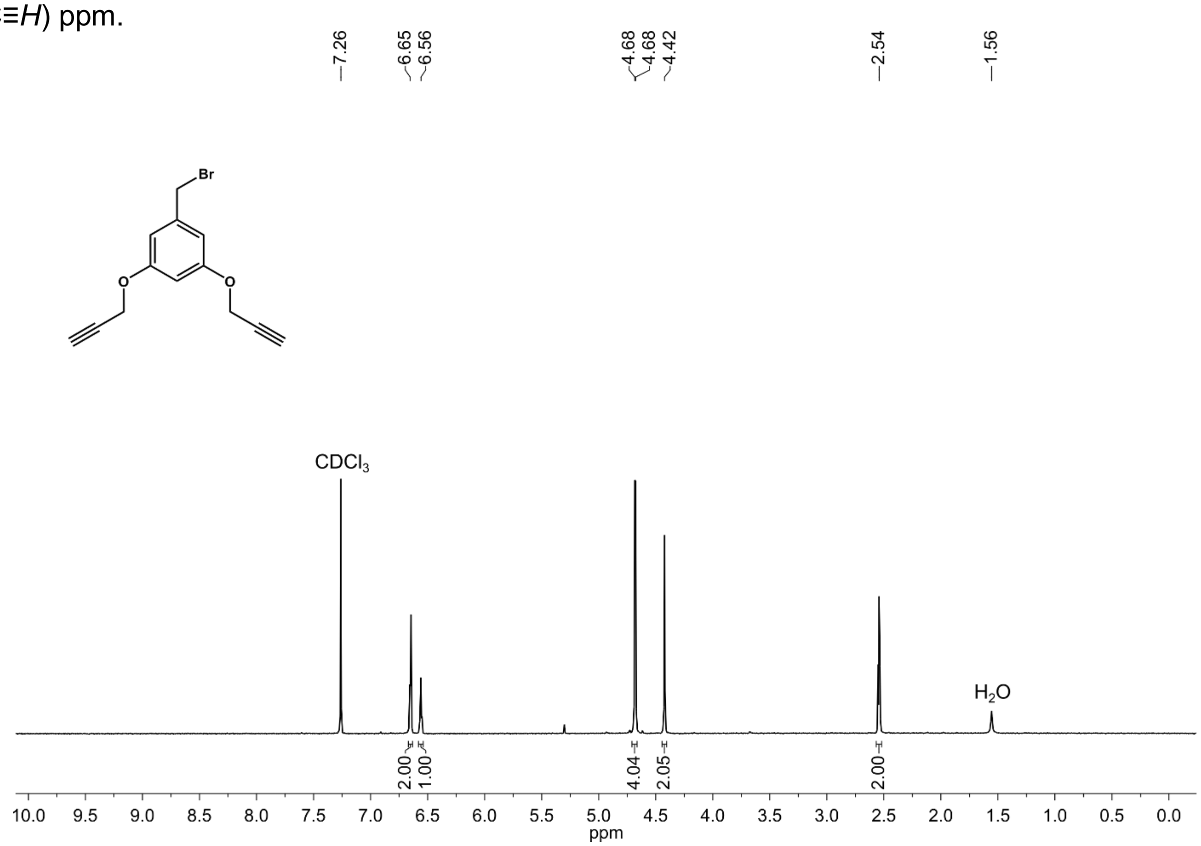

Figure S7. ^1H NMR spectrum of 5-(bromomethyl)-1,3-bis(propynyloxy)benzene ($CDCl_3$, 300 MHz, 25 °C).

S2.1.7. 5-((3,5-bis(propynyloxy)benzyl)oxy)picolinaldehyde (a)

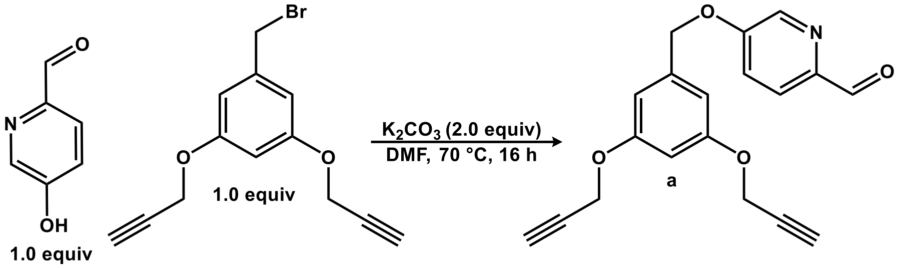

In a 50 mL Schlenk flask under an atmosphere of N_2, a DMF (10 mL) solution of 5-(bromomethyl)-1,3-bis(propynyloxy)benzene (500 mg, 1.79 mmol, 1.00 equiv), 5-hydroxypicolinaldehyde (220 mg, 1.79 mmol, 1.00 equiv), and K_2CO_3 (494 mg, 3.58 mmol, 2.00 equiv) was heated at 70 °C for 16 h. The reaction mixture was then allowed to cool to ambient temperature, diluted with EtOAc (150 mL), and washed with a saturated aqueous solution of NaCl (3 x 150 mL). The organic phase was dried over $MgSO_4$, filtered through a pad of Celite and evaporated to dryness to yield a brown oil, which was purified by flash liquid chromatography on silica gel eluting with a solvent gradient of 30:70 to 60:40 EtOAc/hexanes to yield 5-((3,5-bis(propynyloxy)benzyl)oxy)picolinaldehyde (a) as a colorless powder (422 mg, 1.31 mmol, 73%). M.p. 102–105 °C. ^{1}H NMR (400 MHz, 25 °C, $CDCl_3$) δ: 10.00 (s, 1H, OC*H*), 8.49 (d, 1H, ^4J = 2.7 Hz, py-*H*-6), 7.95 (d, 1H, ^3J = 8.6 Hz, py-*H*-3), 7.35 (dd, 1H, ^3J = 8.6 Hz, ^4J = 2.7 Hz, py-*H*-4), 6.68 (d, 2H, ^4J = 2.2 Hz, *o*-Ar-*H*), 6.60 (t, 1H, ^4J = 2.2 Hz, *p*-Ar-H), 5.16 (s, 2H, C*H*$_2$-O-py), 4.69 (d, 4H, ^4J = 2.4 Hz, C*H*$_2$C≡CH), 2.52 (t, 2H, ^4J = 2.4 Hz, C≡CH) ppm. ^{13}C{^{1}H} NMR (100 MHz, 25 °C, $CDCl_3$) δ: 192.0 (O*C*H), 159.0 (*m*-Ar-*C*), 158.0 (py-*C*-2), 146.5 (py-*C*-5), 139.1 (py-*C*-6), 137.7 (Ar-*C*H$_2$), 123.4 (py-*C*-3), 121.1 (py-*C*-4), 106.7 (*o*-Ar-*C*), 102.1 (*p*-Ar-*C*), 78.2 (*C*≡CH), 76.0 (C≡*C*H), 70.3 (*C*H$_2$-O-py), 56.0 (O-*C*H$_2$) ppm. ATR-IR (ν): 3269, 2119 (C≡C), 1700 (HC=O), 1573, 1446, 1379, 1316, 1215, 1069, 1032, 910, 828, 708, 652 cm^{-1}.

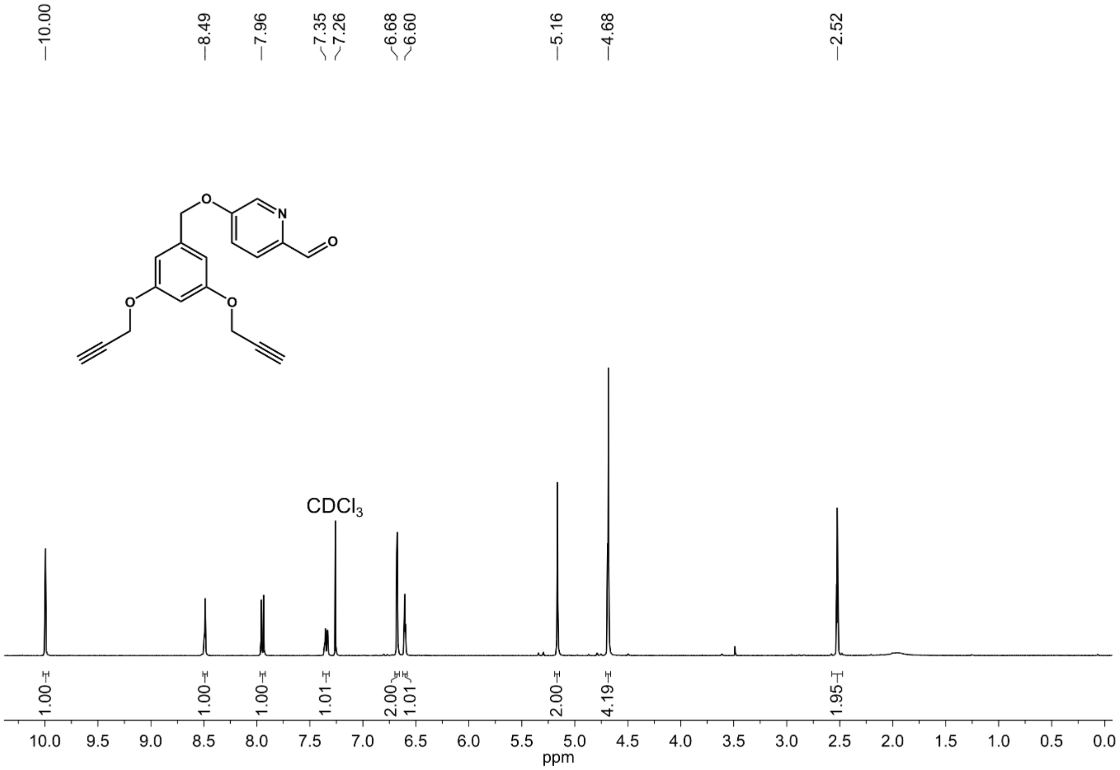

Figure S8. ^{1}H NMR spectrum of 5-((3,5-bis(propynyloxy)benzyl)oxy)picolinaldehyde ($CDCl_3$, 400 MHz, 25 °C).

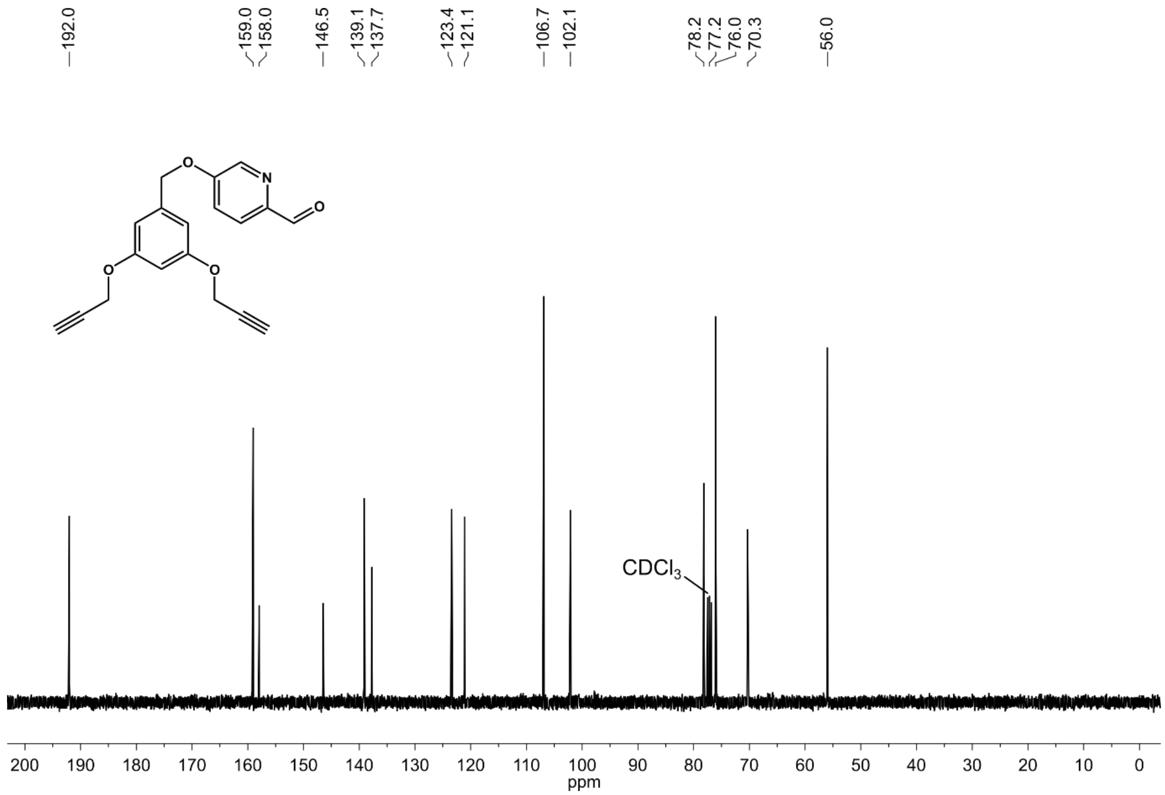

Figure S9. $^{13}C\{^1H\}$ NMR spectrum of 5-((3,5-bis(propynyloxy)benzyl)oxy)picolinaldehyde (CDCl₃, 100 MHz, 25 °C).

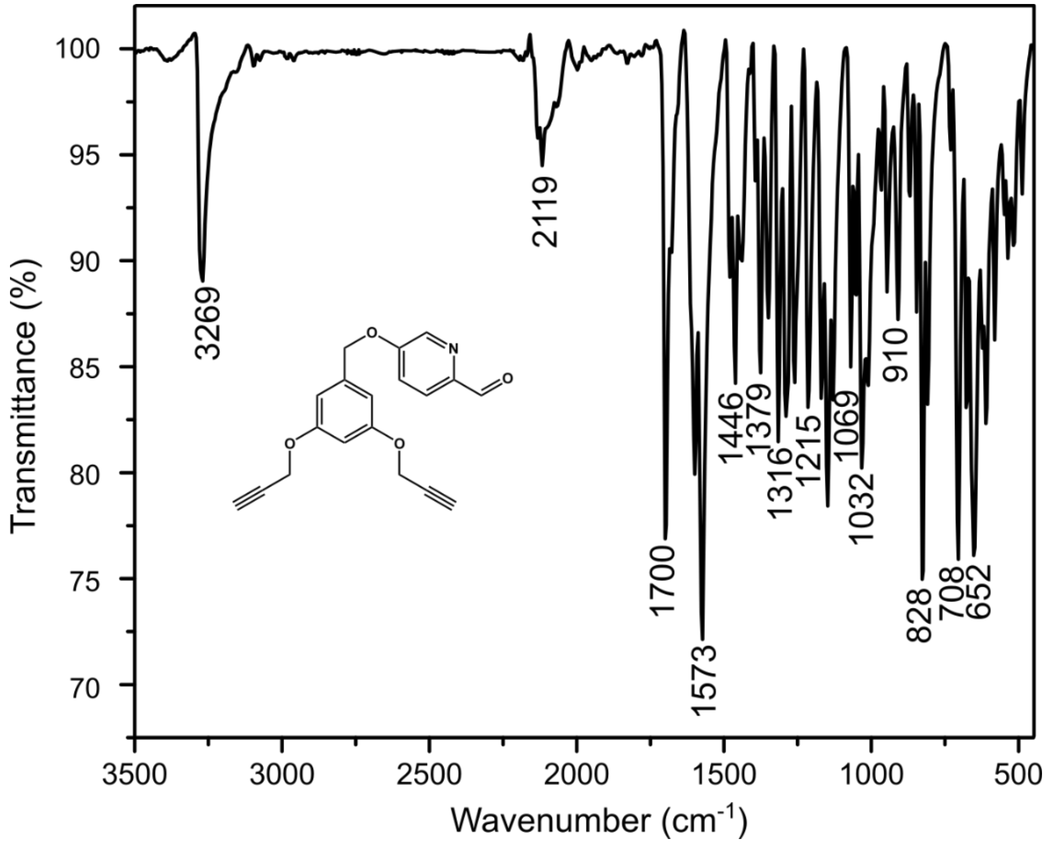

Figure S10. ATR-IR spectrum 5-((3,5-bis(propynyloxy)benzyl)oxy)picolinaldehyde.

S2.1.8. Man-2(OAc)

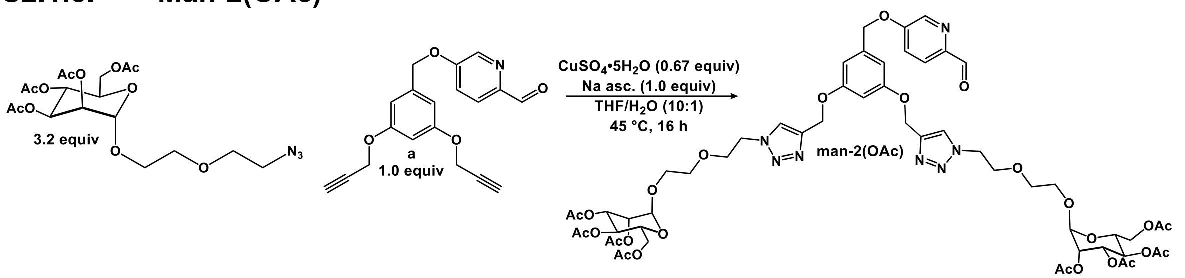

To a solution of $CuSO_4$•$5H_2O$ (78 mg, 0.31 mmol, 0.67 equiv) in H_2O (1 mL) in a 25 mL Schlenk flask was added a solution of 5-((3,5-bis(propynyloxy)benzyl)oxy)picolinaldehyde (a) (150 mg, 0.467 mmol, 1.00 equiv), sodium ascorbate (92 mg, 0.47 mmol, 1.0 equiv), and 2-(2-azidoethoxy)ethyl(OAc)$_4$-α-D-mannose (623 mg, 1.49 mmol, 3.20 equiv) in THF (10 mL). The yellow heterogeneous reaction mixture was allowed to stir under an atmosphere of N_2 for 16 h, at which point it was allowed to cool to ambient temperature. The mixture was diluted with EtOAc (150 mL) and washed with an aqueous solution of Na_4[EDTA] (3 x 30 mL), followed by a saturated aqueous solution of NaCl (3 x 150 mL). The organic phase was dried over $MgSO_4$, filtered through a pad of Celite and evaporated to dryness to yield a yellow oil that was first purified on silica gel eluting with a 60:40 EtOAc/hexanes mixture (100 mL) to remove unreacted 2-(2-azidoethoxy)ethyl(OAc)$_4$-α-D-mannose followed by acetone (50 mL). The acetone fraction was evaporated, and the residue was purified by flash liquid chromatography eluting with a solvent system of 0:100 to 5:95 MeOH/DCM to yield man-2(OAc) as a colorless powder (330 mg, 0.265 mmol, 57%). M.p. 47–49 °C. ^{1}H NMR (400 MHz, 25 °C, $CDCl_3$) δ: 9.96 (s, 1H, OC*H*), 8.45 (d, 1H, ^4J = 2.4 Hz, py-*H*-6), 7.92 (d, 1H, ^3J = 8.6 Hz, py-*H*-3), 7.77 (s, 2H, N-C*H*), 7.32 (dd, 1H, ^3J = 8.6 Hz, ^4J = 2.4 Hz, py-*H*-4), 6.68 (d, 2H, ^4J = 2.2 Hz, *o*-Ar-*H*), 6.64 (t, 1H, *p*-Ar-*H*), 5.31 (dd, 2H, *J* = 10.0, 3.3 Hz, man-*H*-3), 5.27 (d, 2H, *J* = 10.1 Hz, man-*H*-2), 5.23 (dd, 2H, man-*H*-4, *J* = 3.3, 1.8 Hz), 5.16 (s, 4H, *m*-CH_2), 5.12 (s, 2H, CH_2-Opy), 4.83 (d, 2H, *J* = 1.8 Hz, man-*H*-1), 4.51 (t, 4H, *J* = 5.8 Hz, OCH_2), 4.25 (dd, 2H, *J* = 12.2, 5.2 Hz, man-*H*-5), 4.08 (dd, 2H, man-*H*-6_a, *J* = 12.2, 2.8 Hz), 3.97 (dddd, 2H, *J* = 9.6, 5.2, 2.8 Hz, man-*H*-6_b), 3.87 (t, 4H, OCH_2), 3.79-3.72 (m, 2H, OCH_2), 3.60 (m, 6H, OCH_2), 2.12 (s, 6H, CH_3COO), 2.06 (s, 6H, CH_3COO), 2.01 (s, 6H, CH_3COO), 1.94 (s, 6H, CH_3COO) ppm. ^{13}C{^{1}H} NMR (100 MHz, 25 °C, $CDCl_3$) δ: 191.8 (O*C*H), 170.4 (CH_3*C*OO), 169.9 (CH_3*C*OO), 169.8 (CH_3*C*OO), 169.6 (CH_3*C*OO), 159.7 (py-*C*-2), 157.8 (*m*-Ar-*C*), 146.2 (py-*C*-5), 143.3 (C=CN), 139.0 (py-*C*-6), 137.5 (*p*-Ar-*C*), 124.0 (N-*C*=C), 123.3 (py-*C*-3), 120.9 (py-*C*-4), 106.4 (*o*-Ar-*C*), 101.6 (*p*-Ar-*C*), 97.4 (man-*C*-1), 70.2 (*C*H_2-O-py), 69.8 (O*C*H_2), 69.3 (O*C*H_2), 69.3 (O*C*H_2), 68.8 (man-*C*-2), 68.3 (man-*C*-3), 67.0 (man-*C*-5), 65.9 (man-*C*-4), 62.3 (man-*C*-6), 61.7 (OCH_2), 50.2 (ArO-*C*H_2), 20.7 (CH_3*C*OO), 20.6 (CH_3*C*OO), 20.53 (CH_3*C*OO), 20.49 (CH_3*C*OO) ppm (*two decimal places are given only where needed to distinguish resonances*). HR-ESI-MS(+) (*m/z*): 1266.4299 ([M+Na]$^+$, calc'd, 1266.4190). ATR-IR (*ν*): 2937, 2881, 1741 (HC=O), 1703 (C=O), 1577, 1457, 1372, 1215, 1044, 835, 731, 596 cm^{-1}.

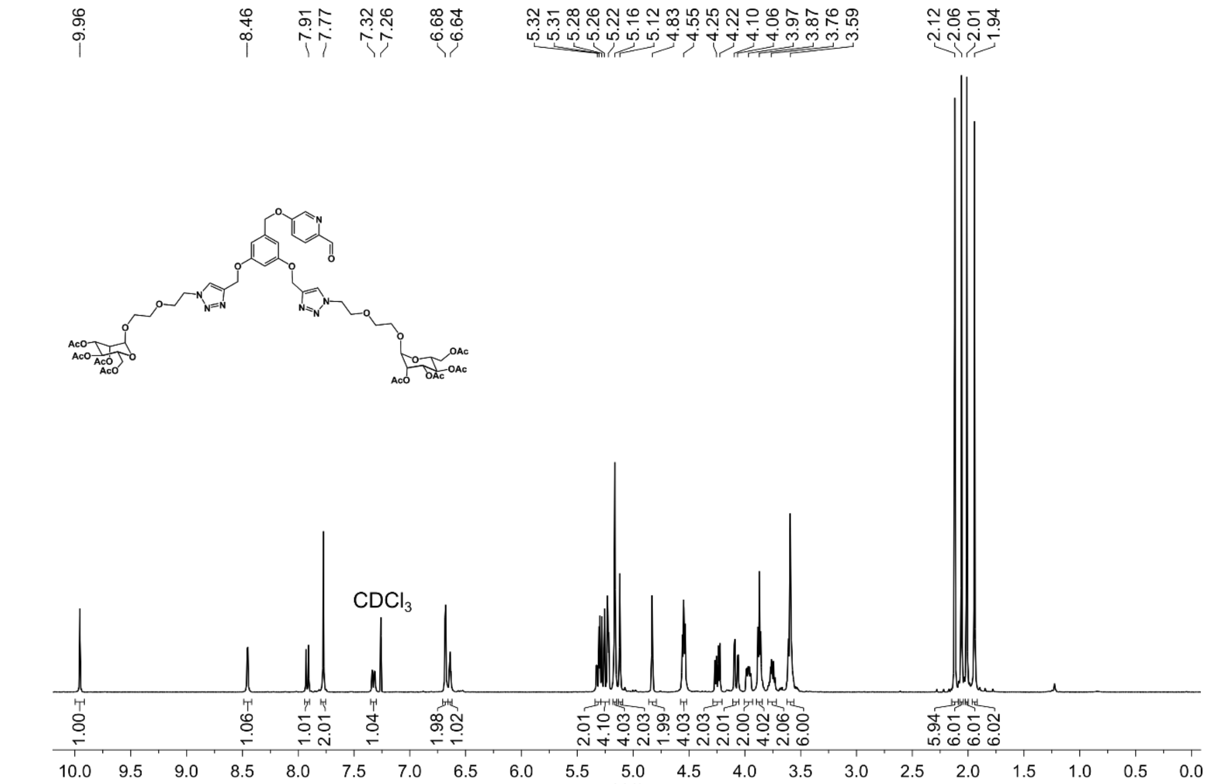

Figure S11. ^{1}H NMR spectrum of man-2(OAc) ($CDCl_3$, 400 MHz, 25 °C).

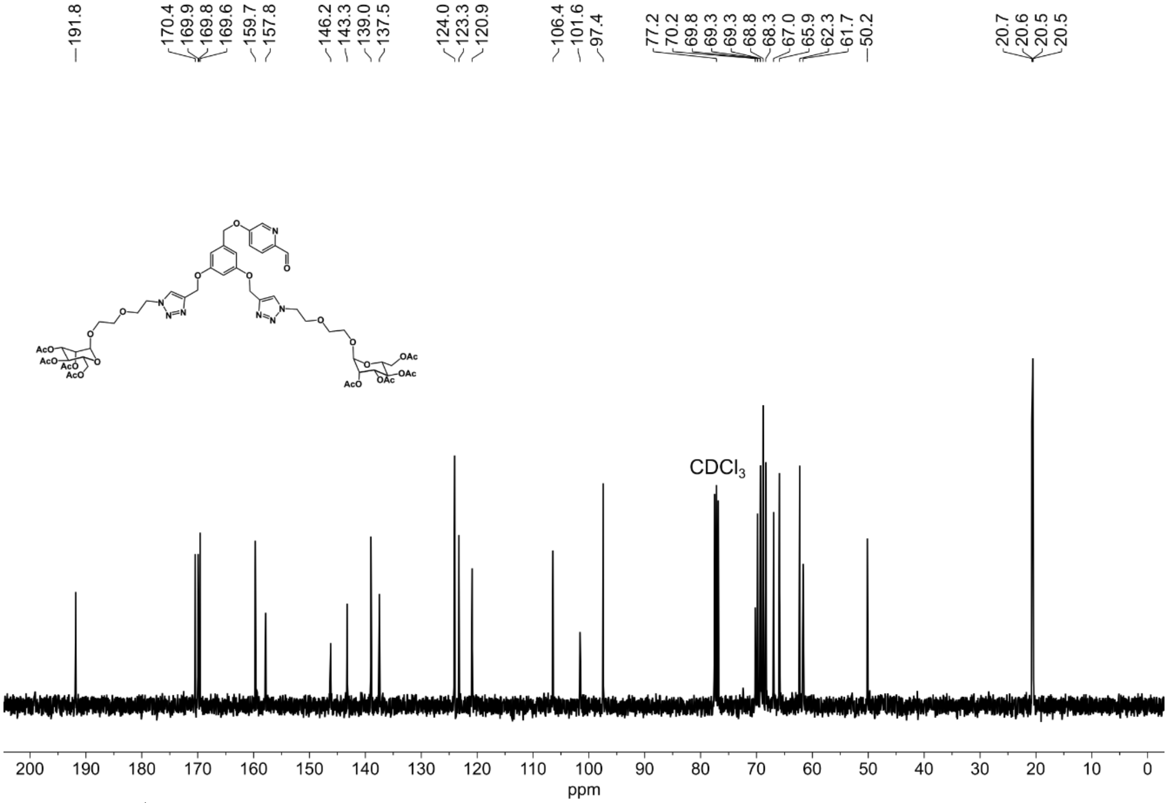

Figure S12. ^{1}H NMR spectrum of man-2(OAc) ($CDCl_3$, 100 MHz, 25 °C).

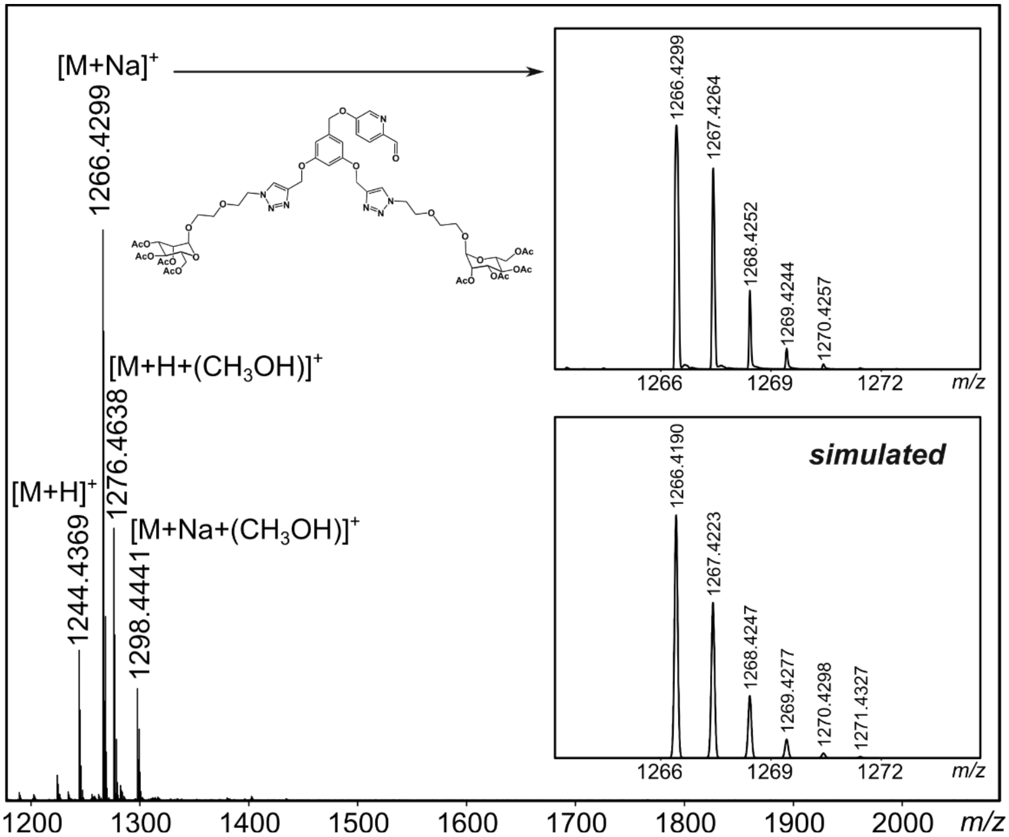

Figure S13. HR-ESI-MS(+) of man-2(OAc) (measured in CH_3OH).

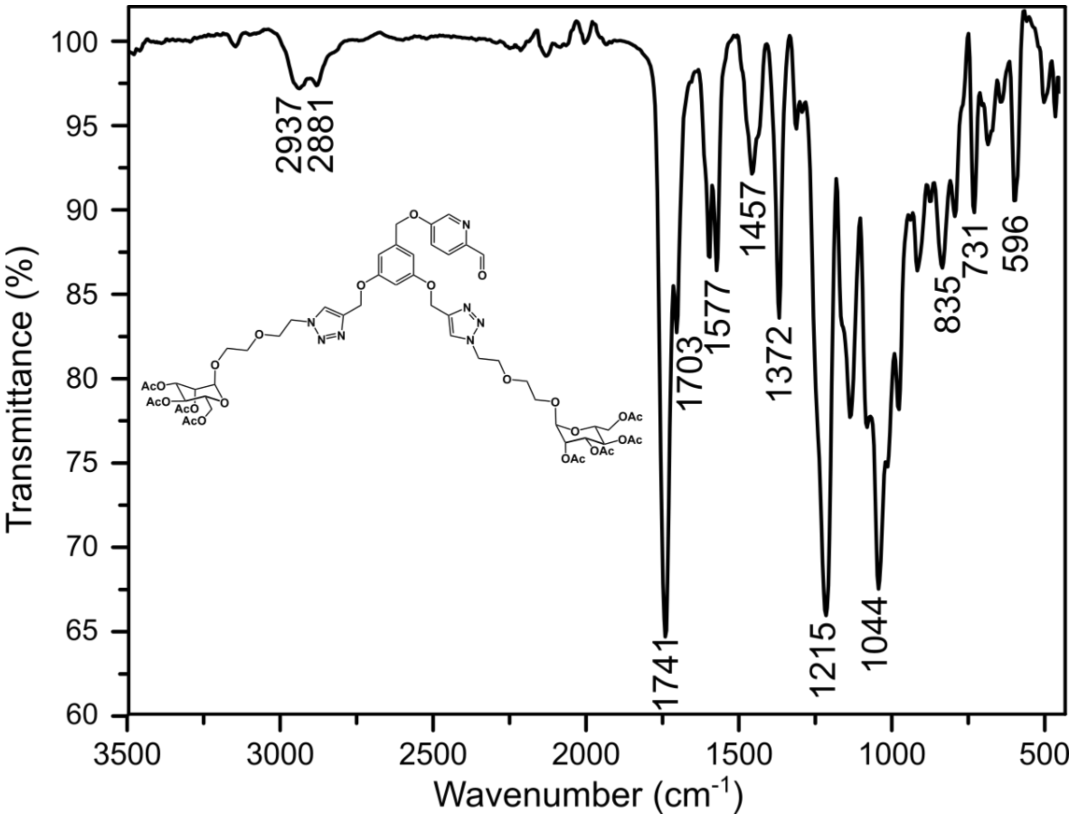

Figure S14. ATR-IR spectrum of man-2(OAc).

S2.1.9. Man-2

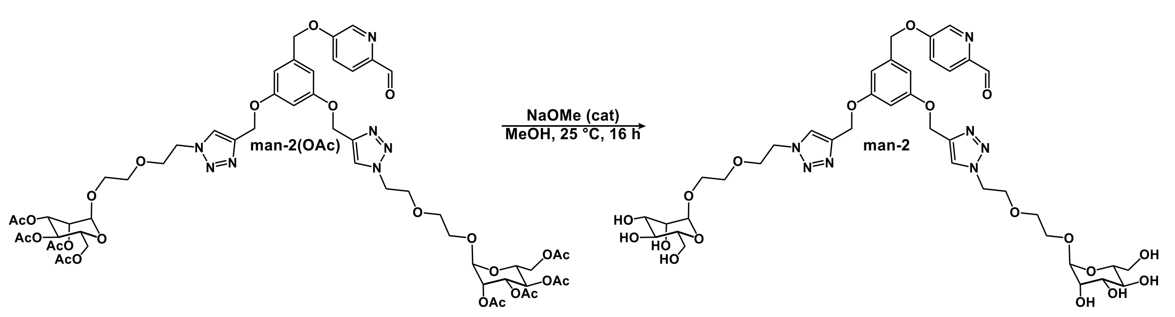

To a stirring solution of man-2(OAc) (84 mg, 0.067 mmol, 1.0 equiv) in MeOH (10 mL) was added a solution of NaOMe in MeOH until the pH reached 8 (500 µL of a 0.3 M solution, 0.150 mmol, 2.23 equiv). The reaction mixture was allowed to stir at ambient temperature under an atmosphere of N_2 for 16 h, at which point it was neutralized with Amberlyst®-15 (180 mg). The solution was filtered through a pad of Celite, and all volatiles were removed under reduced pressure to afford man-2 as a colorless solid that was used without further purification (yield: 48 mg, 0.054 mmol, 80%). ^{1}H NMR (400 MHz, 25 °C, D_2O) δ: 9.55 (s, 1H, OC*H*), 8.08 (s, 1H, py-*H*-6), 7.92 (s, 2H, N-C*H*), 7.56 (d, 1H, ^3J = 6.9. Hz, py-*H*-3), 7.10 (d, 1H, ^3J = 6.9 Hz, ^4J = 2.8 Hz, py-*H*-4), 6.40 (d, 2H, ^4J = 2.7 Hz, *o*-Ar-*H*), 6.33 (s, 1H, *p*-Ar-*H*), 4.88 (d, 2H, *J* = 12.5 Hz, man-*H*-1), 4.76 (s, 2H, –CH_2–), 4.66 (s, 2H, –CH_2–), 4.43 (s, 4H, *m*-CH_2), 3.79-3.73 (m, 8H, man-*H*-6_b, man-*H*-6_a man-*H*-2, man-*H*-3 signals overlapping), 3.69–3.56 (m, 8H, CH_2-Opy, man-*H*-4, man-*H*-5, –CH_2– signals overlapping), 3.48-3.38 (m, 10H, –CH_2–) ppm. ESI-MS(+) (*m/z*): 908.53 ([M+H]$^+$, calc'd, 908.35).

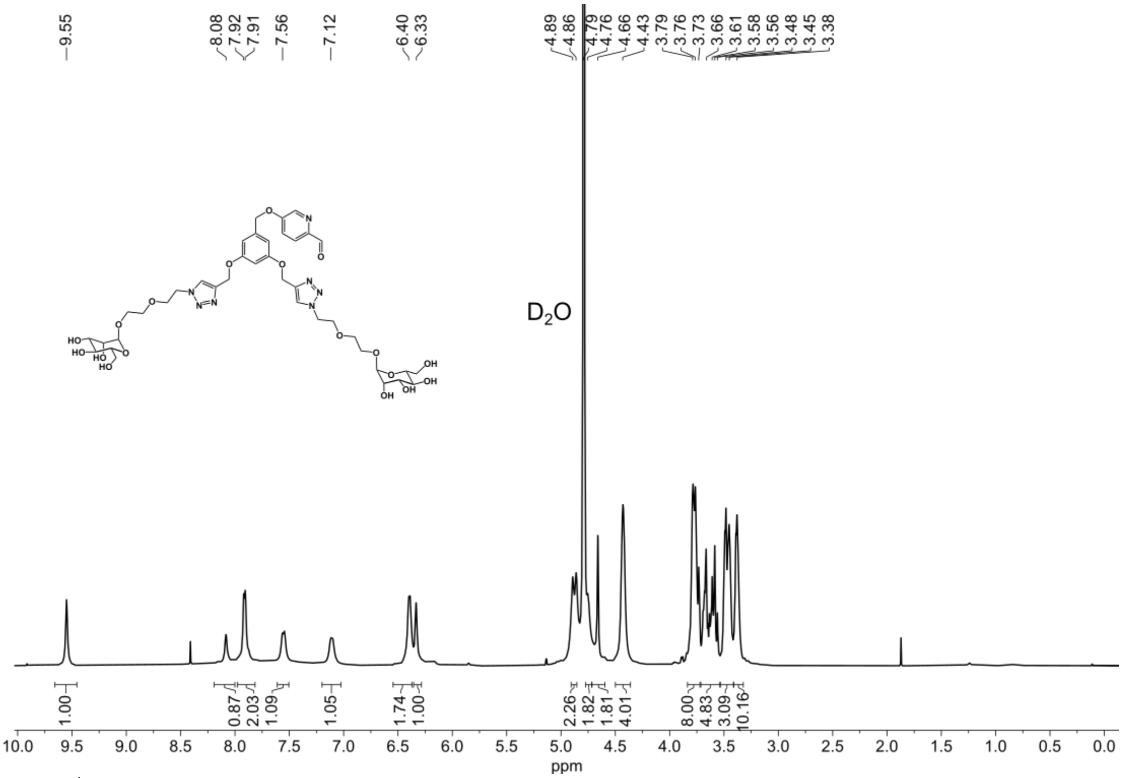

Figure S15. ^{1}H NMR spectrum of man-2 (D_2O, 400 MHz, 25 °C).

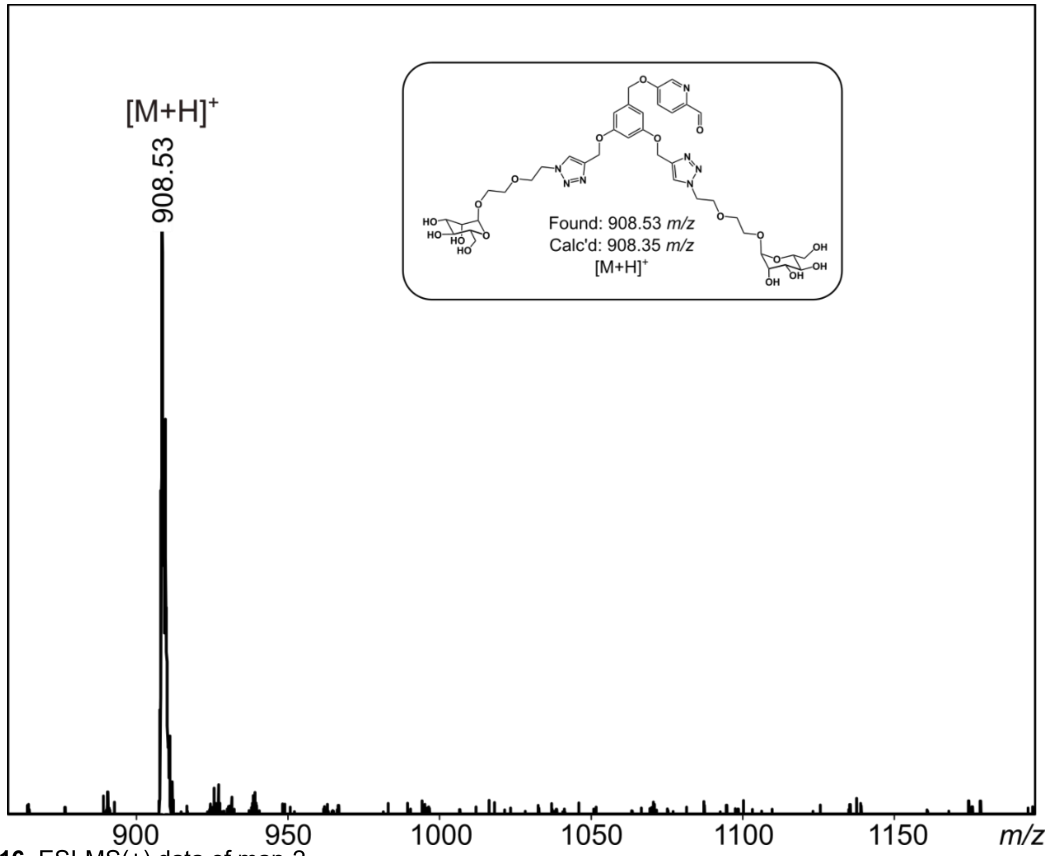

Figure S16. ESI-MS(+) data of man-2.

S2.1.10. Methyl 1,2,3-tris(propynyloxy)benzoate

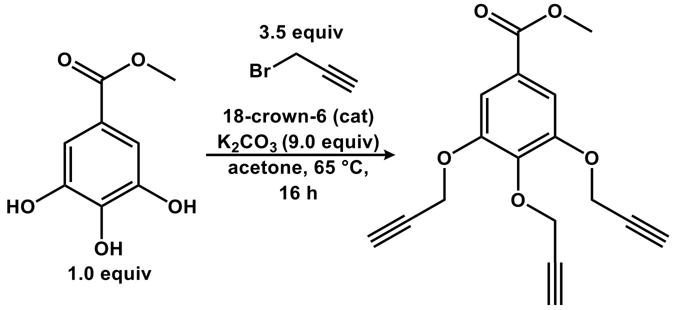

The synthesis of methyl 1,2,3-tris(propynyloxy)benzoate was adapted from the literature,[11] and spectroscopic characterization of the product match the reported data.[12]

To a solution of methyl gallate (5.000 g, 27.15 mmol, 1.000 equiv), K_2CO_3 (33.774 g, 244.44 mmol, 9.000 equiv) and 18-crown-6 (358 mg, 1.36 mmol, 0.05 equiv) in acetone (100 mL) was added a solution of propargyl bromide (10.3 mL of a 9.2 M solution, 95.1 mmol, 3.50 equiv), and the suspension was heated at reflux for 16 h. The reaction mixture was then allowed to cool to ambient temperature and diluted with EtOAc (250 mL). The mixture was washed with a saturated aqueous solution of NaCl (3 x 150 mL), and the organic phase was dried over $MgSO_4$ and filtered through a pad of Celite. The filtrate was evaporated to dryness to yield methyl 1,2,3-tris(propynyloxy)benzoate as a colorless powder, which was used in the next step without further purification (8.036 g, 27.00 mmol, 99%). M.p. 82–85 °C. ^{1}H NMR (300 MHz, 25 °C, $CDCl_3$) δ: 7.47 (s, 2H, *o*-Ar-*H*), 4.83 (d, 2H, *J* = 2.4 Hz, *p*-C*H*$_2$), 4.80 (d, 4H, *J* = 2.4 Hz, *m*-C*H*$_2$), 3.91 (s, 3H, C*H*$_3$), 2.53 (t, 2H, *J* = 2.4 Hz, *m*-C≡C*H*), 2.46 (t, 1H, *J* = 2.4 Hz, *p*-C≡C*H*) ppm.

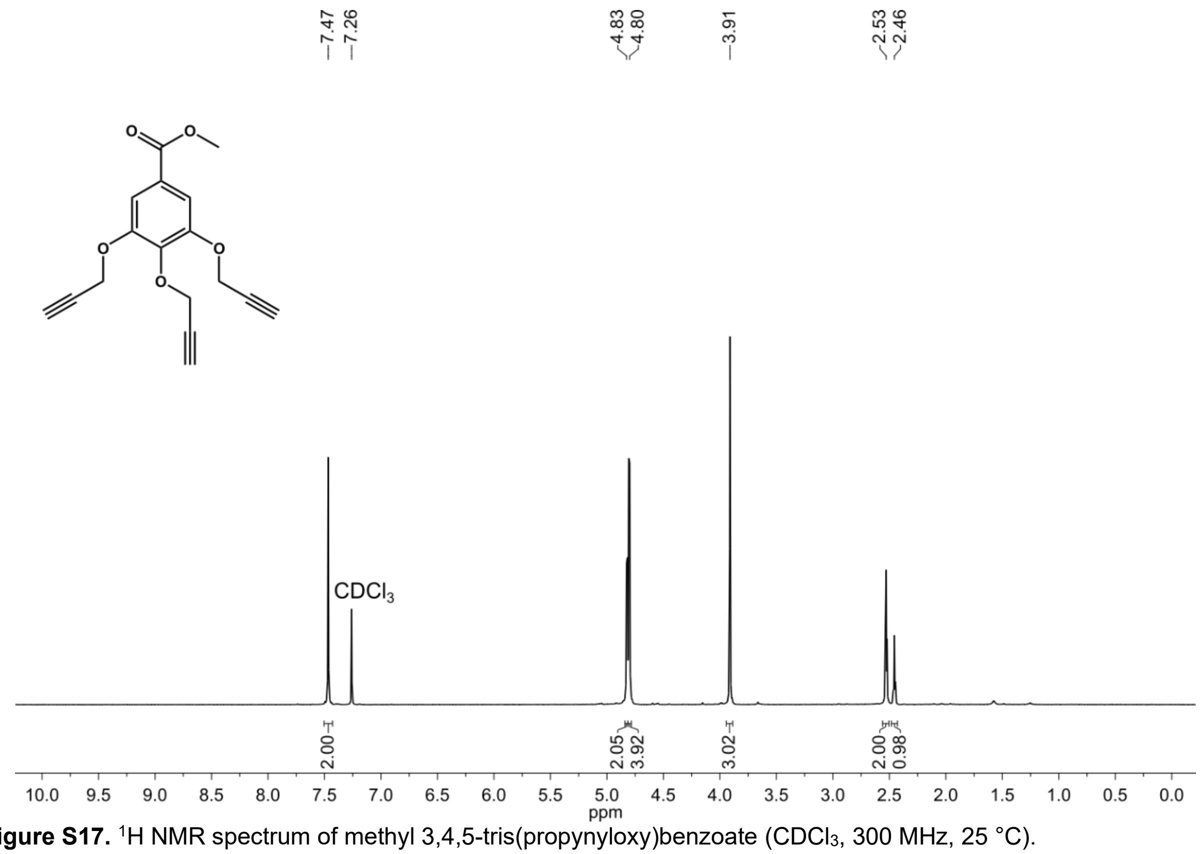

Figure S17. ^{1}H NMR spectrum of methyl 3,4,5-tris(propynyloxy)benzoate ($CDCl_3$, 300 MHz, 25 °C).

S2.1.11. 5-(hydroxymethyl)-1,2,3-tris(propynyloxy)benzene

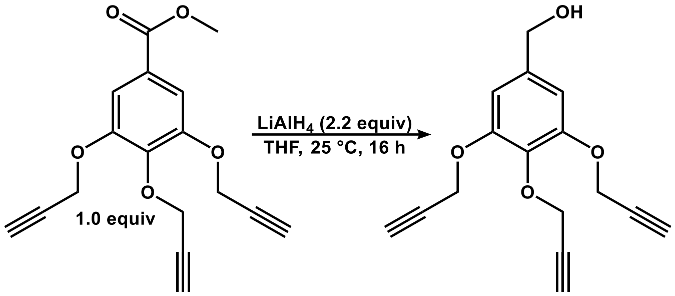

The synthesis of 5-(hydroxymethyl)-1,2,3-tris(propynyloxy)benzene was adapted from the literature,[11] and spectroscopic characterization of the product match the reported data.[12]

In a 200 mL Schlenk flask in the glovebox, a solution of methyl 3,4,5-bis(propynyloxy)benzoate (8.036 g, 26.96 mmol, 1.000 equiv) in THF (30 mL) was added dropwise to a suspension of $LiAlH_4$ (2.200 g, 57.89 mmol, 2.200 equiv) in THF (50 mL) over the course of 20 min at -35 °C. The reaction was allowed to warm to glovebox temperature and stirred for a total of 16 h, at which point the $LiAlH_4$ was quenched with H_2O (1 mL), NaOH (1 mL of a 15 wt% solution), and H_2O (10 mL) sequentially. To the mixture was added EtOAc (200 mL), and the organic phase was washed with a saturated aqueous solution of NaCl (3 x 150 mL). The organic phase was dried over $MgSO_4$, filtered through a pad of Celite and evaporated to dryness to yield 5-(hydroxymethyl)-1,2,3-tris(propynyloxy)benzene as a colorless solid, which was used in the next step without further purification (6.100 g, 22.22 mmol, 84%). M.p. 103–106 °C. ^{1}H NMR (300 MHz, 25 °C, $CDCl_3$) δ: 6.78 (s, 2H, *o*-Ar-*H*), 4.77 (d, 4H, *J* = 2.4 Hz, *m*-C*H*$_2$), 4.73 (d, 2H, *J* = 2.4 Hz, *p*-C*H*$_2$), 4.65 (d, 2H, C*H*$_2$OH), 2.51 (t, 2H, *J* = 2.4 Hz, *m*-C≡C*H*), 2.45 (t, 1H, *J* = 2.4 Hz, *p*-C≡C*H*), 1.71 (t, 1H, O*H*) ppm.

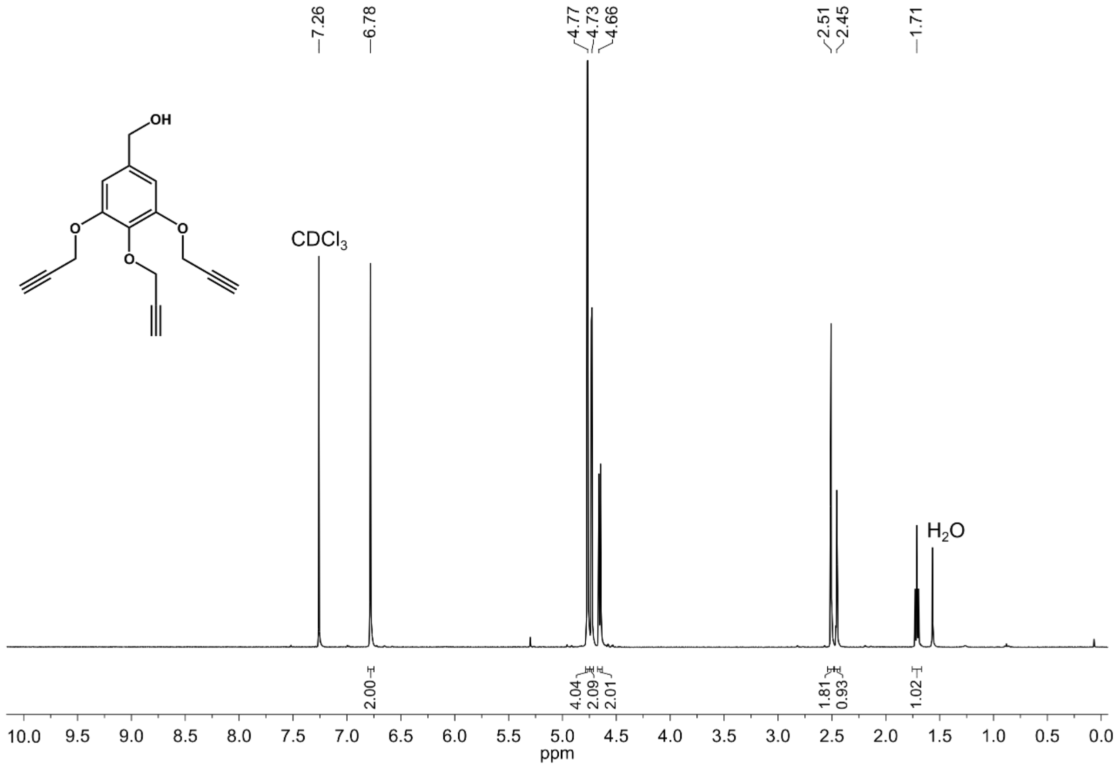

Figure S18. ^{1}H NMR spectrum of 5-(hydroxymethyl)-1,2,3-tris(propynyloxy)benzene ($CDCl_3$, 400 MHz, 25 °C).

S2.1.12. 5-(bromomethyl)-1,2,3-tris(propynyloxy)benzene

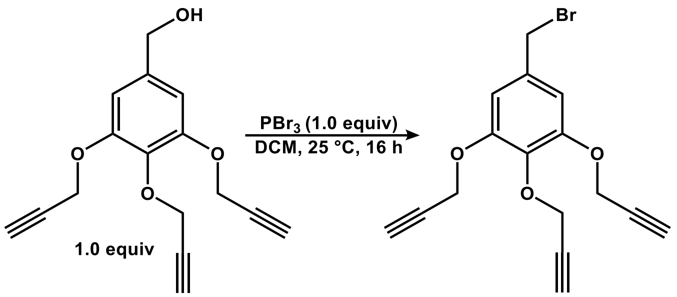

The synthesis of 5-(bromomethyl)-1,2,3-tris(propynyloxy)benzene was adapted from the literature,[11] and spectroscopic characterization agrees with the reported data.[12]

In a 200 mL two-neck flask under an atmosphere of N_2, PBr_3 (2.1 mL, 22 mmol, 1.0 equiv) was added to a solution of 5-(hydroxymethyl)-1,2,3-tris(propynyloxy)benzene (6.100 g, 22.22 mmol, 1.000 g) in DCM (100 mL) at 0 °C dropwise over the course of 20 min. The reaction mixture was allowed to warm to ambient temperature, and then stirred for an additional 16 h. The mixture was diluted with DCM (150 mL) and washed with a saturated aqueous solution of $NaHCO_3$ (2 x 150 mL), followed by a saturated aqueous solution of NaCl (3 x 150 mL). The organic phase was dried over $MgSO_4$, filtered through a pad of Celite and evaporated to dryness. The product was redissolved in a minimal amount of DCM and precipitated with hexanes to yield 5-(bromomethyl)-1,2,3-tris(propynyloxy)benzene as a colorless solid that was used in the next step without further purification (5.300 g, 15.92 mmol, 72%). M.p. 63–66 °C. ^{1}H NMR (300 MHz, 25 °C, $CDCl_3$) δ: 6.80 (s, 2H, *o*-Ar-*H*), 4.77 (d, 4H, *J* = 2.5 Hz, *m*-C*H*$_2$), 4.73 (d, 2H, *J* = 2.5 Hz, *p*-C*H*$_2$), 4.46 (s, 2H, C*H*$_2$Br), 2.53 (t, 2H, *J* = 2.5 Hz, *m*-C≡*H*), 2.47 (t, 2H, *J* = 2.5 Hz, *p*-C≡*H*) ppm.

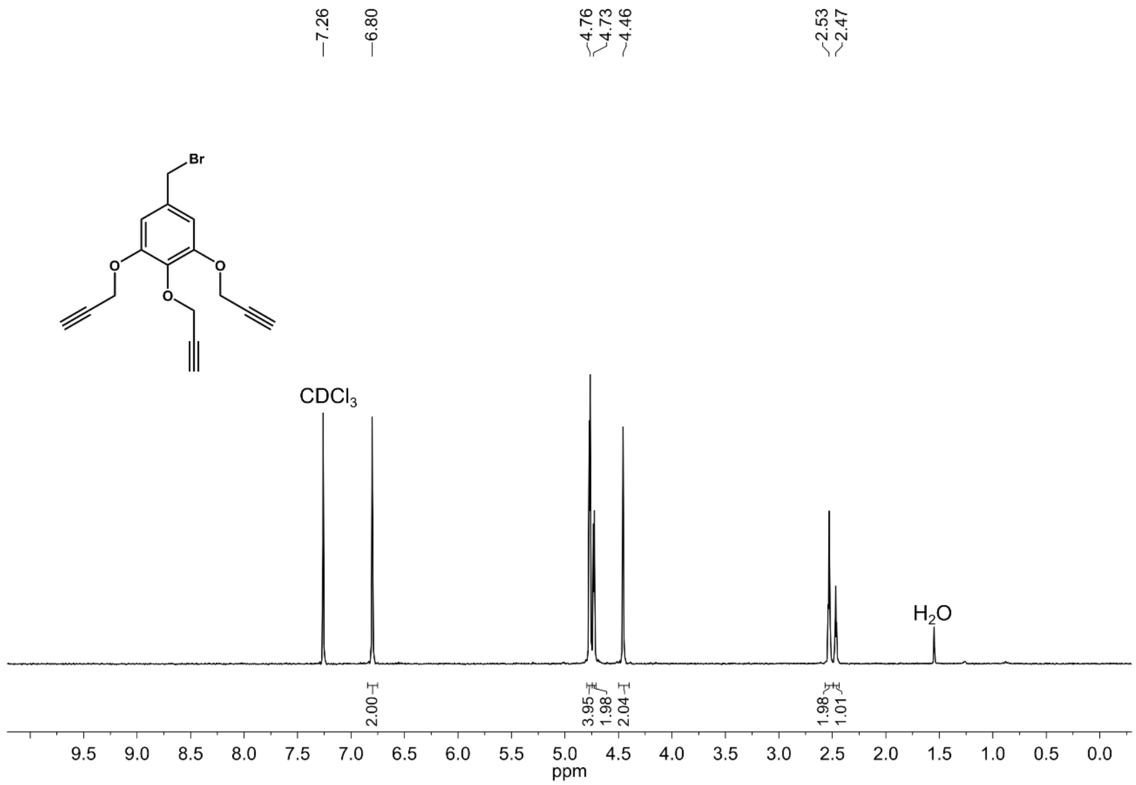

Figure S19. ^{1}H NMR spectrum of 5-(bromomethyl)-1,2,3-tris(propynyloxy)benzene ($CDCl_3$, 300 MHz, 25 °C).

S2.1.13. 5-((1,2,3-tris(propynyloxy)benzyl)oxy)picolinaldehyde (b)

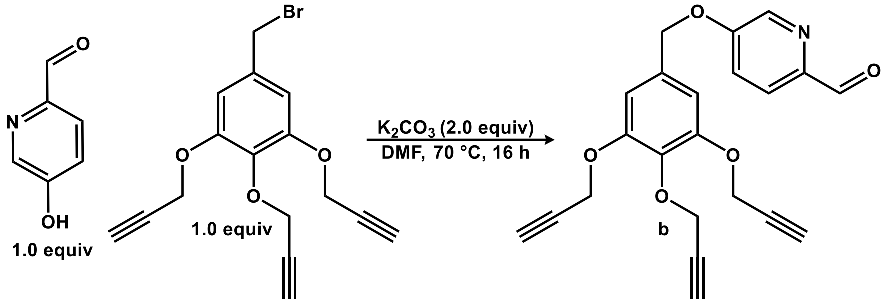

In a 50 mL Schlenk flask under an atmosphere of N_2, a DMF (10 mL) solution of 5-(bromomethyl)-1,2,3-tris(propynyloxy)benzene (600 mg, 1.81 mmol, 1.00 equiv), 5-hydroxypicolinaldehyde (222 mg, 1.81 mmol, 1.00 equiv), and K_2CO_3 (500 mg, 3.62 mmol, 2.00 equiv) was heated at 70 °C for 16 h. The reaction mixture was allowed to cool to ambient temperature, diluted with EtOAc (150 mL) and washed with a saturated aqueous solution of NaCl (3 x 150 mL). The organic phase was dried over $MgSO_4$, filtered through a pad of Celite and evaporated to dryness to yield a brown oil, which was purified by flash liquid chromatography on silica gel eluting with a solvent gradient of 30:70 to 60:40 EtOAc/hexanes to yield 5-((1,2,3-tris(propynyloxy)benzyl)oxy)picolinaldehyde (b) as a colorless powder (536 mg, 1.43 mmol, 79%). M.p. 97–100 °C. ^{1}H NMR (400 MHz, 25 °C, $CDCl_3$) δ: 9.97 (s, 1H, OC*H*), 8.49 (d, 1H, ^4J = 2.6 Hz, py-*H*-6), 7.93 (d, 1H, ^3J = 8.7 Hz, py-*H*-3), 7.35 (dd, 1H, ^3J = 8.7 Hz, ^4J = 2.8 Hz, py-*H*-4), 6.83 (s, 2H, *o*-Ar-*H*), 5.14 (s, 2H, C*H*$_2$-O-py), 4.76 (d, 4H, *J* = 2.5 Hz, *m*-C*H*$_2$), 4.72 (d, 2H, *J* = 2.5 Hz, *p*-C*H*$_2$), 2.49 (t, 2H, *J* = 2.5 Hz, *m*-C≡C*H*), 2.46 (t, 1H, *J* = 2.5 Hz, *p*-C≡C*H*) ppm. ^{13}C{^{1}H} NMR (100 MHz, 25 °C, $CDCl_3$) δ: 192.1 (O*C*H), 158.0 (py-*C*-2), 152.0 (*m*-Ar-*C*), 146.6 (py-*C*-5), 139.2 (py-*C*-6), 137.4 (*p*-Ar-*C*), 131.4 (Ar-*C*), 123.4 (py-*C*-3), 121.2 (py-*C*-4), 108.0 (*o*-Ar-*C*), 79.1 (*p*-*C*≡CH), 78.3 (*m*-*C*≡CH), 76.2 (*m*-C≡*C*H), 75.5 (*p*-C≡*C*H), 70.6 (*C*H$_2$-O-py), 60.5 (*p*-ArO-*C*H$_2$), 57.2 (*m*-ArO-*C*H$_2$) ppm. ATR-IR (ν): 3270, 2115 (C≡C), 1696 (HC=O), 1558, 1446, 1316, 1264, 1208, 1107, 984, 842, 768, 678, 634, 544 cm^{-1}.

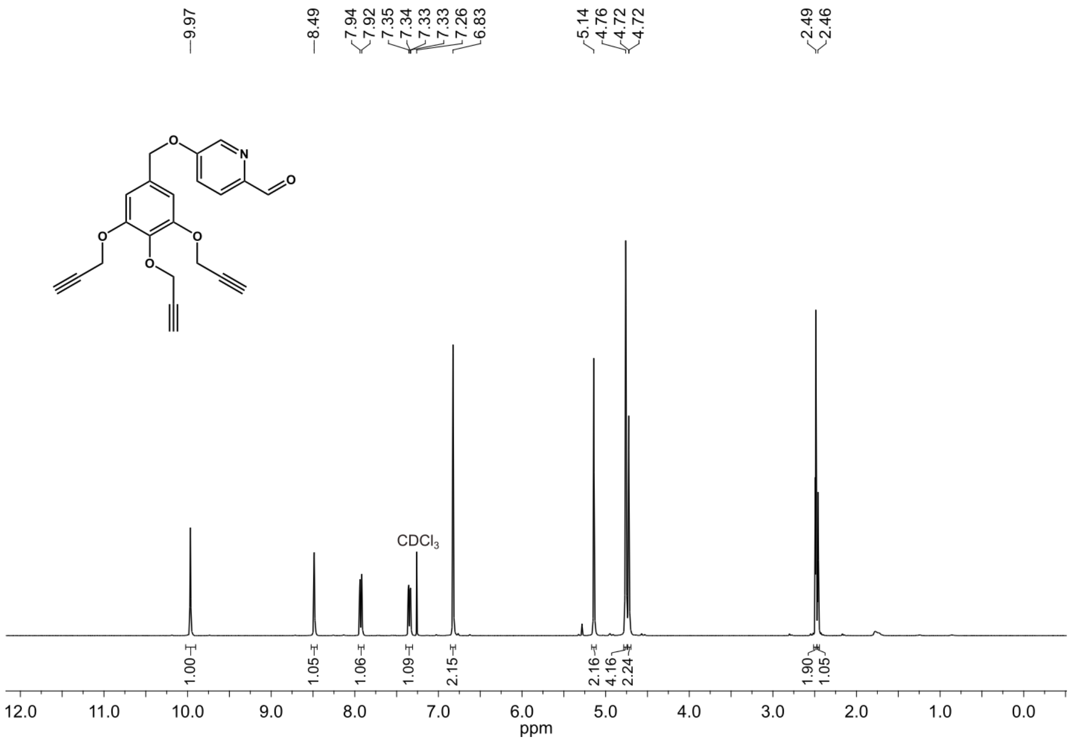

Figure S20. ^{1}H NMR spectrum of 5-((1,2,3-tris(propynyloxy)benzyl)oxy)picolinaldehyde ($CDCl_3$, 400 MHz, 25 °C).

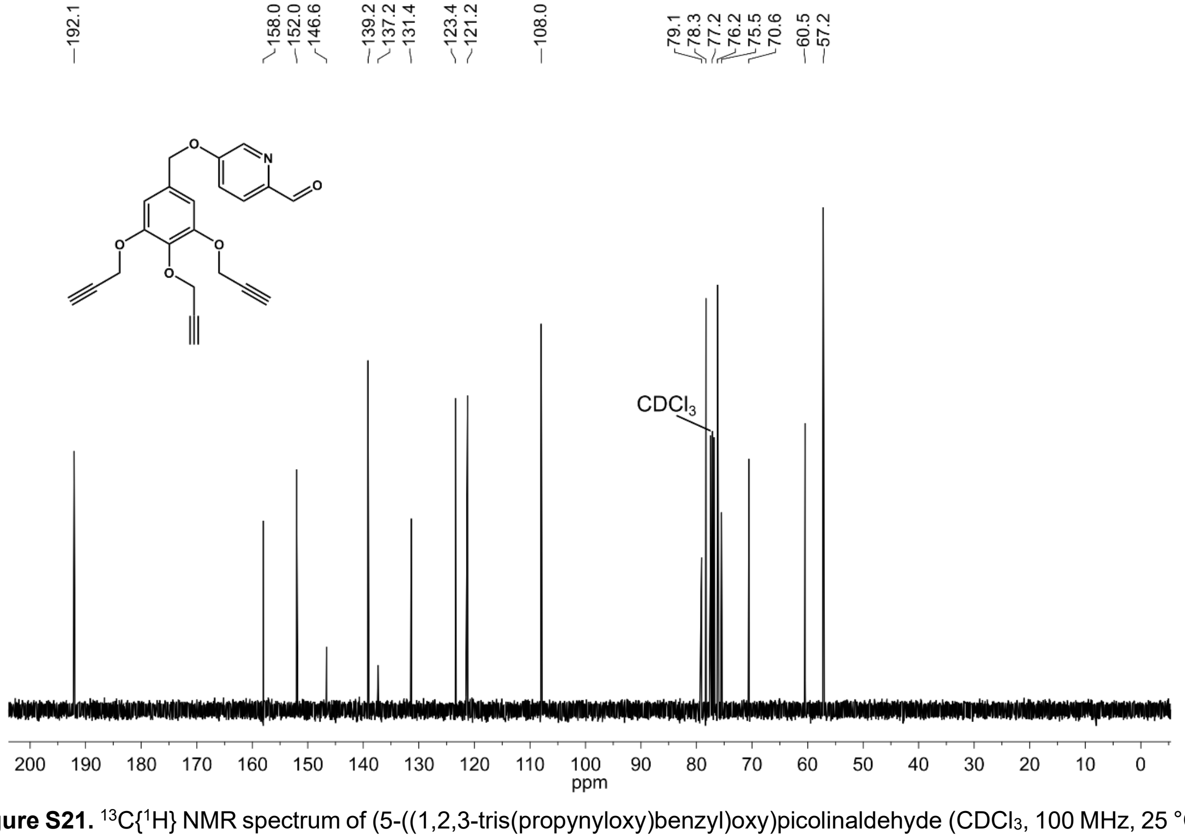

Figure S21. $^{13}C\{^1H\}$ NMR spectrum of (5-((1,2,3-tris(propynyloxy)benzyl)oxy)picolinaldehyde ($CDCl_3$, 100 MHz, 25 °C).

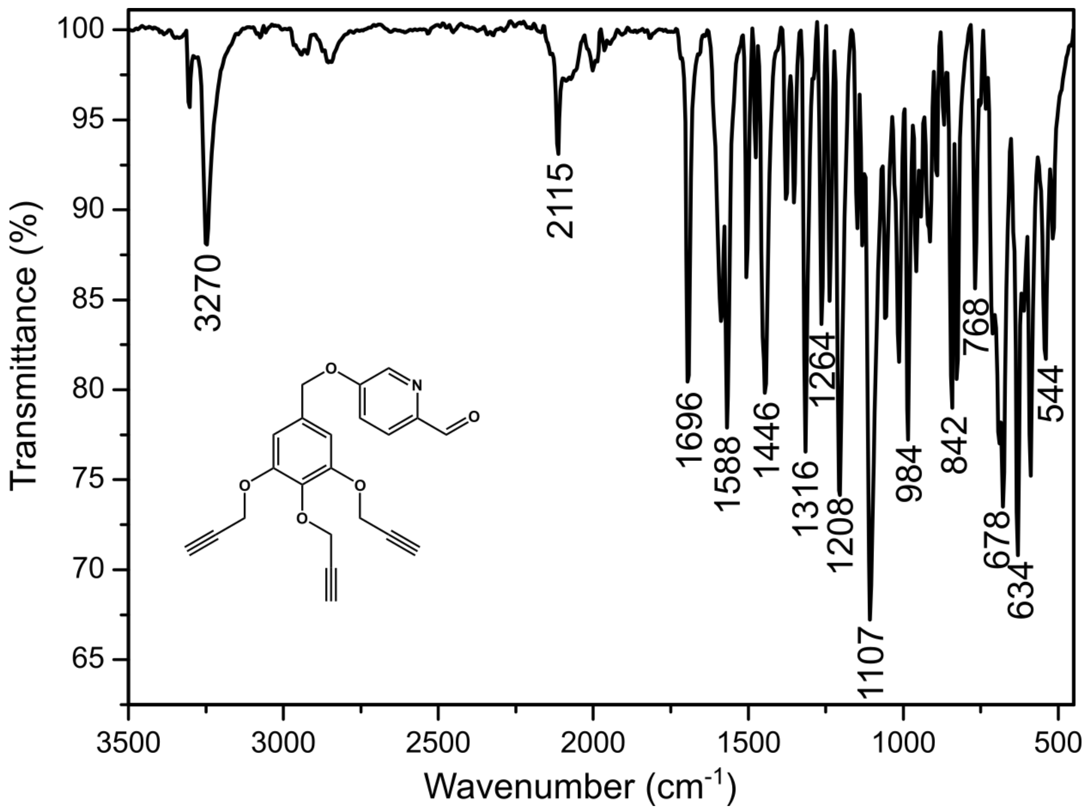

Figure S22. ATR-IR spectrum of (5-((1,2,3-tris(propynyloxy)benzyl)oxy)picolinaldehyde.

S2.1.14. Man-3(OAc)

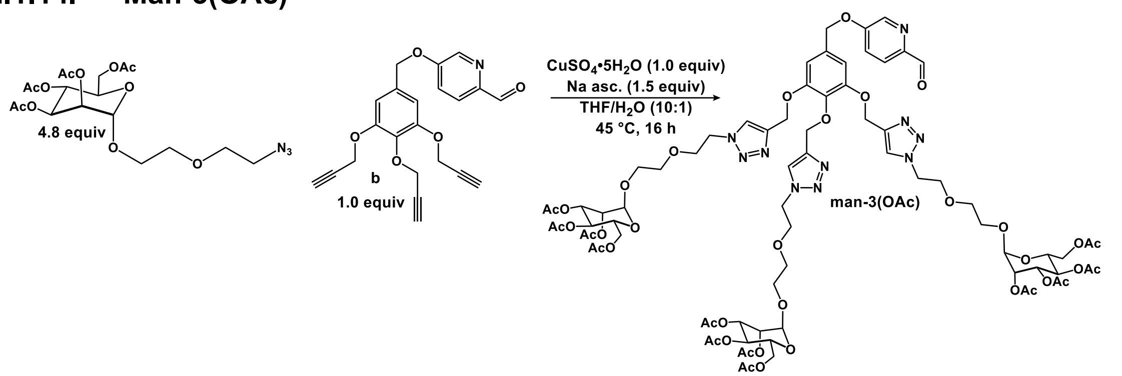

To a solution of $CuSO_4$•$5H_2O$ (100 mg, 0.400 mmol, 1.00 equiv) in H_2O (1 mL) in a 25 mL Schlenk flask was added a solution of 5-((1,2,3-tris(propynyloxy)benzyl)oxy)picolinaldehyde (b) (150 mg, 0.400 mmol, 1.00 equiv), sodium ascorbate (119 mg, 0.600 mmol, 1.50 equiv), and 2-(2-azidoethoxy)ethyl(OAc)$_4$-α-D-mannose (882 mg, 1.92 mmol, 4.80 equiv) in THF (10 mL). The yellow heterogeneous reaction mixture was allowed to stir under an atmosphere of N_2 for 16 h, at which point it was allowed to cool to ambient temperature. The mixture was diluted with EtOAc (150 mL) and washed with an aqueous solution of Na_4[EDTA] (3 x 30 mL), followed by a saturated aqueous solution of NaCl (3 x 150 mL). The organic phase was dried over $MgSO_4$, filtered through a pad of Celite and evaporated to dryness to yield a yellow oil that was first purified on silica gel eluting with a 60:40 EtOAc/hexanes mixture (100 mL) to remove unreacted 2-(2-azidoethoxy)ethyl(OAc)$_4$-α-D-mannose followed by acetone (50 mL). The acetone fraction was evaporated, and the residue was purified by flash liquid chromatography eluting with a solvent system of 0:100 to 5:95 MeOH/DCM to yield man-3(OAc) as a colorless powder (404 mg, 0.230 mmol, 57%). M.p. 52–55 °C. ^{1}H NMR (400 MHz, 25 °C, $CDCl_3$) δ: 9.91 (s, 1H, OC*H*), 8.42 (d, 1H, ^4J = 2.3 Hz, py-*H*-6), 7.89 (d, 1H, ^3J = 8.6 Hz, py-*H*-3), 7.84 (s, 3H, N-C*H*), 7.31 (dd, 1H, ^3J = 8.6 Hz, ^4J = 2.3 Hz, py-*H*-4), 6.80 (s, 2H, *o*-Ar-*H*), 5.24 (dd, 3H, *J* = 10.1, 3.3 Hz, man-*H*-3), 5.21 (d, 3H, *J* = 10.1 Hz, man-*H*-2), 5.18 (dd, 3H, *J* = 3.1, 1.8 Hz, man-*H*-4), 5.16 (s, 4H, *m*-CH_2), 5.11 (s, 2H, CH_2-Opy), 5.05 (s, 2H, *p*-CH_2), 4.78 (d, 3H, *J* = 1.1 Hz, man-*H*-1), 4.51 (t, 4H, *J* = 5.1, OCH_2), 4.46 (t, 2H, *J* = 5.1 Hz, OCH_2), 4.21–4.17 (m, 3H, man-*H*-5), 4.03 (t, 2H, *J* = 2.8 Hz, man-*H*-6_a), 4.00 (t, 1H, *J* = 2.8 Hz, man-*H*-$6_{a'}$), 4.95-3.91 (m, 3H, man-*H*-6_b), 3.87-3.80 (m, 6H, OCH_2), 3.74-3.67 (m, 3H, OCH_2), 3.57-3.54 (m, 9H, OCH_2), 2.07 (s, 3H, CH_3COO), 2.07 (s, 6H, CH_3COO), 2.01 (s, 9H, CH_3COO, two signals overlapping), 1.96 (s, 9H, CH_3COO two signals overlapping), 1.91 (s, 3H, CH_3COO), 1.90 (s, 6H, CH_3COO) ppm. ^{13}C{^{1}H} NMR (100 MHz, 25 °C, $CDCl_3$) δ: 191.9 (O*C*H), 170.6 (CH_3*C*OO, two signals overlapping), 170.02 (CH_3*C*OO), 169.99 (CH_3*C*OO), 169.90 (CH_3*C*OO), 169.87 (CH_3*C*OO), 169.69 (CH_3*C*OO), 169.67 (CH_3*C*OO), 157.9 (py-*C*-2), 152.5 (*m*-Ar-*C*), 146.4 (py-*C*-5), 144.4 (*p*-C=*C*N), 143.5 (*m*-C=*C*N), 139.0 (py-*C*-6), 137.7 (*p*-Ar-*C*), 131.0 (Ar-*C*), 124.6 (*p*-N-*C*=C), 124.3 (*m*-N-*C*=C), 123.4 (py-*C*-3), 121.1 (py-*C*-4), 107.3 (*o*-Ar-*C*), 97.6 (man-*C*-1, two signals overlapping), 70.6 (*C*H_2-O-py), 69.9 (O*C*H_2, two signals overlapping), 69.9 (O*C*H_2, two signals overlapping), 69.4 (man-*C*-2, two signals overlapping), 69.0 (man-*C*-3, two signals overlapping), 68.5 (man-*C*-5), 68.4 (man-*C*-5), 67.1 (man-*C*-4, two signals overlapping), 66.4 (O*C*H_2, two signals overlapping), 66.0 (O*C*H_2, two signals overlapping), 62.9 (man-*C*-6, two signals overlapping), 62.4 (man-*C*-6, two signals overlapping), 50.2 (*m*-ArO*C*H_2), 49.9 (*p*-ArO*C*H_2), 20.8 (CH_3*C*OO, two signals overlapping), 20.70 (CH_3*C*OO, two signals overlapping), 20.65 (CH_3*C*OO, two signals overlapping), 20.63 (CH_3*C*OO), 20.61 (CH_3*C*OO) ppm *(two decimal places are given only where needed to distinguish resonances)*. HR-ESI-MS(+) (*m/z*): 1781.5934 ($[M+Na]^+$, calc'd, 1781.5941). ATR-IR (ν): 2937, 1741 (HC=O), 1573, 1438, 1372, 1219, 1044, 977, 596 cm^{-1}.

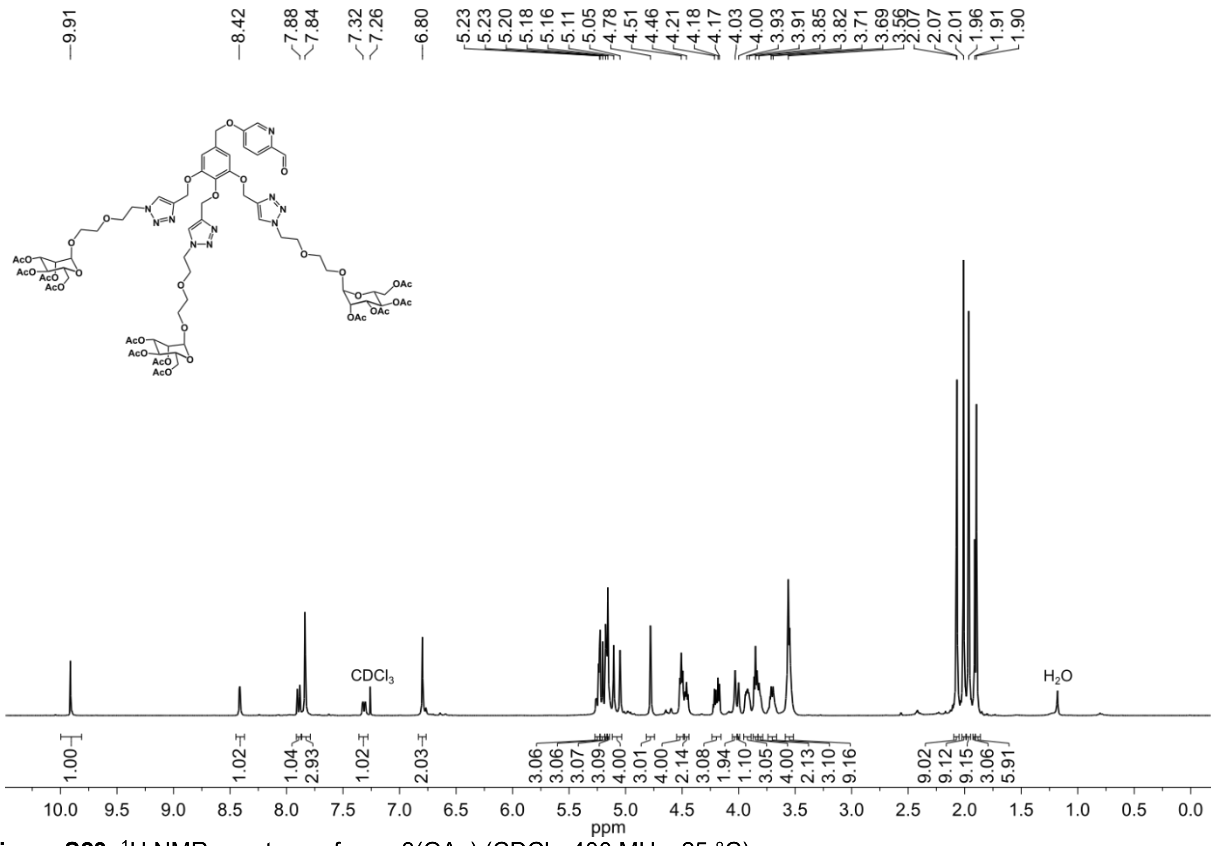

Figure S23. ^{1}H NMR spectrum of man-3(OAc) ($CDCl_3$, 400 MHz, 25 °C).

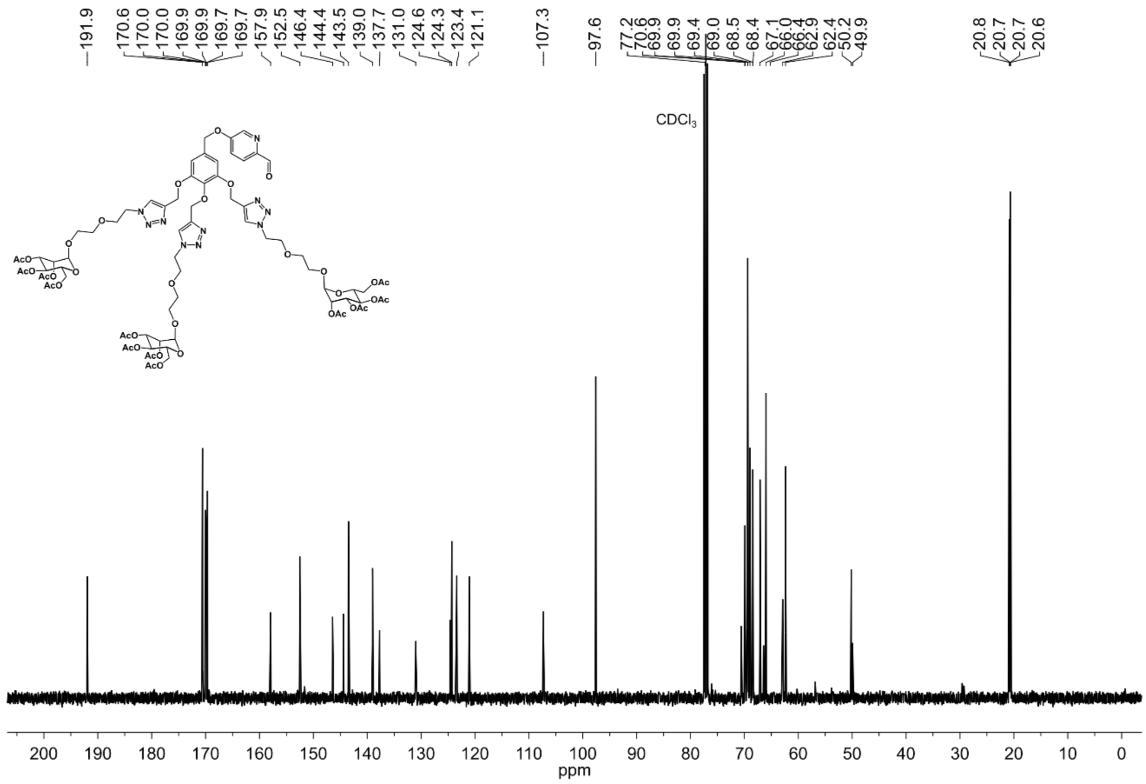

Figure S24. $^{13}C\{^{1}H\}$ NMR spectrum of man-3(OAc) ($CDCl_3$, 100 MHz, 25 °C).

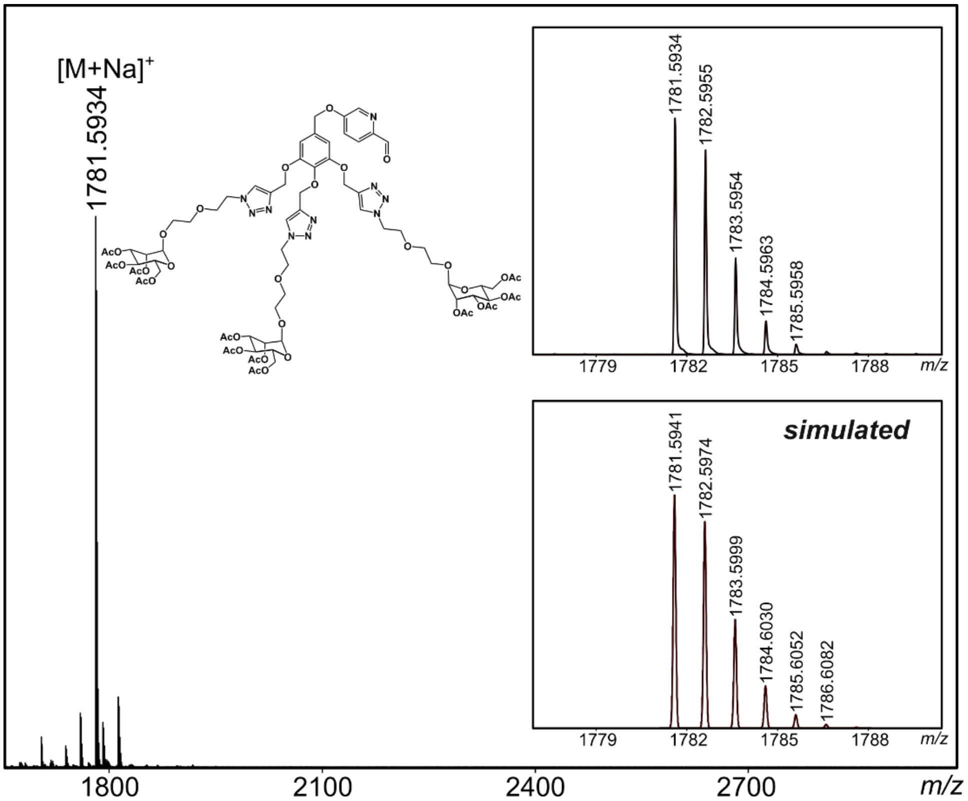

Figure S25. HR-ESI-MS(+) of man-3(OAc) (measured in CH_3OH).

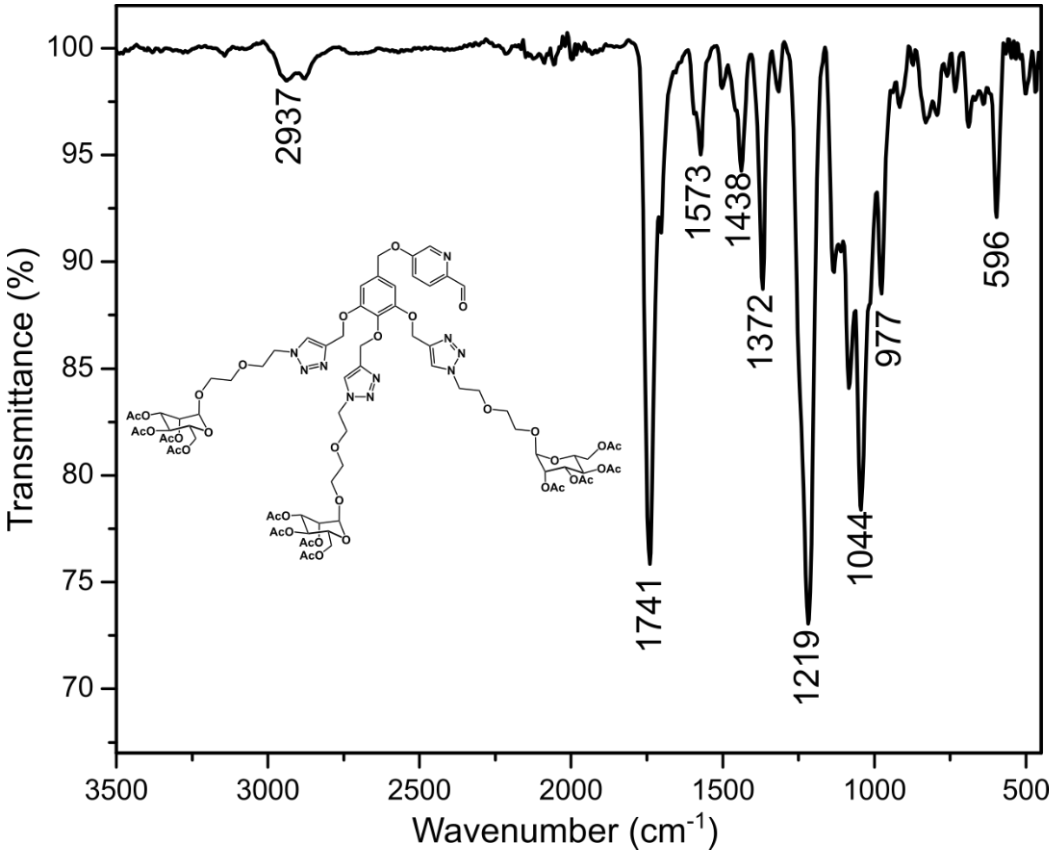

Figure S26. ATR-IR spectrum of man-3(OAc).

S2.1.15. Man-3

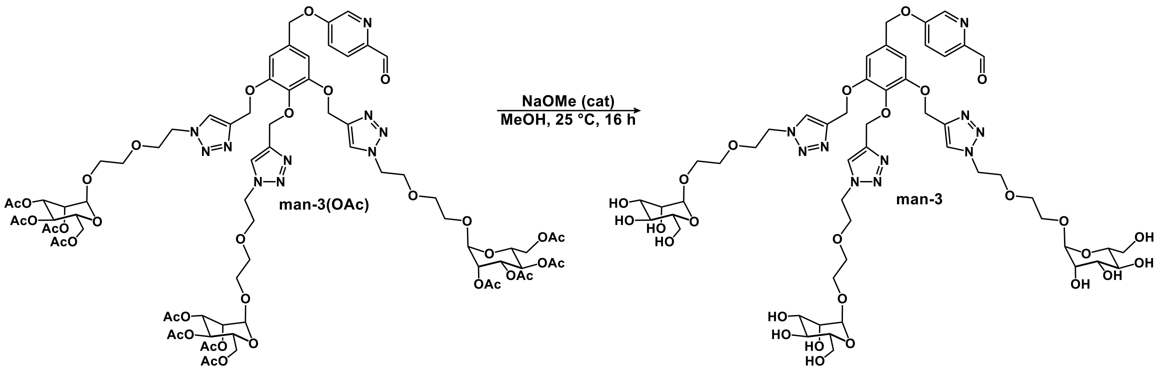

To a stirring solution of man-3(OAc) (85 mg, 0.047 mmol, 1.0 equiv) in MeOH (10 mL) was added a solution of NaOMe in MeOH until the pH reached 8 (400 µL of a 0.3 M solution, 0.12 mmol, 2.6 equiv). The reaction mixture was allowed to stir at ambient temperature under an atmosphere of N_2 for 16 h, at which point it was neutralized with Amberlyst®-15 (180 mg). The solution was filtered through a pad of Celite, and all volatiles were removed under reduced pressure to afford man-3 as a colorless solid that was used without further purification (yield: 41 mg, 0.033 mmol, 67%). ^{1}H NMR (400 MHz, 25 °C, D_2O) δ: 9.79 (s, 1H, OC*H*), 8.36 (s, 1H, py-*H*-6), 8.03 (s, 2H, N-C*H*), 7.92 (d, 1H, ^3J = 8.3 Hz, py-*H*-3), 7.79 (s, 1H, N-C*H*), 7.45 (s, 1H, py-*H*-4), 6.85 (s, 2H, *o*-Ar), 5.14 (s, 2H, man-*H*-1), 5.07 (s, 1H, man-*H*-1), 4.73 (s, 4H, –CH_2–), 4.57 (s, 4H, *m*-CH_2), 4.48 (s, 2H, *p*-CH_2), 3.91-3.79 (m, 12H, man-*H*-2, man-*H*-6$_b$, man-*H*-6$_a$, man-*H*-3 signals overlapping), 3.73–3.68 (m, 10H, man-*H*-4, man-*H*-5, –CH_2– signals overlapping), 3.62 (t, 4H, –CH_2–), 3.53-3.46 (m, 14H, –CH_2–) ppm. ESI-MS(+) (*m/z*): 628.30 ([M+2H]$^{2+}$, calc'd, 628.25).

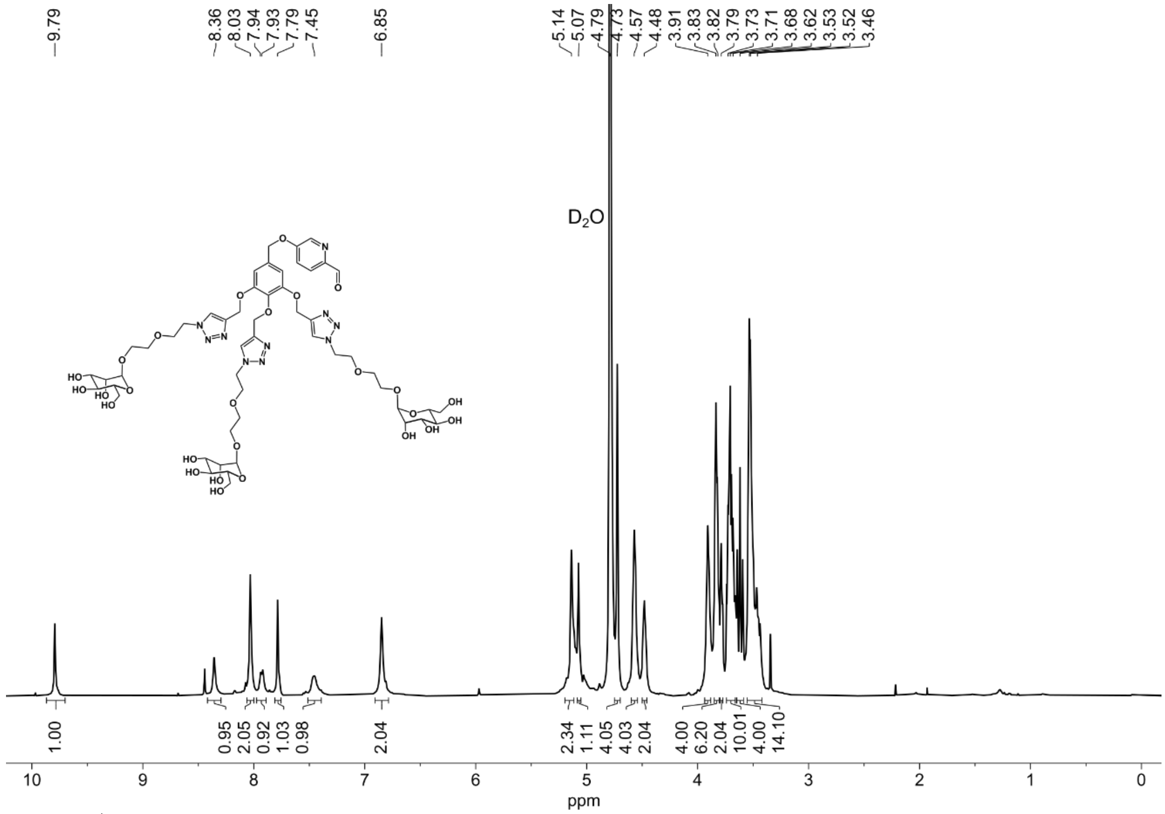

Figure S27. ^{1}H NMR spectrum of man-3 (D_2O, 400 MHz, 25 °C).

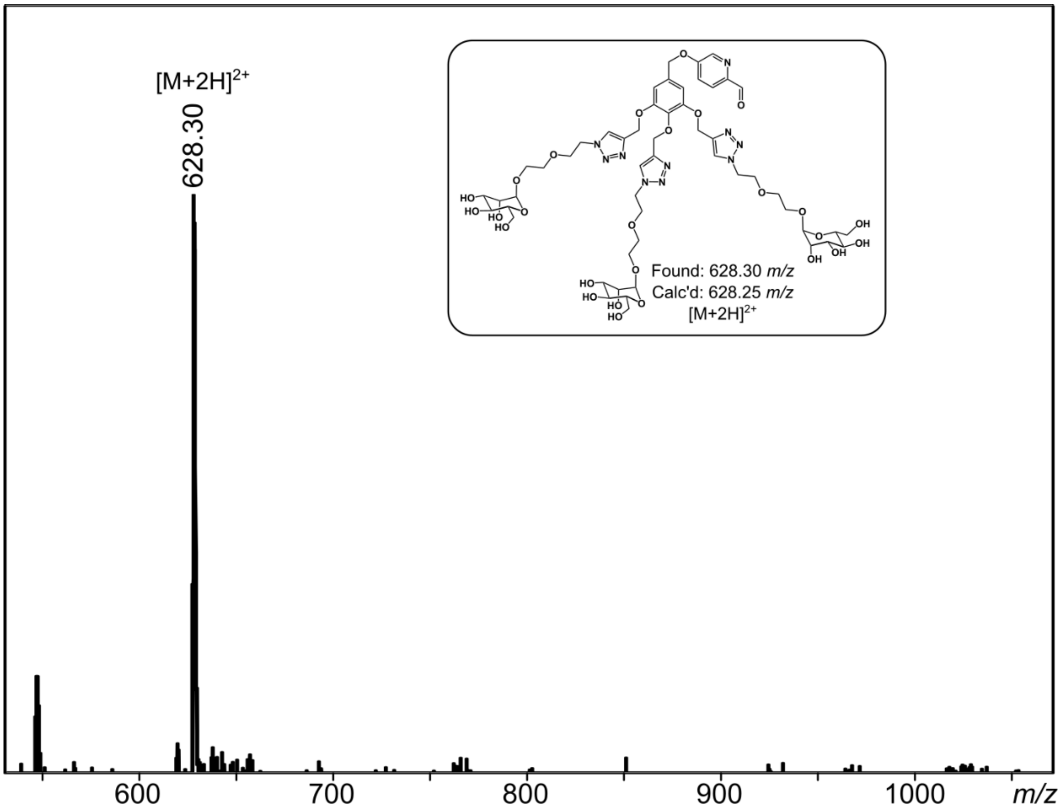

Figure S28. ESI-MS(+) of man-3.

S2.1.16. (3,5-bis((3,4,5-tris(propynyloxy)benzyl)oxy)phenyl)methanol

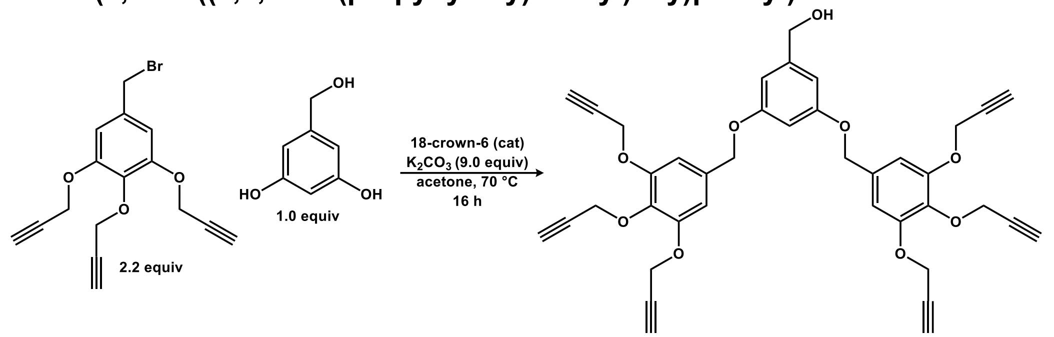

The synthesis of (3,5-bis((3,4,5-tris(propynyloxy)benzyl)oxy)phenyl)methanol was adapted from the literature,[11] and spectroscopic characterization of the product match the reported data.[12]

A 200 mL round bottom flask was charged with a solution of 3,5-dihydroxybenzyl alcohol (191 mg, 1.36 mmol, 1.00 equiv), 5-(bromomethyl)-1,2,3-tris(propynyloxy) (1.000 g, 3.000 mmol, 2.200 equiv), K_2CO_3 (1.695 g, 12.29 mmol, 9.000 equiv), and 18-crown-6 (72 mg, 0.27 mmol, 0.20 equiv) in acetone (500 mL), and the suspension was heated at reflux for 16 h. The reaction mixture was then allowed to cool to ambient temperature and diluted with EtOAc (200 mL). The mixture was washed with a saturated aqueous solution of NaCl (3 x 150 mL), and the organic phase was dried over $MgSO_4$ and filtered through a pad of Celite. The filtrate was evaporated to dryness and the product was purified by flash liquid chromatography on silica gel, eluting with a solvent-system of 30:70 to 60:10 EtOAc/hexanes to yield (3,5-bis((3,4,5-tris(propynyloxy)benzyl)oxy)phenyl)methanol as a colorless powder (748 mg, 1.16 mmol, 85%). M.p. 107–109 °C. ^{1}H NMR (400 MHz, 25 °C, $CDCl_3$) δ: 6.83 (s, 4H, *o*-Ar-*H*), 6.62 (d, 2H, ^4J = 2.0 Hz, *o*-Ar-*H*), 6.53 (t, 1H, ^4J = 2.0 Hz, *p*-Ar-H), 4.99 (s, 4H, ArCH_2O), 4.76 (d, 8H, ^4J = 2.4 Hz, *m*-CH_2C≡C), 4.73 (d, 4H, ^4J = 2.4 Hz, *p*-CH_2C≡C), 4.65 (s, 2H, CH_2OH), 2.49 (t, 4H, ^4J = 2.4 Hz, *m*-C≡C*H*), 2.46 (t, 2H, ^4J = 2.4 Hz, *p*-C≡C*H*), 1.63 (s, 1H, O*H*) ppm. ^{13}C{^{1}H} NMR (100 MHz, 25 °C, $CDCl_3$) δ: 160.0 (*m*-Ar-C_d), 151.8 (*m*-Ar-C_i), 143.6 (Ar-C_b), 136.9 (*p*-Ar-C_j), 133.1 (Ar-C_g), 108.0 (*o*-Ar-C_h), 106.0 (*o*-Ar-C_c), 101.7 (*p*-Ar-C_e), 79.2 (*p*-$C_{l'}$≡CH), 78.5 (*m*-C_l≡CH), 76.2 (*m*-C≡C_mH), 75.4 (*m*-C≡$C_{m'}$H), 70.0 (Ar-$C_f$$H_2$-O), 65.3 ($C_a$$H_2$-OH), 60.5 (≡C-$C_{k'}$$H_2$), 57.2 (≡C-$C_k$$H_2$) ppm.

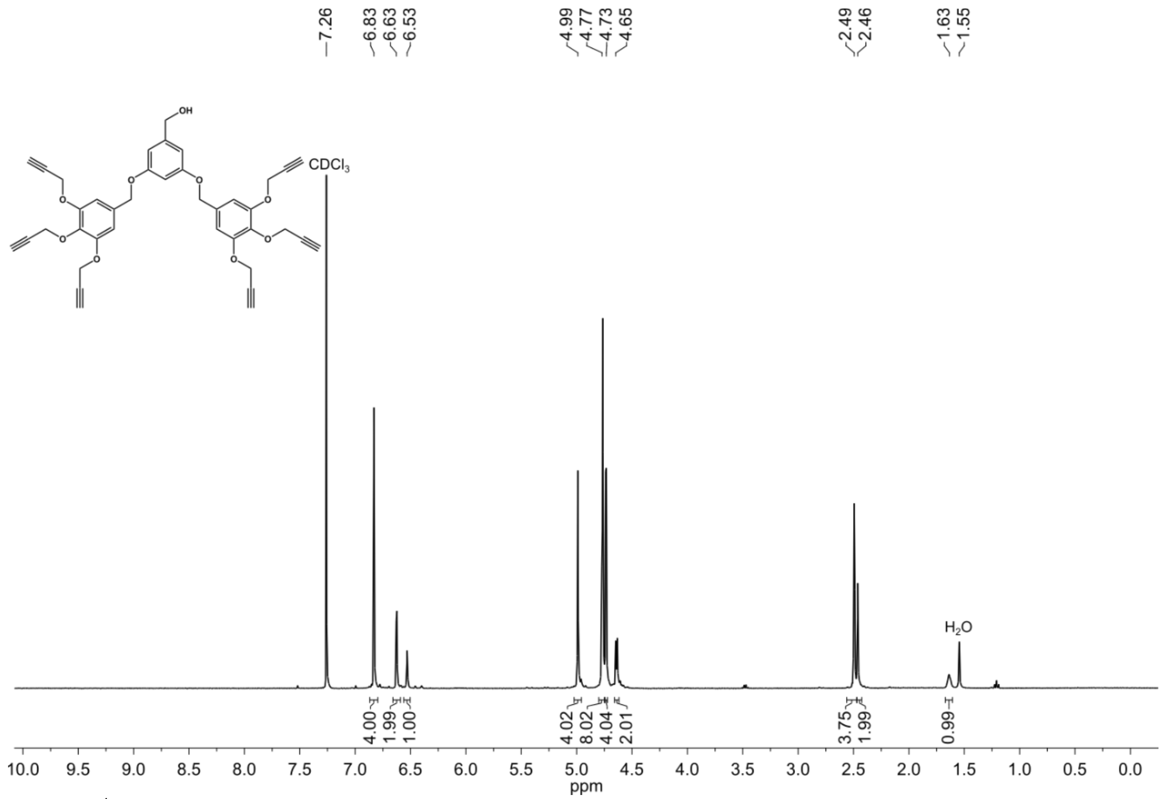

Figure S29. ^{1}H NMR spectrum of (3,5-bis((3,4,5-tris(propynyloxy)benzyl)oxy)phenyl)methanol ($CDCl_3$, 400 MHz, 25 °C).

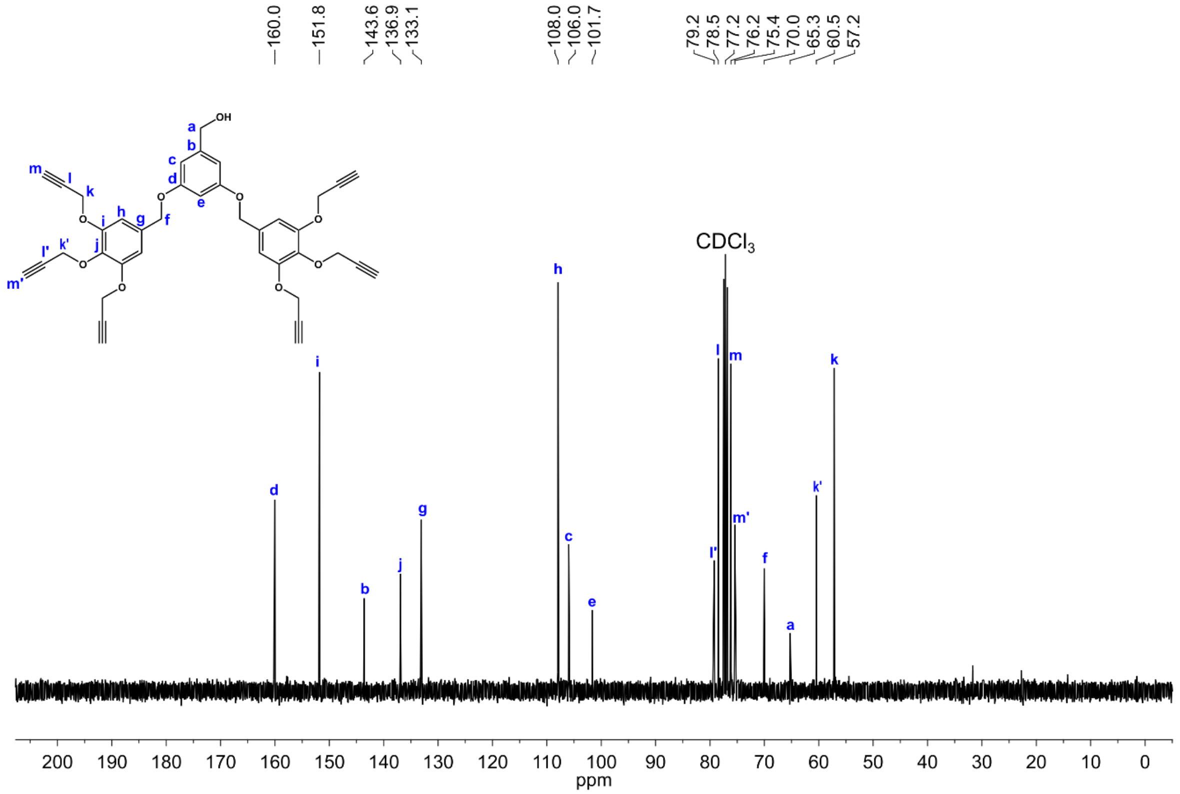

Figure S30. ^{13}C{^{1}H} NMR spectrum of (3,5-bis((3,4,5-tris(propynyloxy)benzyl)oxy)phenyl)methanol ($CDCl_3$, 100 MHz, 25 °C).

S2.1.17. (5-(bromomethyl)-1,3-phenylene)bis(1,2,3-tris(propynyloxy)benzene)

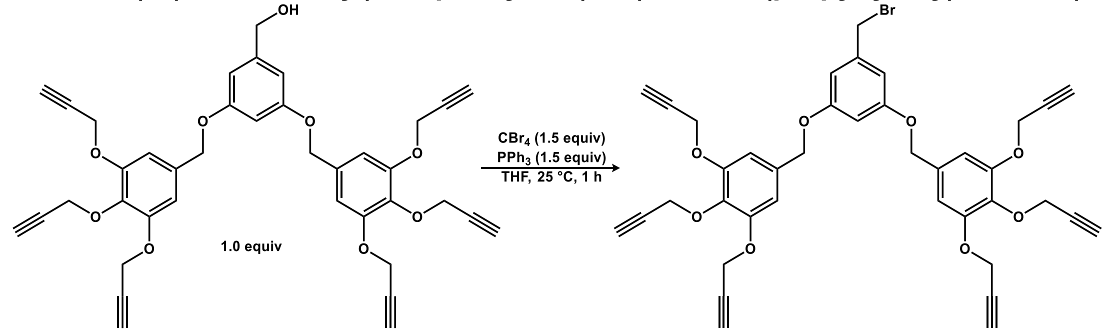

The synthesis of (5-(bromomethyl)-1,3-phenylene)bis(1,2,3-tris(propynyloxy)benzene) was adapted from the literature,[11] and spectroscopic characterization of the product match the reported data.[12]

In the glovebox, to a solution of PPh_3 (1.137 g, 4.335 mmol, 1.500 equiv) and (3,5-bis((3,4,5-tris(propynyloxy)benzyl)oxy)phenyl)methanol (1.862 g, 2.888 mmol, 1.000 equiv) in THF (10 mL) was added CBr_4 (1.438 g, 4.335 mmol, 1.500 equiv) as a solid over the course of 15 min at -35 °C. The solution was allowed to warm to glovebox temperature and stirred for 1 h, during which time solids precipitated out of the solution. The reaction mixture was removed from the glovebox and filtered through a pad of Celite to remove $OPPh_3$, and the filtrate was evaporated under reduced pressure. The crude product was purified by flash liquid chromatography on silica gel eluting with a solvent gradient of 30:70 to 100:0 DCM/hexanes to afford (5-(bromomethyl)-1,3-phenylene)bis(1,2,3-tris(propynyloxy)benzene) as a colorless solid (969 mg, 1.37 mmol, 47%). M.p. 113–116 °C. ^{1}H NMR (400 MHz, 25 °C, $CDCl_3$) δ: 6.83 (s, 4H, *o*-Ar-*H*), 6.63 (d, 2H, ^4J = 2.1 Hz, *o*-Ar-*H*), 6.52 (t, 1H, ^4J = 2.0 Hz, *p*-Ar-H), 4.98 (s, 4H, ArC*H*$_2$O), 4.76 (d, 8H, ^4J = 2.3 Hz, *m*-C*H*$_2$C≡C), 4.73 (d, 4H, ^4J = 2.3 Hz, *p*-C*H*$_2$C≡C), 4.41 (s, 2H, C*H*$_2$Br), 2.49 (t, 4H, ^4J = 2.3 Hz, *m*-C≡C*H*), 2.46 ppm (t, 2H, ^4J = 2.3 Hz, *p*-C≡C*H*) ppm. ^{13}C{^{1}H} NMR (100 MHz, 25 °C, $CDCl_3$) δ: 160.0 (*m*-Ar-C_d), 151.9 (*m*-Ar-C_i), 140.0 (Ar-C_b), 137.0 (*p*-Ar-C_j), 132.9 (Ar-C_g), 108.4 (*o*-Ar-C_c), 108.0 (*o*-Ar-C_h), 102.6 (*p*-Ar-C_e), 79.2 (*p*-$C_{l'}$≡CH), 78.5 (*m*-C_l≡CH), 76.2 (*m*-C≡C_mH), 75.4 (*m*-C≡$C_{m'}$H), 70.1 (Ar-C_fH$_2$-O), 60.5 (≡C-$C_{k'}$H$_2$), 57.2 (≡C-C_kH$_2$), 33.6 (C_aH$_2$-Br) ppm.

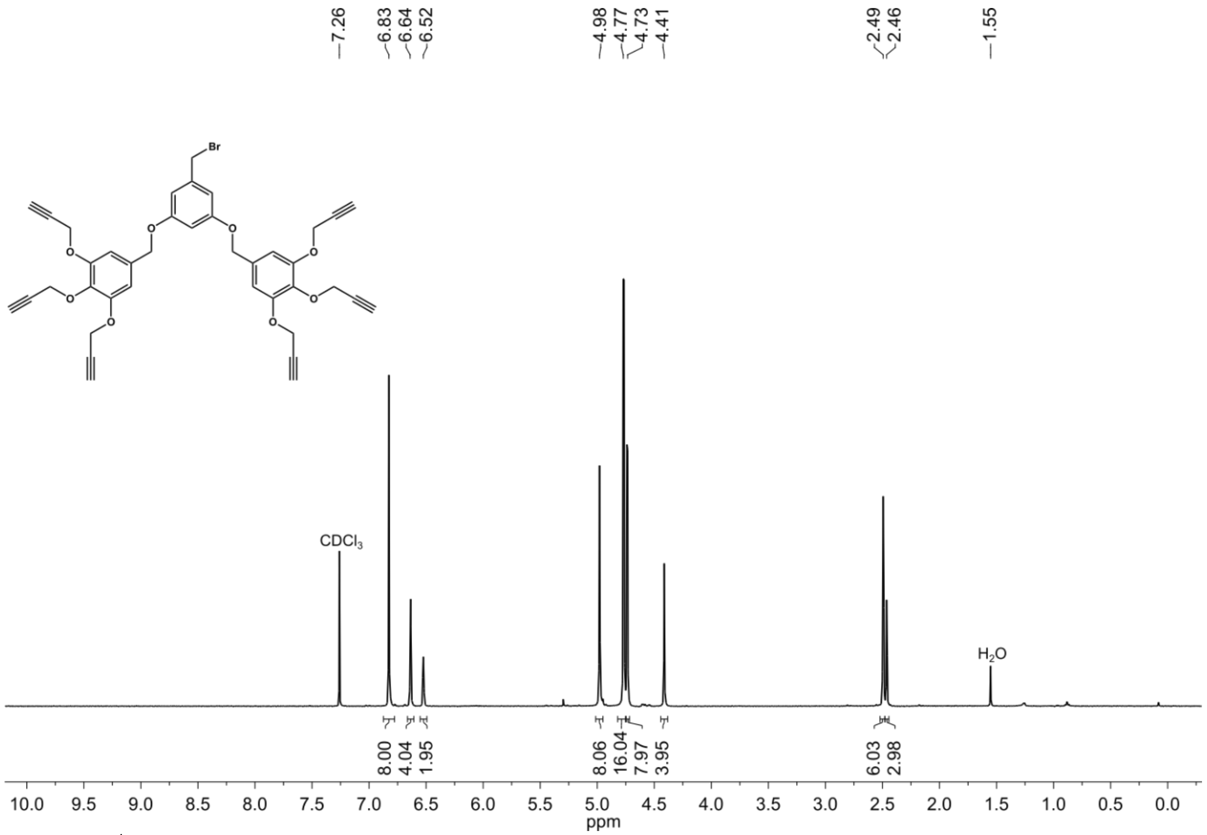

Figure S31. ^{1}H NMR spectrum of (5-(bromomethyl)-1,3-phenylene)bis(1,2,3-tris(propynyloxy)benzene) $CDCl_3$, 400 MHz, 25 °C).

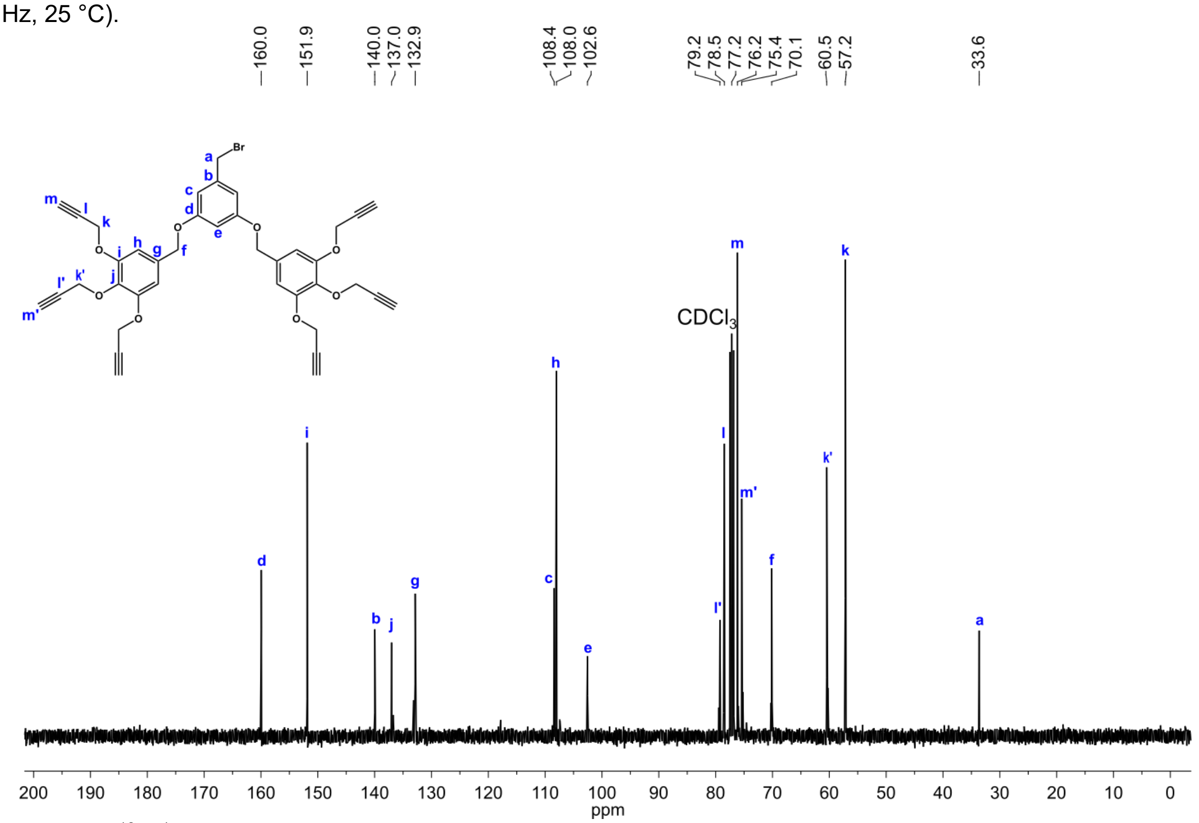

Figure S32. $^{13}C\{^1H\}$ NMR spectrum of (5-(bromomethyl)-1,3-phenylene)bis(1,2,3-tris(propynyloxy)benzene) ($CDCl_3$, 100 MHz, 25 °C).

S2.1.18. 5-(1,3-bis(3,4,5-tris(propynyloxy)))picolinaldehyde (c)

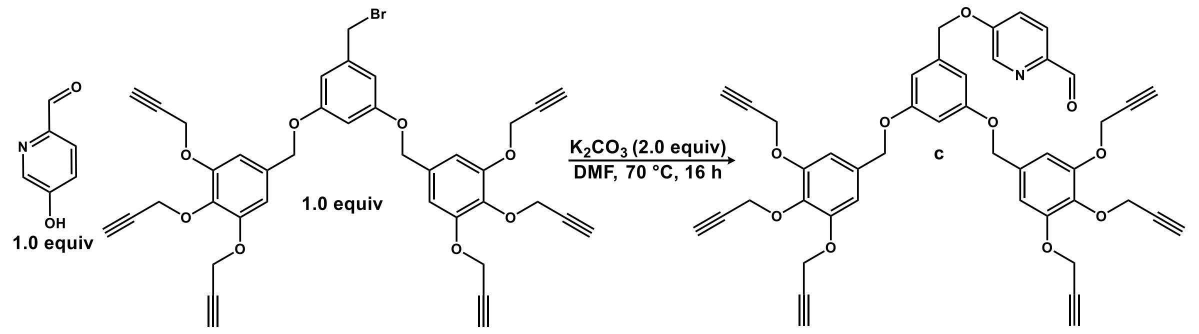

In a 50 mL Schlenk flask under an atmosphere of N_2, a DMF (10 mL) solution of (5-(bromomethyl)-1,3-phenylene)bis(1,2,3-tris(propynyloxy)benzene) (493 mg, 0.697 mmol, 1.00 equiv), 5-hydroxypicolinaldehyde (86 mg, 0.740 mmol, 1.0 equiv), and K_2CO_3 (192 mg, 1.39 mmol, 2.00 equiv) was heated at 70 °C for 16 h. The reaction mixture was allowed to cool to ambient temperature, diluted with EtOAc (150 mL) and washed with a saturated aqueous solution of NaCl (3 x 150 mL). The organic phase was dried over $MgSO_4$, filtered through a pad of Celite and evaporated to dryness to yield a brown oil, which was purified by flash liquid chromatography on silica gel eluting with a solvent gradient of 30:70 to 60:40 EtOAc/hexanes to yield 5-(1,3-bis(3,4,5-tris(propynyloxy)))picolinaldehyde (c) as a colorless powder (449 mg, 0.599 mmol, 81%). M.p. 67–70 °C. ^{1}H NMR (400 MHz, 25 °C, $CDCl_3$) δ: 9.99 (s, 1H, OC*H*), 8.49 (d, 1H, ^4J = 2.7 Hz, py-*H*-6), 7.95 (d, 1H, ^3J = 8.6 Hz, py-*H*-3), 7.33 (dd, 1H, ^3J = 8.6 Hz, ^4J = 2.8 Hz, py-*H*-4), 6.82 (s, 4H, *o*-Ar-*H*), 6.66 (d, 2H, ^4J = 2.2 Hz, *o*-Ar-*H*), 6.58 (t, 1H, ^4J = 2.2 Hz, *p*-Ar-H), 5.14 (s, 2H, CH_2-O-py), 4.98 (s, 4H, ArCH_2O), 4.76 (d, 8H, ^4J = 2.4 Hz, *m*-CH_2C≡C), 4.74 (d, 4H, ^4J = 2.4 Hz, *p*-CH_2C≡C), 2.48 (t, 4H, ^4J = 2.4 Hz, *m*-C≡C*H*), 2.46 (t, 2H, ^4J = 2.4 Hz, *p*-C≡C*H*) ppm. $^{13}C\{^1H\}$ NMR (100 MHz, 25 °C, $CDCl_3$) δ: 191.9 (OC_aH), 160.0 (*m*-Ar-C_j), 157.8 (py-C_b-2), 151.6 (*m*-Ar-C_o), 146.4 (py-C_e-5), 139.0 (py-C_f-6), 137.6 (Ar-C_h), 136.7 (*p*-Ar-C_p), 132.6 (Ar-C_m), 123.3 (py-C_c-3), 121.0 (py-C_d-4),107.7 (*o*-Ar-C_n), 106.5 (*o*-Ar-C_i), 102.0 (*p*-Ar-C_k), 79.1 (*p*-$C_{r'}$≡CH), 78.3 (*m*-C_r≡CH), 76.1 (*m*-C≡C_sH), 75.4 (*m*-C≡$C_{s'}$H), 70.4 (Ar-$C_g$$H_2$-Opy), 69.9 ($C_l$$H_2$-O), 60.2 (≡C-$C_{q'}$$H_2$), 57.0 (≡C-$C_q$$H_2$) ppm. ATR-IR ($\nu$): 3269, 2121 (C≡C), 1700 (HC=O), 1596, 1454, 1371, 1267, 1156, 1111, 1021, 987, 831, 723, 675 cm^{-1}.

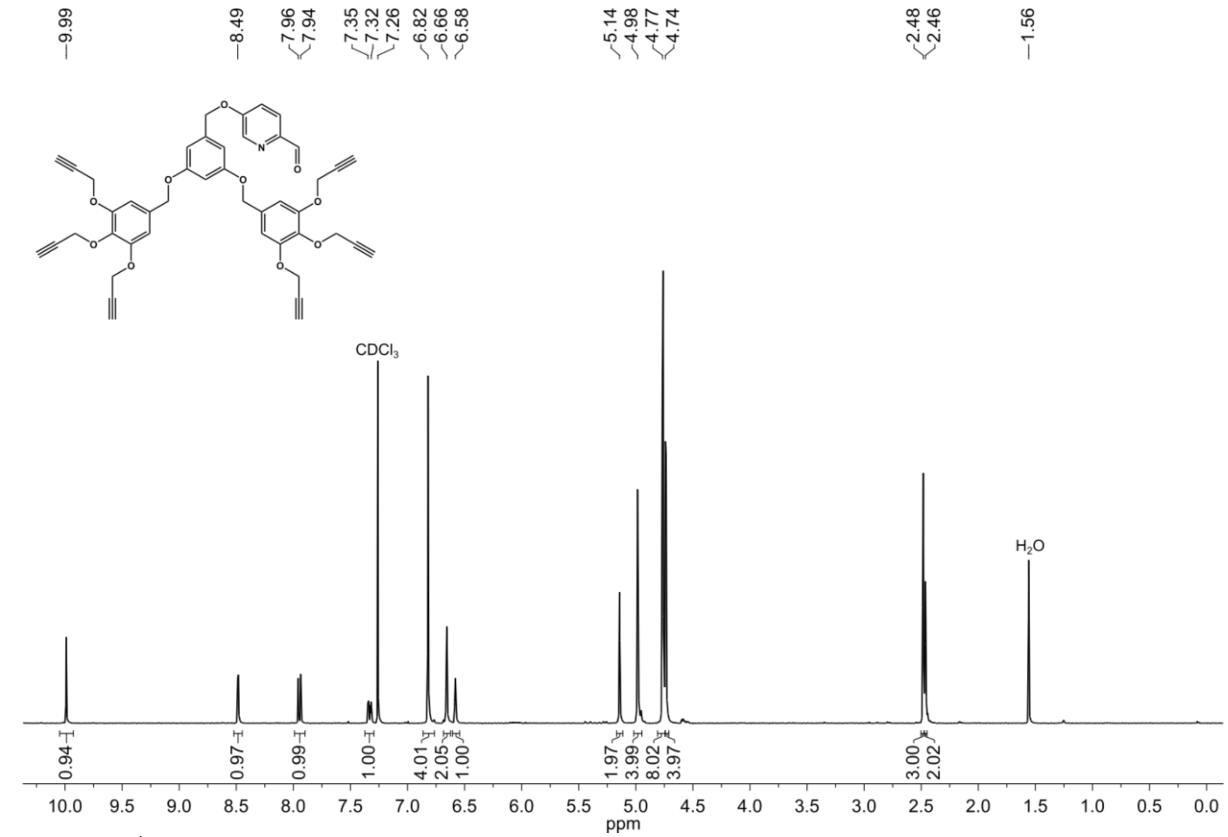

Figure S33. ^{1}H NMR spectrum of 5-(1,3-bis(3,4,5-tris(propynyloxy)))picolinaldehyde ($CDCl_3$, 400 MHz, 25 °C).

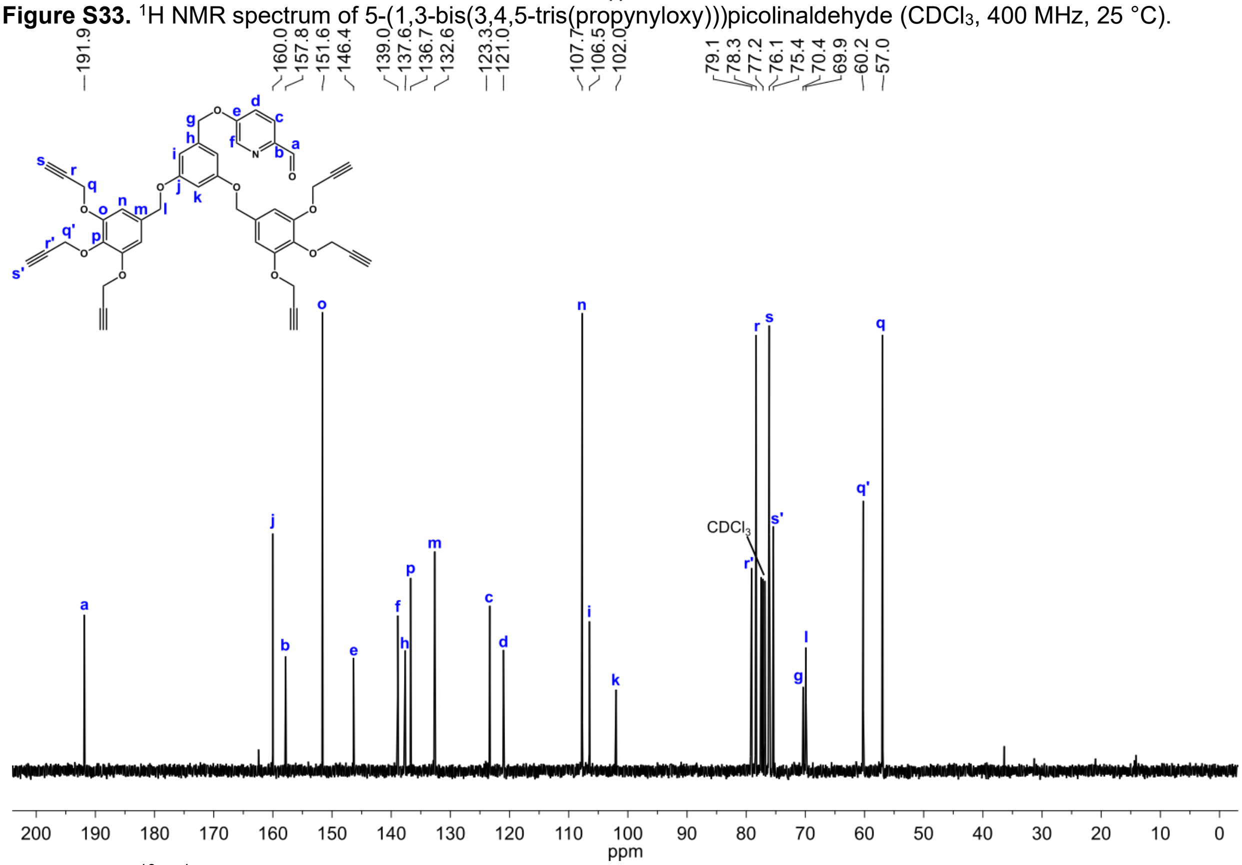

Figure S34. $^{13}C\{^{1}H\}$ NMR spectrum of 5-(1,3-bis(3,4,5-tris(propynyloxy)))picolinaldehyde ($CDCl_3$, 100 MHz, 25 °C).

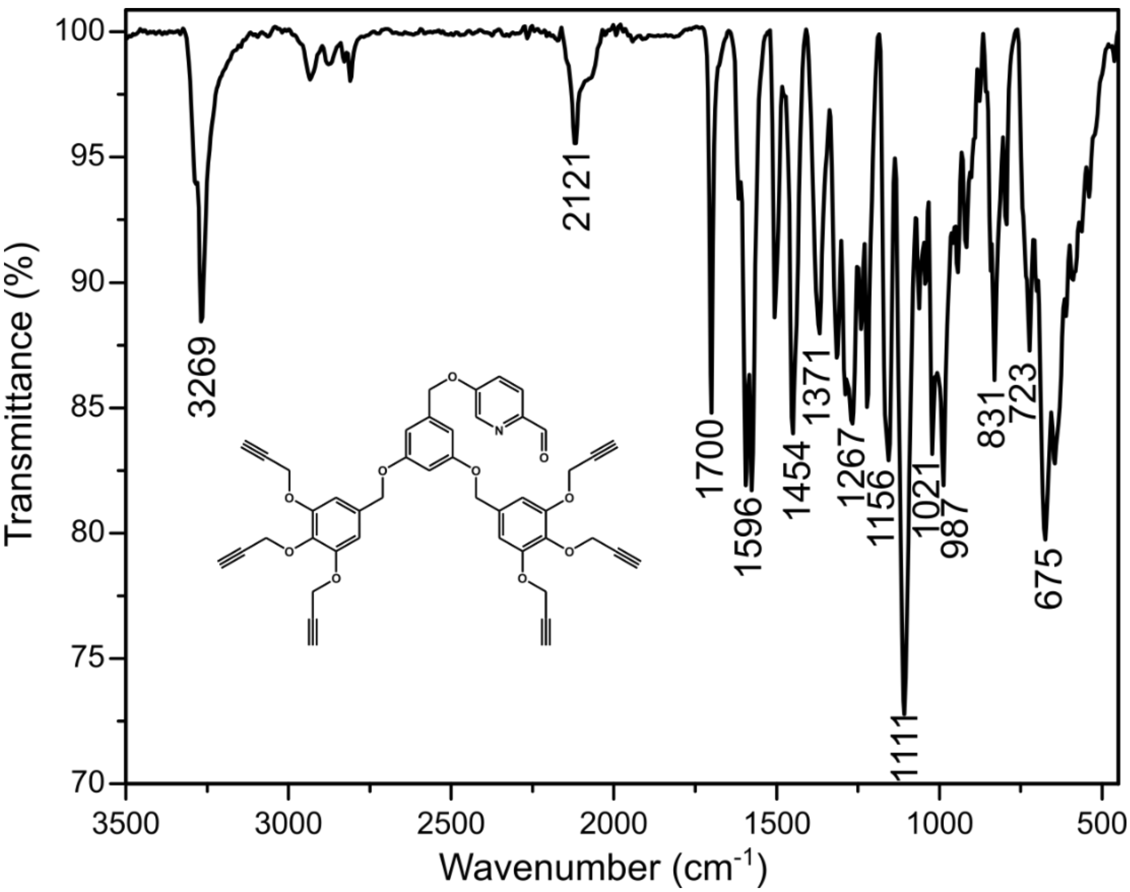

Figure S35. ATR-IR spectrum of 5-(1,3-bis(3,4,5-tris(propynyloxy)))picolinaldehyde.

S2.1.19. Man-6(OAc)

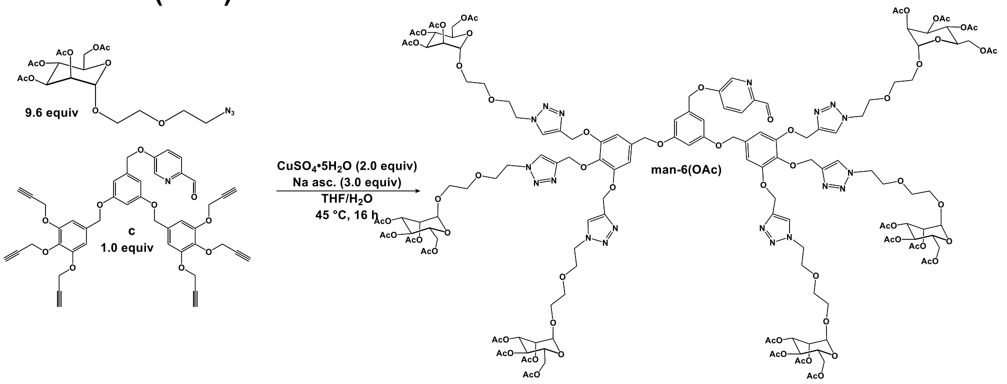

To a solution of $CuSO_4$•$5H_2O$ (45 mg, 0.87 mmol, 9.6 equiv) in H_2O (1 mL) in a 25 mL Schlenk flask was added a solution of 5-(1,3-bis(3,4,5-tris(propynyloxy)))picolinaldehyde (c) (68 mg, 0.091 mmol, 1.0 equiv), sodium ascorbate (54 mg, 0.27 mmol, 3.0 equiv), and 2-(2-azidoethoxy)ethyl(OAc)$_4$-α-D-mannose (400 mg, 0.870 mmol, 9.60 equiv) in THF (10 mL). The yellow heterogeneous reaction mixture was allowed to stir under an atmosphere of N_2 for 16 h, at which point it was allowed to cool to ambient temperature. The mixture was diluted with EtOAc (150 mL) and washed with an aqueous solution of Na_4[EDTA] (3 x 30 mL), followed by a saturated aqueous solution of NaCl (3 x 150 mL). The organic phase was dried over $MgSO_4$, filtered through a pad of Celite and evaporated to dryness to yield a yellow oil that was purified on silica gel eluting first with a 60:40 EtOAc/hexanes mixture (100 mL) to remove unreacted 2-(2-azidoethoxy)ethyl(OAc)$_4$-α-D-mannose followed by acetone (50 mL). The acetone fraction was evaporated, and the residue was purified by flash liquid chromatography eluting with a solvent system of 0:100 to 5:95 MeOH/DCM to yield man-6(OAc) as a colorless powder (208 mg, 0.059 mmol, 65%). M.p. 61–64 °C. ^{1}H NMR (400 MHz, 25 °C, $CDCl_3$) δ: 9.95 (s, 1H, OC*H*), 8.48 (d, 1H, ^4J = 2.4 Hz, py-*H*-6), 7.92 (d, 1H, ^3J = 8.6 Hz, py-*H*-3), 7.90 (s, 4H, N-C*H*), 7.89 (s, 2H, N-C*H*), 7.34 (dd, 1H, ^3J = 8.7 Hz, ^4J = 2.4 Hz, py-*H*-4), 6.83 (s, 4H, *o*-Ar-*H*), 6.65 (d, 2H, ^4J = 1.8 Hz, *o*-Ar-*H*), 6.60 (t, 1H, ^4J = 1.8 Hz, *p*-Ar-*H*), 5.30 (dd, 6H, *J* = 10.1, 3.1 Hz, man-*H*-3), 5.26 (d, 6H, *J* = 10.1 Hz, man-*H*-2), 5.22 (m, 6H, man-*H*-4), 5.20 (s, 8H, *m*-OCH_2), 5.17 (s, 2H, CH_2-Opy), 5.16 (s, 4H, *p*-OCH_2), 5.14 (s, 4H, CH_2-OAr), 4.94 (m, 4H, OCH_2), 4.82 (d, 6H, *J* = 1.1 Hz, man-*H*-1), 4.55 (t, 8H, *J* = 5.1 Hz, OCH_2), 4.51 (t, 4H, OCH_2, *J* = 5.1 Hz), 4.26 (t, 2H, *J* = 5.0 Hz, man-*H*-5'), 4.22 (t, 4H, *J* = 5.0 Hz, man-*H*-5), 4.07 (t, 4H, *J* = 2.8 Hz, man-*H*-6$_a$), 4.04 (t, 2H, *J* = 2.8 Hz, man-*H*-6$_a$), 3.98-3.95 (m, 6H, man-*H*-6$_b$), 3.91-3.86 (m, 12H, OCH_2), 3.77-3.73 (m, 6H, OCH_2), 3.62-3.58 (m, 14H, OCH_2), 2.11 (s, 6H, CH_3COO), 2.11 (s, 12H, CH_3COO), 2.05 (s, 18H, CH_3COO, two signals overlapping), 2.01 (s, 18H, CH_3COO, two signals overlapping), 1.95 (s, 6H, CH_3COO), 1.94 (s, 12H, CH_3COO) ppm. ^{13}C{^{1}H} NMR (100 MHz, 25 °C, $CDCl_3$) δ: 192.1 (OC_aH), 170.7 (CH_3*C*OO), 170.2 (CH_3*C*OO), 170.11 (CH_3*C*OO), 170.13 (CH_3*C*OO), 170.03 (CH_3*C*OO), 170.00 (CH_3*C*OO), 169.83 (CH_3*C*OO), 169.82 (CH_3*C*OO), 160.3 (*m*-Ar-C_j), 158.1 (py-C_b-2), 152.6 (*m*-Ar-C_o), 146.6 (py-C_e-5), 144.7 (*p*-C=$C_{r'}$N), 143.8 (*m*-C=C_rN), 139.2 (py-C_f-6), 137.9 (Ar-C_h), 137.6 (*p*-Ar-C_p), 132.6 (Ar-C_m), 124.7 (py-C_c-3), 124.4 (*m*-N-C_s=C), 123.5 (*p*-N-$C_{s'}$=C), 121.3 (py-C_d-4), 107.3 (*o*-Ar-C_n), 106.6 (*o*-Ar-C_i), 101.8 (*p*-Ar-C_k), 97.8 (man-*C*-1, two signals overlapping), 70.6 ($C_g$$H_2$-O-py), 70.1 (O*C*$H_2$, two signals overlapping), 69.6 (O*C*H_2, two signals overlapping), 69.5 (man-*C*-2, two signals overlapping), 69.1 (man-*C*-3, two signals overlapping), 68.6 (man-*C*-5, two signals overlapping), 67.3 (man-*C*-4, two signals overlapping), 67.3 (O*C*H_2, two signals overlapping), 66.2 (O*C*H_2, two signals overlapping), 63.2 (man-*C*-6, two signals overlapping), 62.5 (man-*C*-6, two signals overlapping), 50.3 (*m*-ArO$C_q$$H_2$), 50.0 (*p*-ArO$C_{q'}$$H_2$), 21.0 ($CH_3$*C*OO, two signals overlapping), 20.83 (CH_3*C*OO, two signals overlapping), 20.78 (CH_3*C*OO, two signals overlapping), 20.76, 20.7 ppm *(two decimal places are given only where needed to distinguish resonances)*. HR-ESI-MS(+) (*m/z*): 1781.6144 ([M+2Na]$^{2+}$, calc'd, 1781.5974).ATR-IR (ν): 2937, 1741 (HC=O), 1595, 1439, 1372, 1219, 1137, 1044, 977, 831, 600 cm^{-1}.

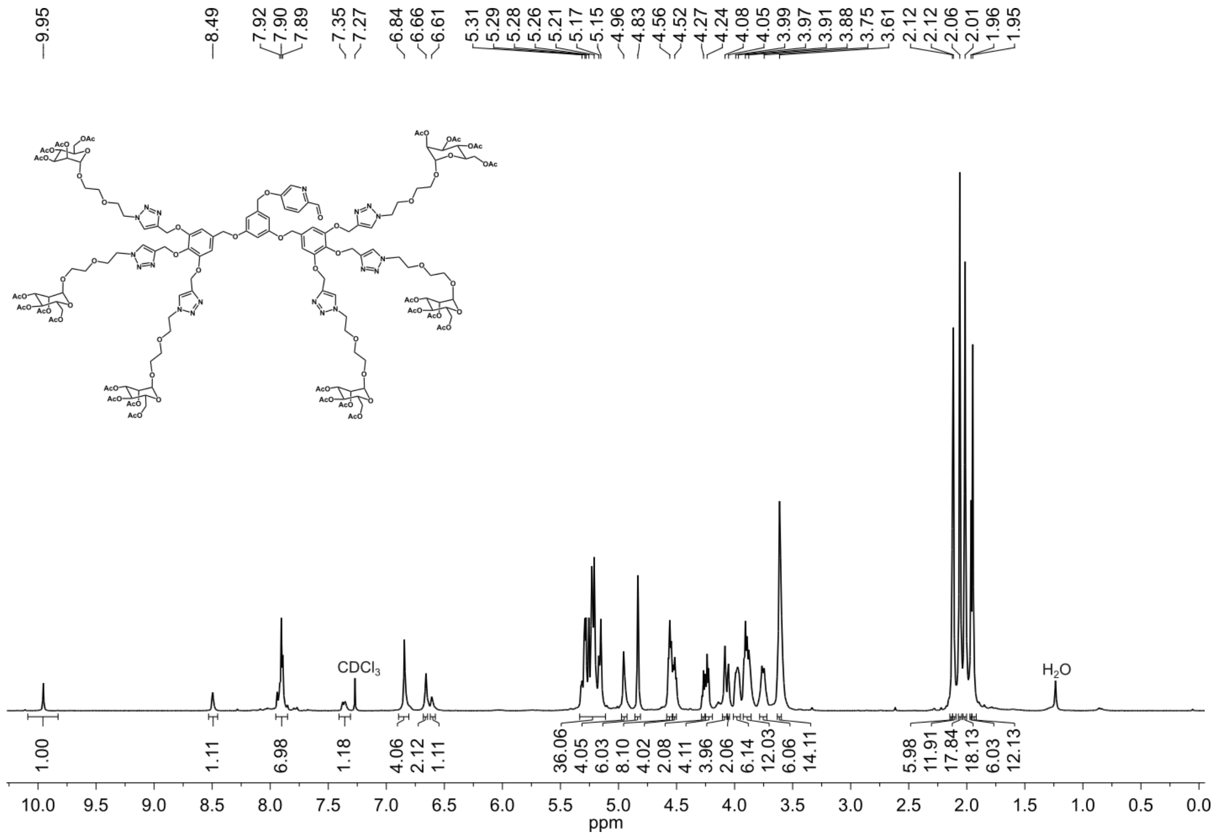

Figure S36. ^{1}H NMR spectrum of man-6(OAc) ($CDCl_3$, 400 MHz, 25 °C).

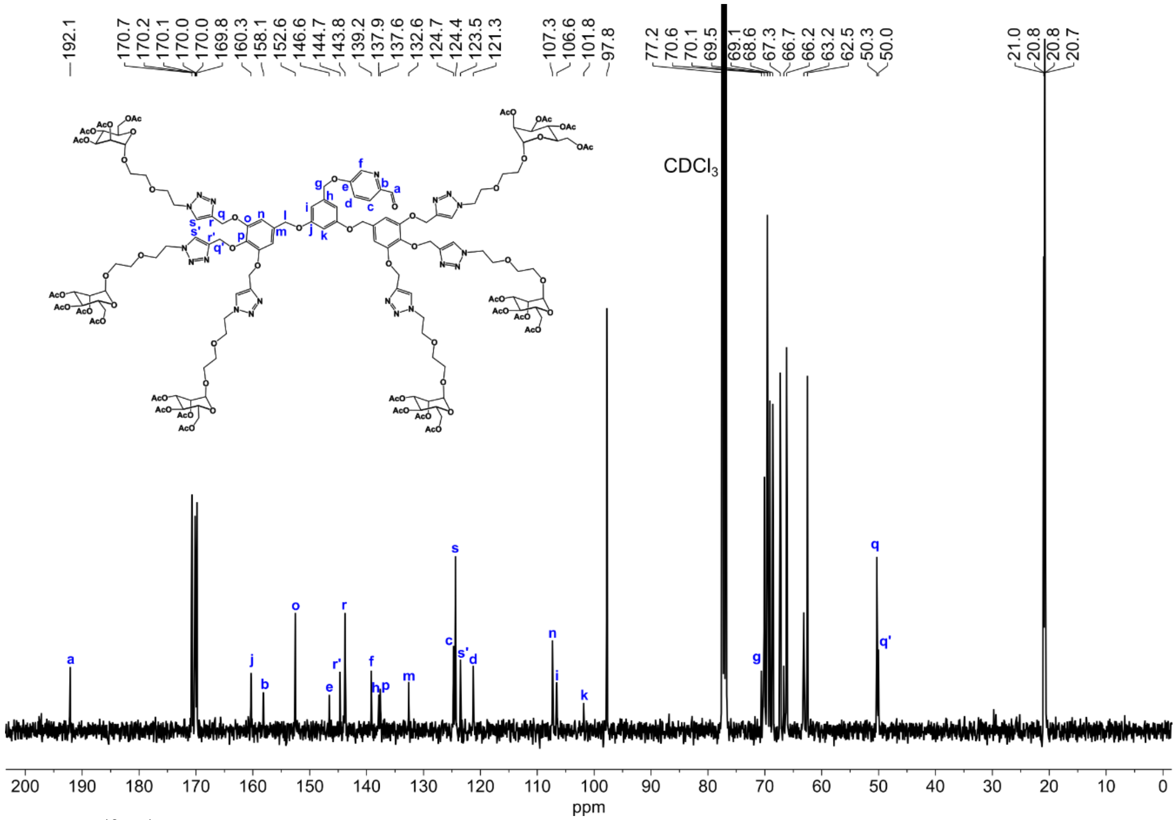

Figure S37. $^{13}C\{^{1}H\}$ NMR spectrum of man-6(OAc) ($CDCl_3$, 100 MHz, 25 °C).

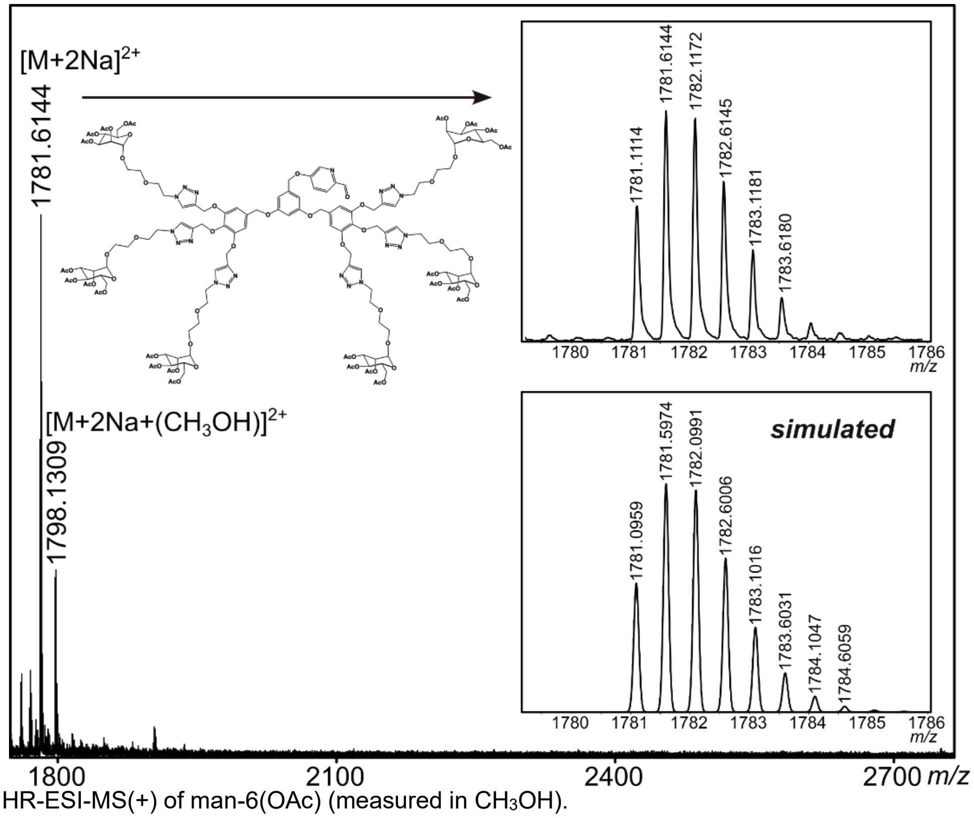

Figure S38. HR-ESI-MS(+) of man-6(OAc) (measured in CH_3OH).

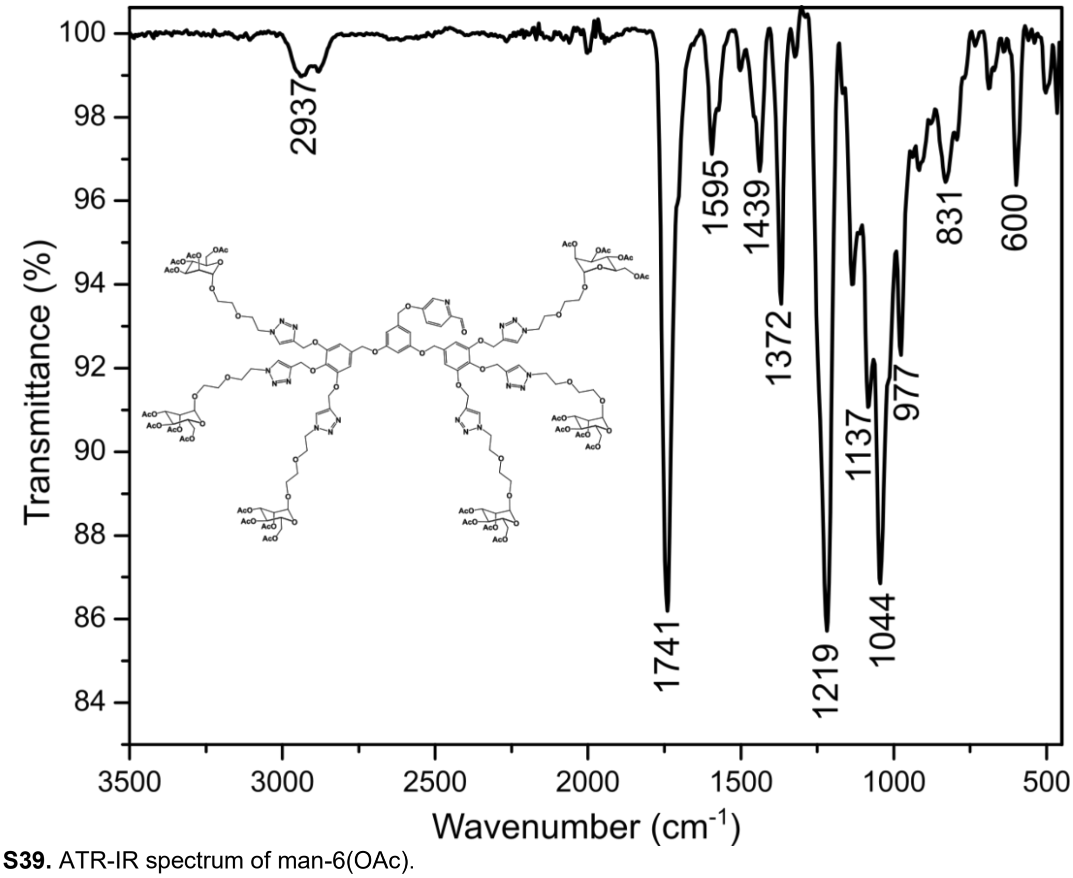

Figure S39. ATR-IR spectrum of man-6(OAc).

S2.1.20. Man-6

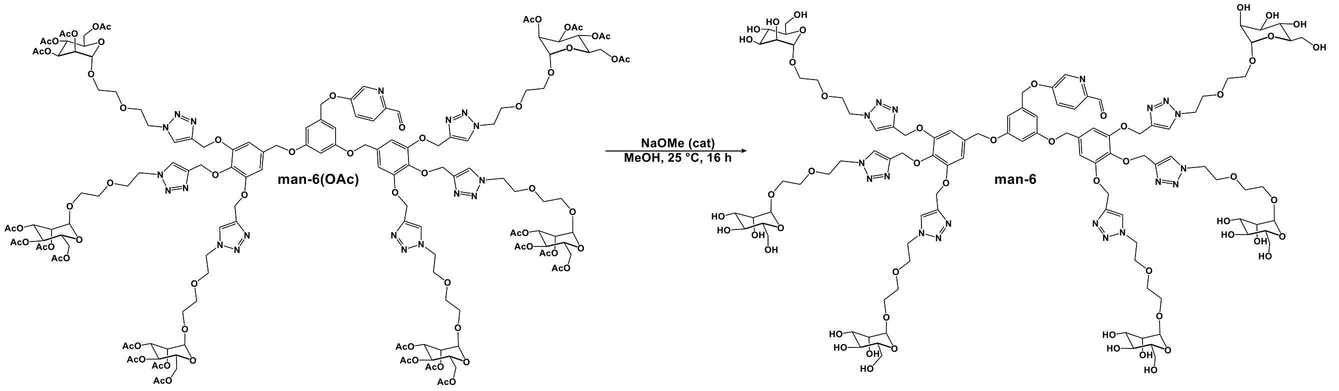

To a stirring solution of man-6(OAc) (80 mg, 0.023 mmol, 1.0 equiv) in MeOH (20 mL) was added a solution of NaOMe in MeOH until the pH reached 8 (400 µL of a 0.3 M solution, 0.12 mmol, 5.2 equiv). The reaction mixture was allowed to stir at ambient temperature under an atmosphere of N_2 for 16 h, at which point it was neutralized with Amberlyst®-15 (180 mg). The solution was filtered through a pad of Celite, and all volatiles were removed under reduced pressure to afford man-6 as a colorless solid that was used without further purification (yield: 40 mg, 0.016 mmol, 69%). ^{1}H NMR (400 MHz, 25 °C, D_2O) δ: 9.97 (br s, 1H, OC*H*), 8.61 (br s, 1H, py-*H*-6), 8.10 (br s, 4H, N-C*H*), 7.80 (br s, 2H, N-C*H*), 7.38 (br s, 1H, *p*-Ar), 7.15 (d, 1H, ^3J = 8.3 Hz, py-*H*-3), 6.94 (d, 1H, ^3J = 8.3 Hz, py-*H*-4), 6.75 (br s, 4H, *o*-Ar), 6.57 (br s, 2H, *o*-Ar), 4.95 (s, 6H, man-*H*-1), 4.49 (s, 16H, –CH_2–), 3.83 (m, 36H, man-*H*-2, man-*H*-6_b, man-*H*-6_a, man-*H*-3, –CH_2– signals overlapping), 3.72 (m, 32H, man-*H*-4, man-*H*-5, –CH_2– signals overlapping), 3.53 (m, 18H, –CH_2–) ppm. ^{1}H NMR (400 MHz, 25 °C, CD_3OD) δ: 9.91 (s, 1H, OC*H*), 8.49 (d, 1H, ^4J = 2.4 Hz, py-*H*-6), 8.25 (d, 1H, ^3J = 8.6 Hz, py-*H*-3), 8.12 (s, 4H, N-C*H*), 8.10 (s, 2H, N-C*H*), 7.57 (dd, 1H, ^3J = 8.7 Hz, ^4J = 2.4 Hz, py-*H*-4), 7.55 (d, 2H, ^4J = 1.8 Hz, *o*-Ar-*H*), 7.48 (t, 1H, ^4J = 1.8 Hz, *p*-Ar-*H*), 6.97 (s, 4H, *o*-Ar), 5.23 (s, 4H, *m*-OCH_2), 5.20 (d, 4H, *J* = 1.1 Hz, man-*H*-1), 5.13 (s, 2H, CH_2-Opy), 5.10 (d, 2H, *J* = 1.1 Hz, man-*H*-1), 4.91 (s, 12H, *m*-OCH_2, *p*-OCH_2 overlapping with H_2O), 4.71 (m, 6H, man-*H*-2), 4.67-4.58 (m, 18H, –CH_2–), 4.15-4.04 (m, 6H, man-*H*-2), 3.88-3.82 (m, 6H, man-*H*-6_a), 3.77-3.72 (m, 12H, man-*H*-6_b, –CH_2–), 3.67-3.56 (m, 32H, man-*H*-2, man-*H*-3, man-*H*-5, –CH_2– signals overlapping), 3.33 (m, 4H, –CH_2–) 3.23-3.20 (m, 6H, –CH_2–) ppm. ESI-MS(+) (*m/z*): 837.00 ([M+3H]$^{3+}$, calc'd, 836.99).

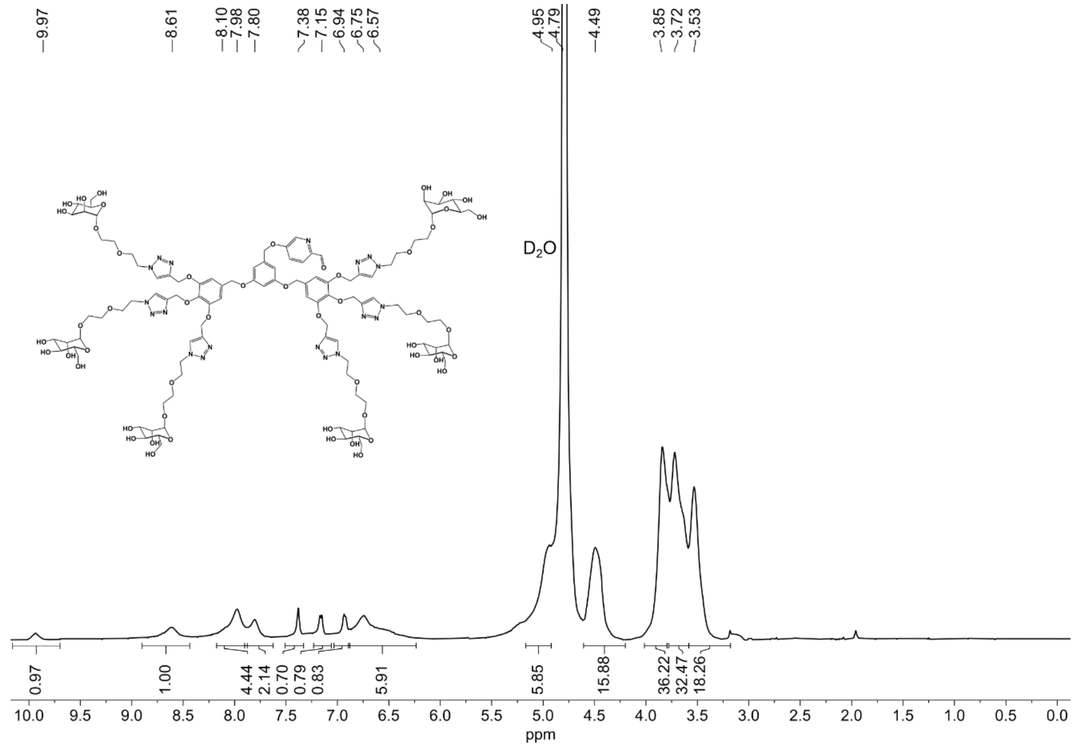

Figure S40. ^{1}H NMR spectrum of man-6 (D_2O, 400 MHz, 25 °C).

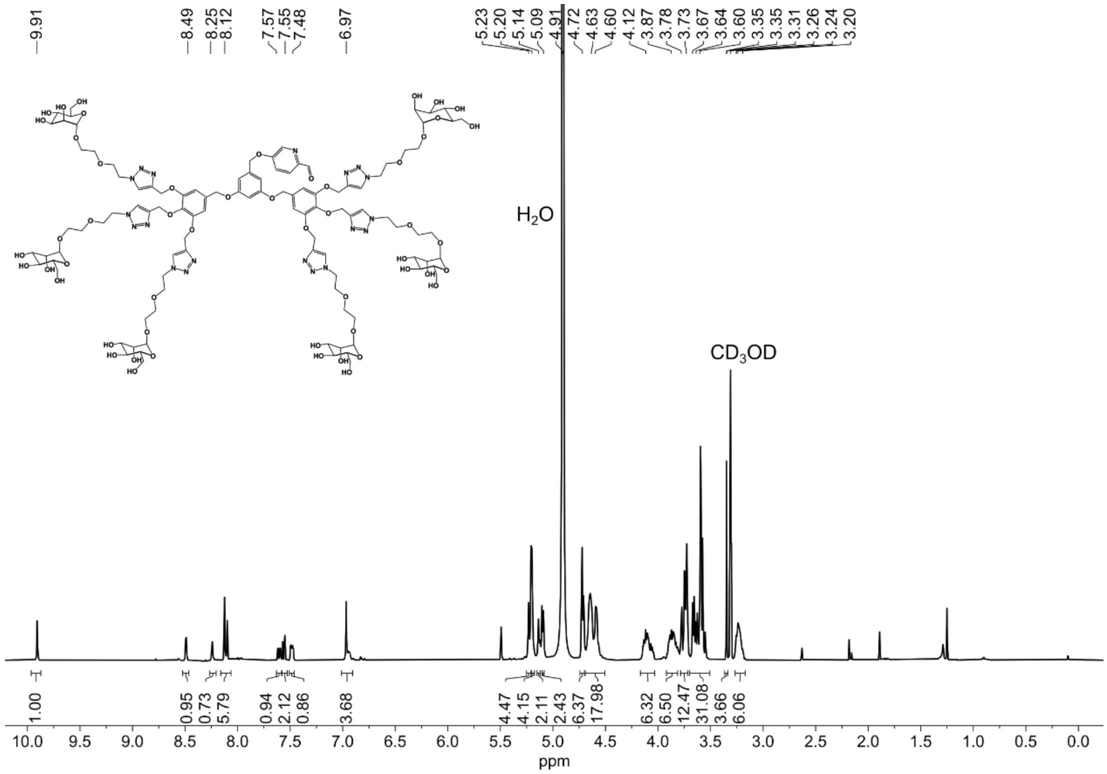

Figure S41. ^{1}H NMR spectrum of man-6 (CD_3OD, 400 MHz, 25 °C).

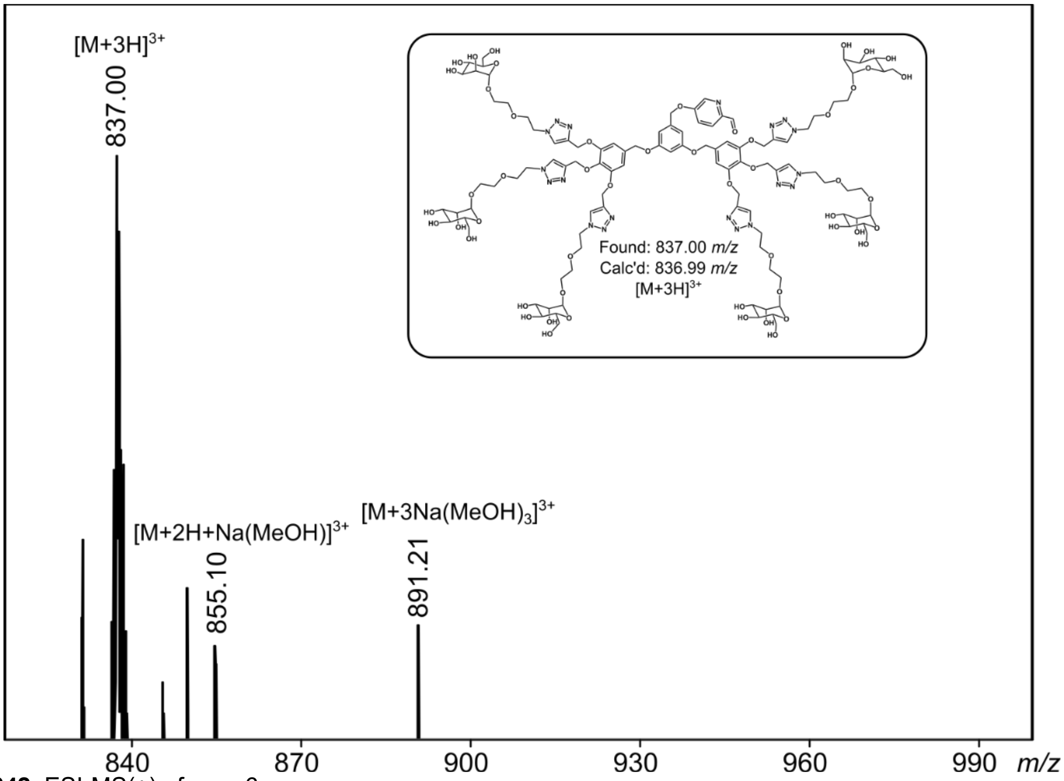

Figure S42. ESI-MS(+) of man-6.

S2.2. Fe(II) mannosylated self-assembly complexes

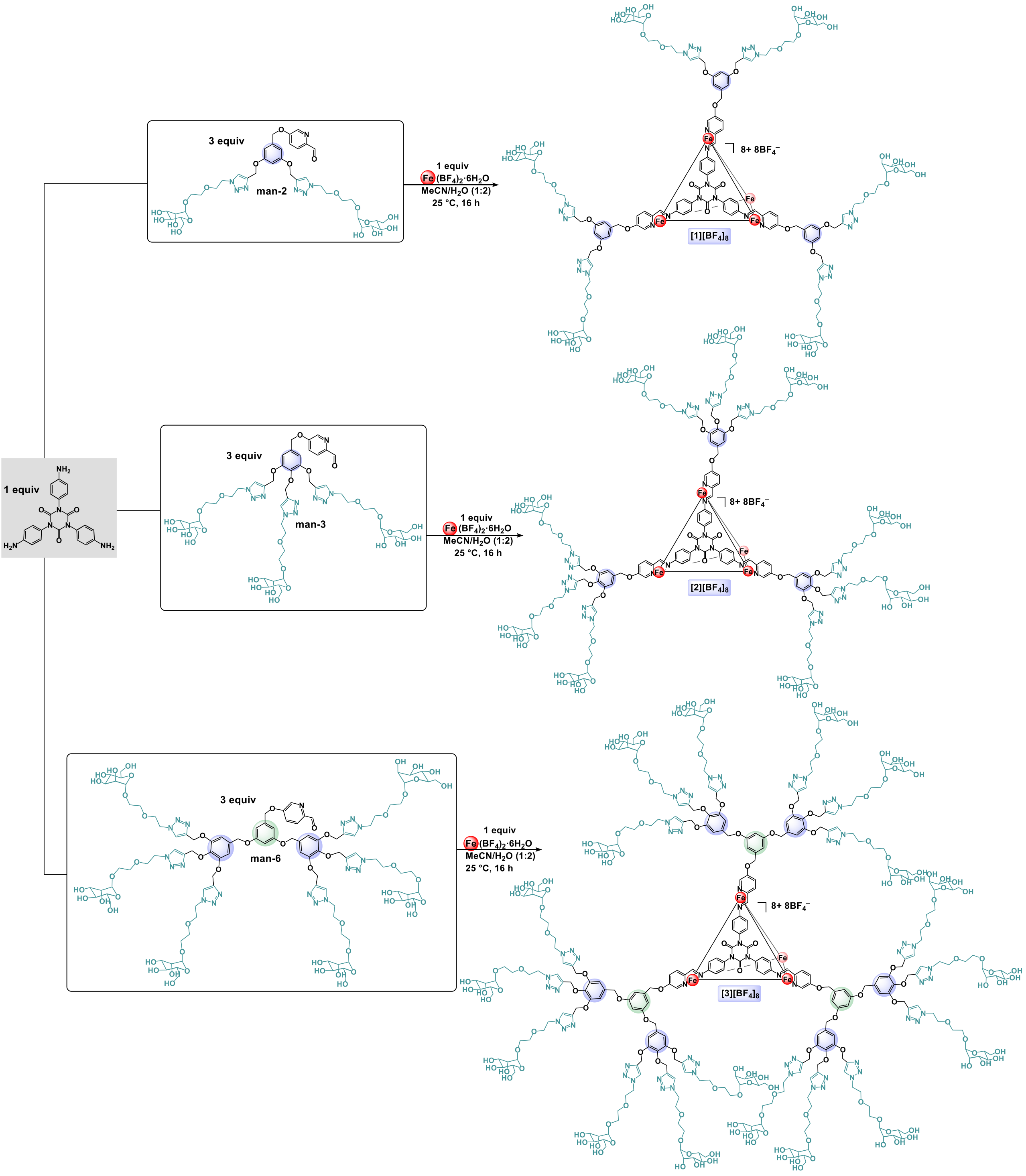

Figure S43. Overview of self-assembly reactions to prepare [**1**][BF4]8, [**2**][BF4]8, and [**3**][BF4]8. Only one of four face-capping ligands is shown for clarity.

S2.2.1. [1][BF_4]$_8$

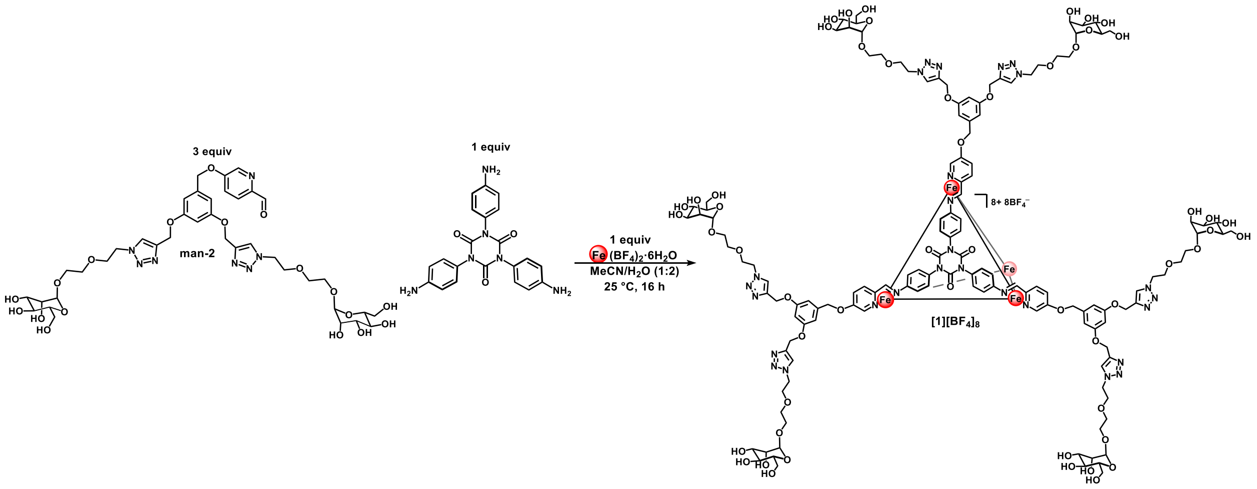

An 8 mL reaction tube was charged with a degassed solution of 2-man (48 mg, 0.053 mmol, 3.0 equiv) in H_2O (1.0 mL). To this solution was added 1,3,5-tris(4-aminophenyl)-1,3,5-triazinane-2,4,6-trione (7 mg, 0.02 mmol, 1 equiv) followed by a degassed solution of MeCN (0.5 mL), and the pale orange solution was allowed to stir at ambient temperature for 10 min under a flow of N_2. To the solution was added Fe(BF_4)$_2$•6H_2O (6 mg, 0.02 mmol, 1 equiv) as a solid, which resulted in an immediate color change to magenta. The reaction mixture was sparged with N_2 for 15 min and was then allowed to stir at 25 °C for 16 h, at which point the magenta solution was filtered through a piece of glass microfiber filter paper and all volatiles were removed under reduced pressure. The pink residue was then dissolved in H_2O (5 mL), and the solution was transferred to the sample reservoir of a Amicon® Ultra Centrifugal Filter (10 kDa MWCO, cellulose membrane). The device was centrifuged at 7500 x g for 75 min, at which point the filtrate in the collection tube was discarded and fresh H_2O (5 mL) was added to the sample reservoir. The device was centrifuged again at 7500 x g for 75 min, and this process was repeated for a total of three centrifuge cycles. After the third cycle, the solution in the sample reservoir was removed and lyophilized overnight to afford the product, [**1**][BF_4]$_8$, as a magenta powder (yield: 19 mg, 1.4 µmol, 32%). ^{1}H NMR (400 MHz, 45 °C, D_2O) δ: 9.15 (br s, 1H, *H*C=N), 8.67 (br s, 1H, py-*H*-3), 8.21 (br s, 3H, py-*H*-4, N-C*H* signals overlapping), 7.62 (br s, 1H, Ar-*H*), 7.25 (br s, 2H, *o*-Ar-*H*), 6.82 (br s, 1H, pyr-*H*-6), 6.58 (br s, 2H, Ar-*H*, *p*-Ar-*H* signals overlapping), 6.06 (br s, 1H, Ar-*H*), 5.24 (br s, 2H, man-*H*-1), 4.96 (br s, 1H, Ar-*H*), 4.07-4.00 (m, 10H, –CH_2–, man-*H*-6$_a$, man-*H*-6$_b$, man-*H*-2 signals overlapping), 4.07-4.00 (m, 10H, *m*-C*H*$_2$, C*H*$_2$-Opy, man-*H*-3, man-*H*-4, man-*H*-5 signals overlapping), 3.77–3.68 (m, 14H, –C*H*$_2$–) ppm. UV-vis [ε] (H_2O, 50 µM): λ_{max} 505 [21,000 $M^{-1}cm^{-1}$], 550 [25,000 $M^{-1}cm^{-1}$] nm. ATR-IR (ν): 3384 (OH), 2878, 1707 (C=O), 1595, 1559 (C=N), 1498, 1431, 1308, 1230, 1051, 835 cm^{-1}.

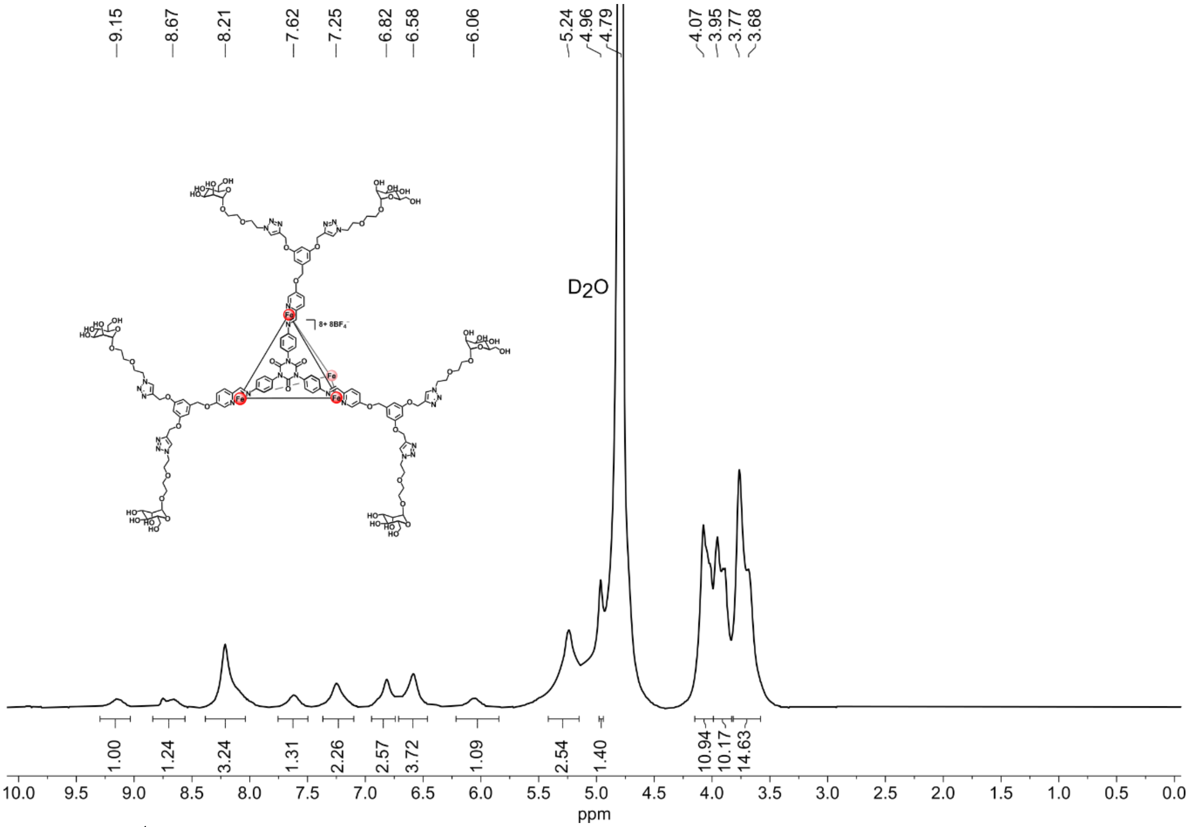

Figure S44. ^{1}H NMR spectrum of [**1**][BF_4]$_8$ (D_2O, 400 MHz, 45 °C).

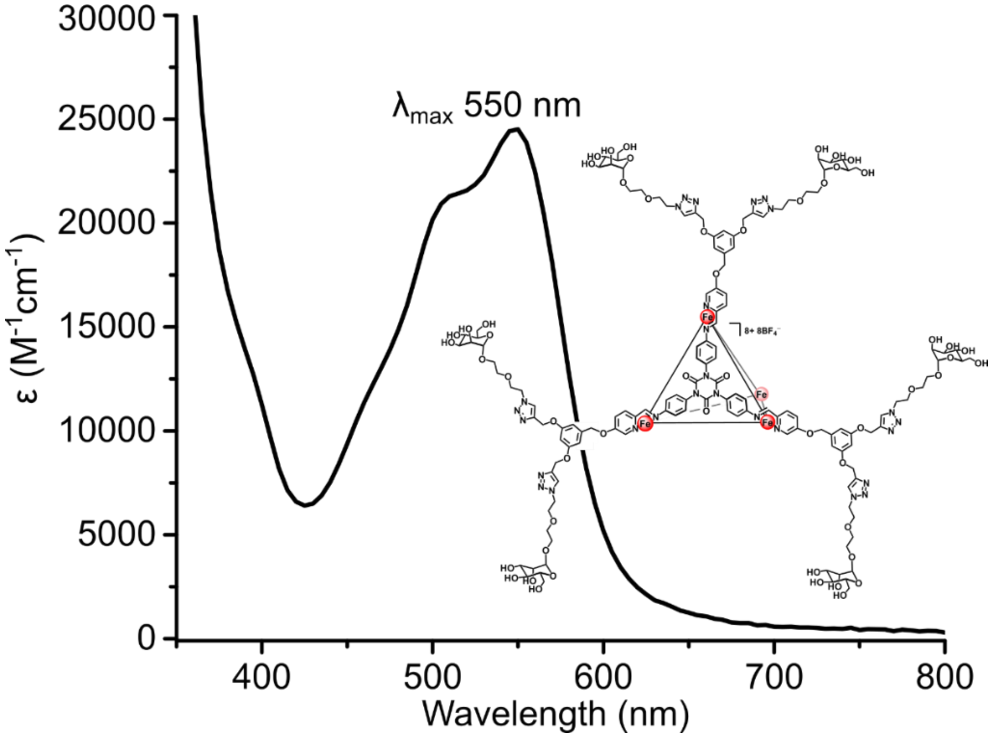

Figure S45. UV-vis spectrum of [**1**][BF_4]$_8$ (H_2O, 50 μM).

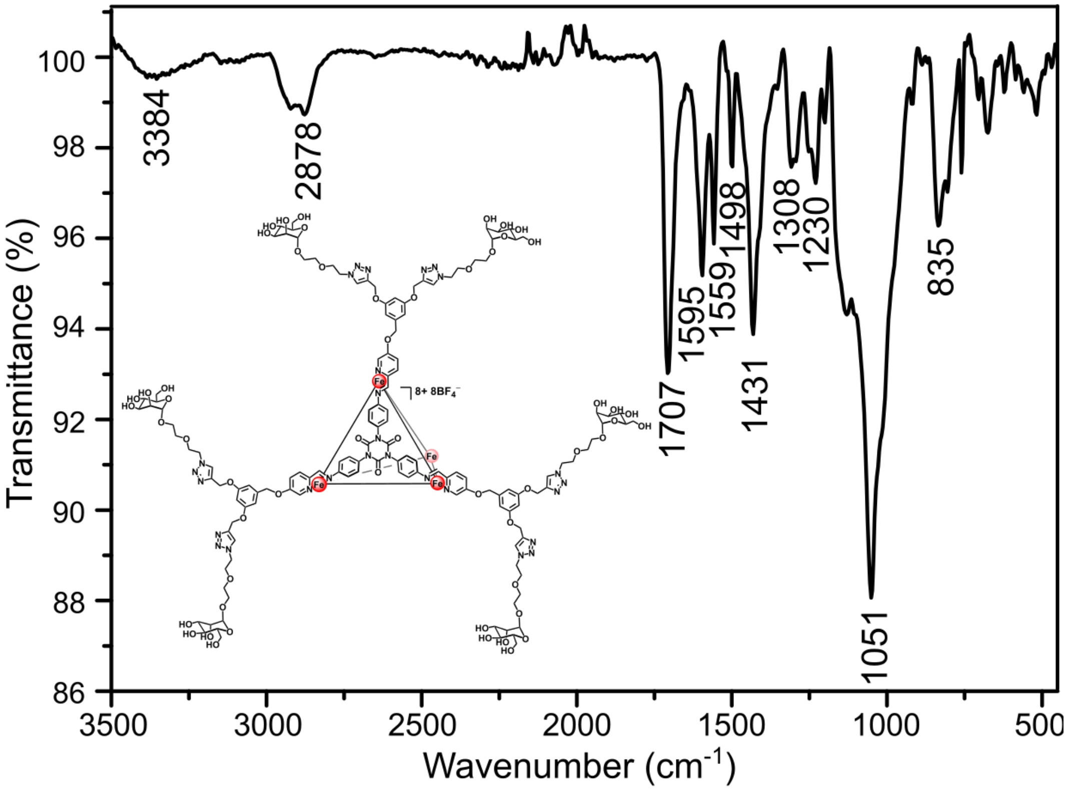

Figure S46. ATR-IR spectrum of [**1**][BF_4]$_8$.

S2.2.2. [2][BF_4]$_8$

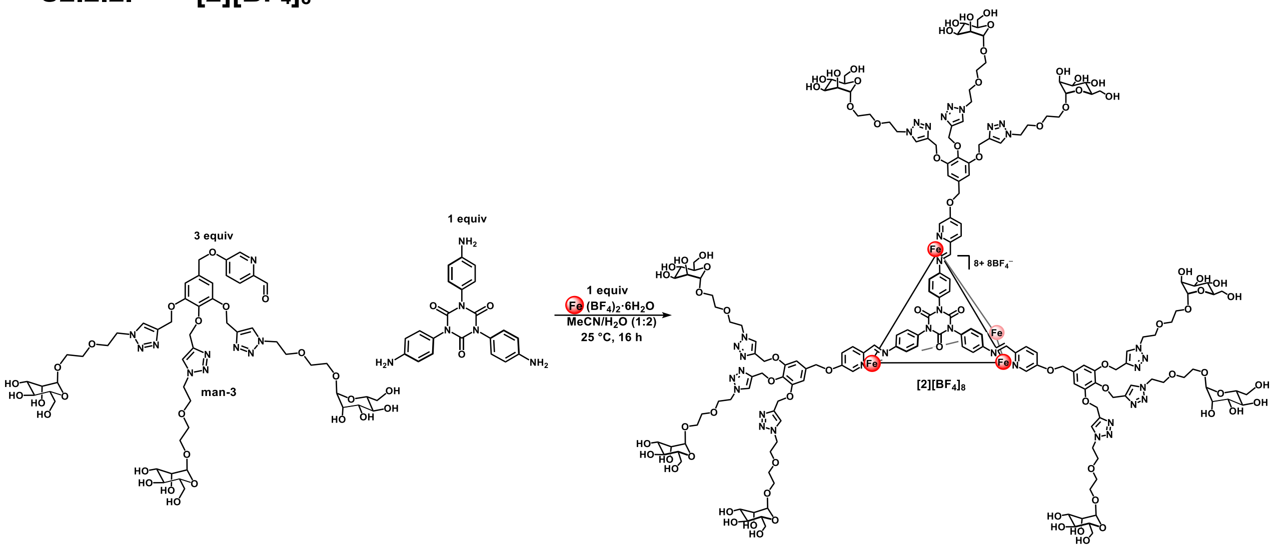

An 8 mL reaction tube was charged with a degassed solution of man-3 (43 mg, 0.034 mmol, 3.0 equiv) in H_2O (1.0 mL). To this solution was added 1,3,5-tris(4-aminophenyl)-1,3,5-triazinane-2,4,6-trione (4 mg, 0.01 mmol, 1 equiv) followed by a degassed solution of MeCN (0.5 mL), and the pale orange solution was allowed to stir at ambient temperature for 10 min under a flow of N_2. To the solution was added $Fe(BF_4)_2$•$6H_2O$ (4 mg, 0.01 mmol, 1 equiv) as a solid, which resulted in an immediate color change to magenta. The reaction mixture was sparged with N_2 for 15 min and was then allowed to stir at 25 °C for 16 h, at which point the magenta solution was filtered through a piece of glass microfiber filter paper and all volatiles were removed under reduced pressure. The pink residue was then dissolved in H_2O (5 mL), and the solution was transferred to the sample reservoir of a Amicon® Ultra Centrifugal Filter (10 kDa MWCO, cellulose membrane). The device was centrifuged at 7500 x g for 75 min, at which point the filtrate in the collection tube was discarded and fresh H_2O (5 mL) was added to the sample reservoir. The device was centrifuged again at 7500 x g for 75 min, and this process was repeated for a total of three centrifuge cycles. After the third cycle, the solution in the sample reservoir was removed and lyophilized overnight to afford the product, [**2**][BF_4]$_8$, as a magenta powder (yield: 23 mg, 1.3 μmol, 46%). ^{1}H NMR (400 MHz, 45 °C, D_2O) δ: 8.85 (br s, 1H, *H*C=N), 8.41 (br s, 1H, py-*H*-3), 7.99 (br s, 2H, N-C*H*), 7.92 (br s, 1H, py-*H*-4), 7.91 (br s, 1H, Ar-*H*), 7.74 (br s, 1H, N-C*H*), 7.36 (br s, 1H, pyr-*H*-6), 7.07 (br s, 1H, Ar-*H*), 6.97 (br s, 1H, Ar-*H*), 6.68 (br s, 2H, *o*-Ar-*H*), 5.93 (br s, 1H, Ar-*H*), 5.24 (br s, 1H, Ar-*H*), 4.94 (br s, 3H, man-*H*-1 overlapping with residual solvent signal), 4.68-4.42 (m, 7H, –CH_2, man-*H*-6$_a$), 3.85-3.75 (m, 16H, man-*H*-6$_b$, man-*H*-2, –CH_2– signals overlapping), 3.66 (m, 11H, man-*H*-3, man-*H*-4, man-*H*-5, –CH_2– signals overlapping), 3.49–3.41 (m, 16H, –CH_2–) ppm. UV-vis [ε] (H_2O, 50 μM): λ_{max} 505 [20,000 $M^{-1}cm^{-1}$], 550 [22,000 $M^{-1}cm^{-1}$] nm. ATR-IR (ν): 3347 (OH), 2923, 1707 (C=O), 1595, 1558 (C=N), 1498, 1431, 1316, 1230, 1047, 805 cm^{-1}

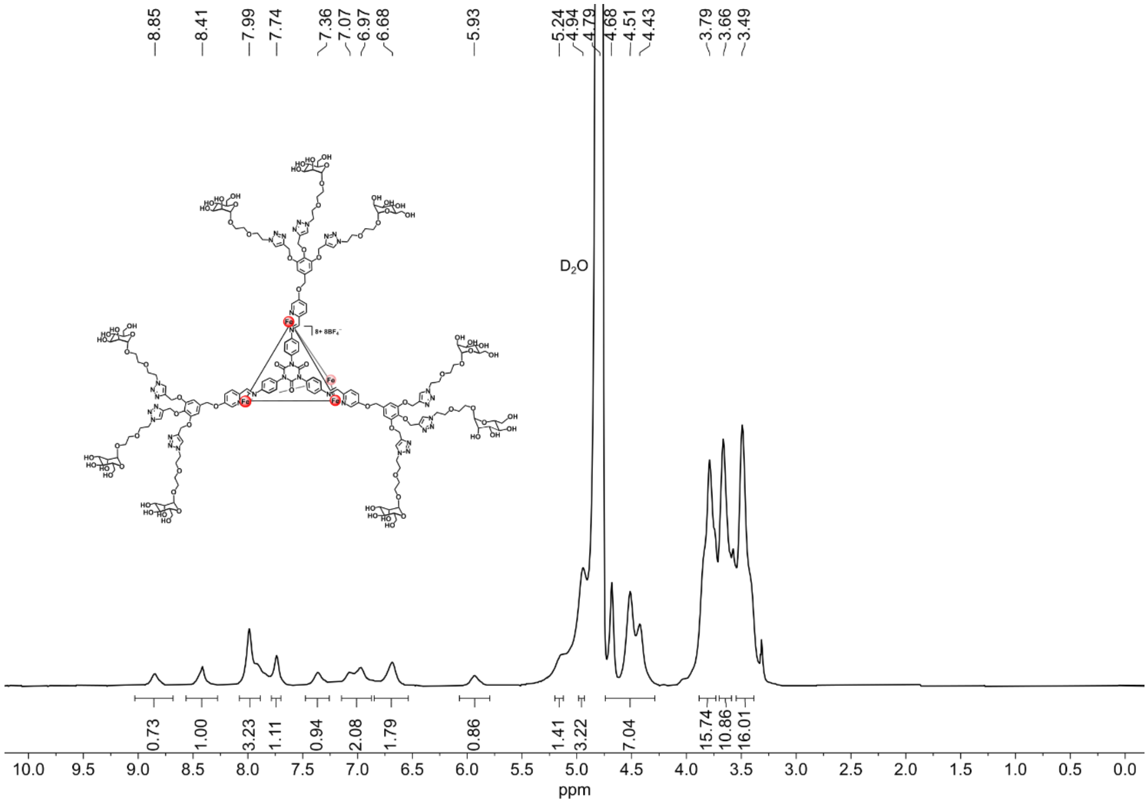

Figure S47. ^{1}H NMR spectrum of [**2**][BF_4]$_8$ (D_2O, 400 MHz, 45 °C).

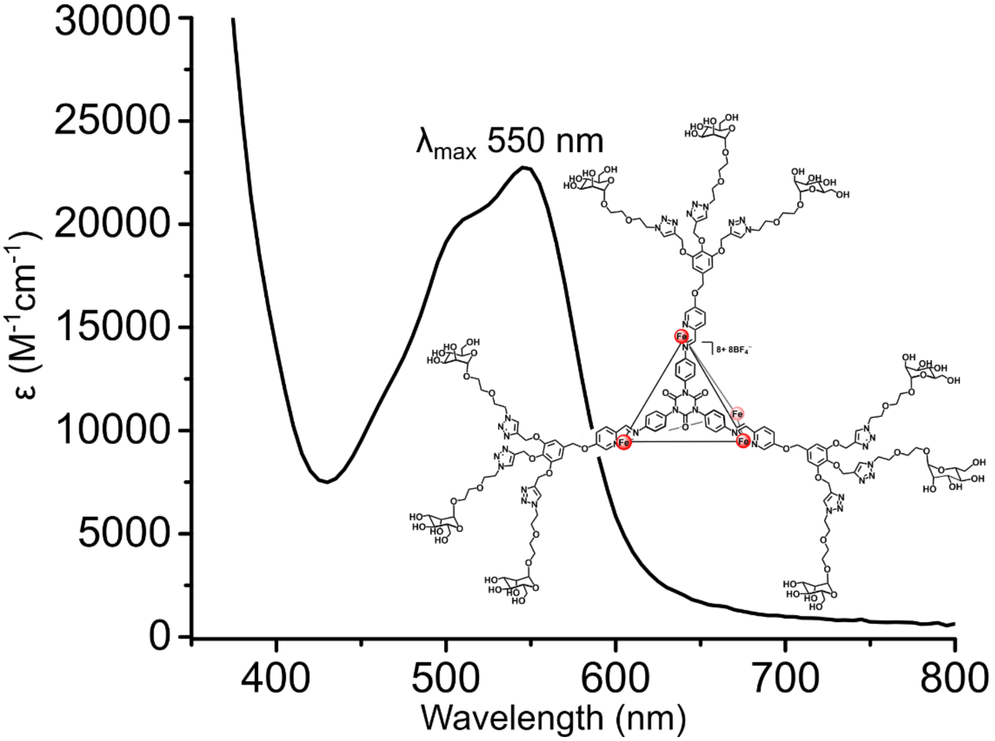

Figure S48. UV-vis spectrum of [**2**][BF_4]$_8$ (H_2O, 50 μM).

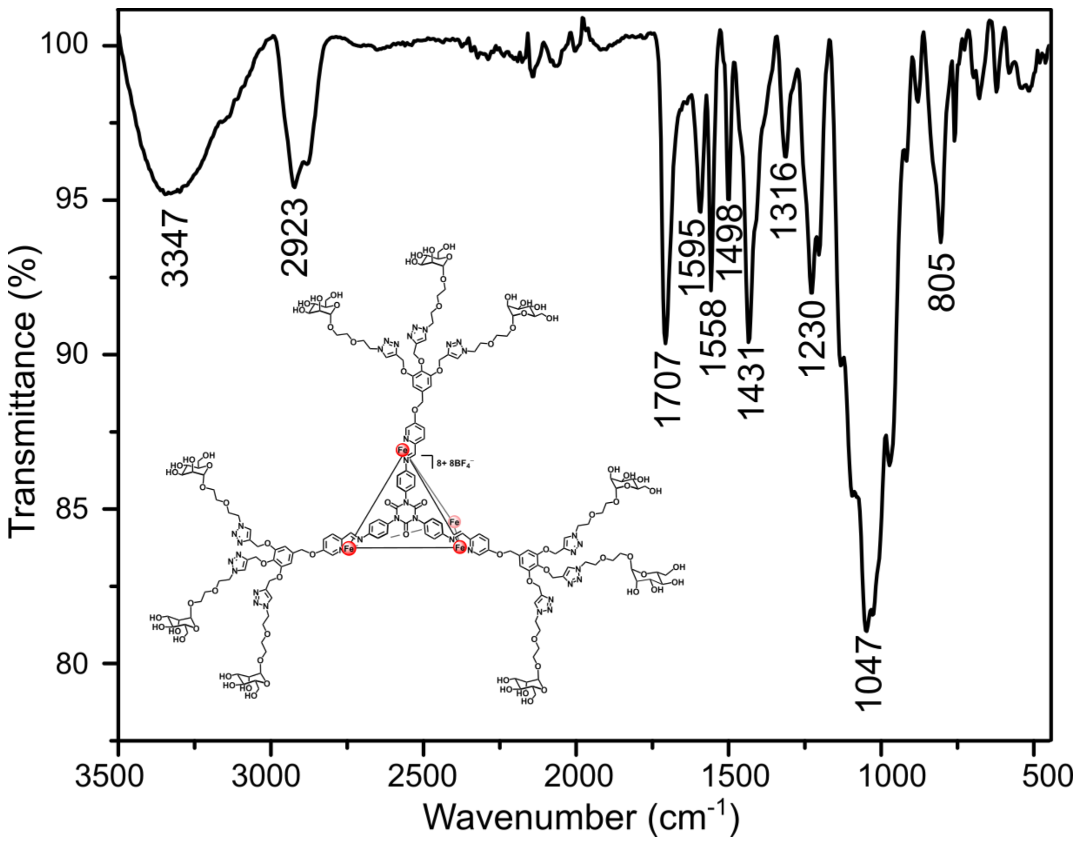

Figure S49. ATR-IR spectrum of [**2**][BF_4]$_8$.

S2.2.3. [3][BF_4]$_8$

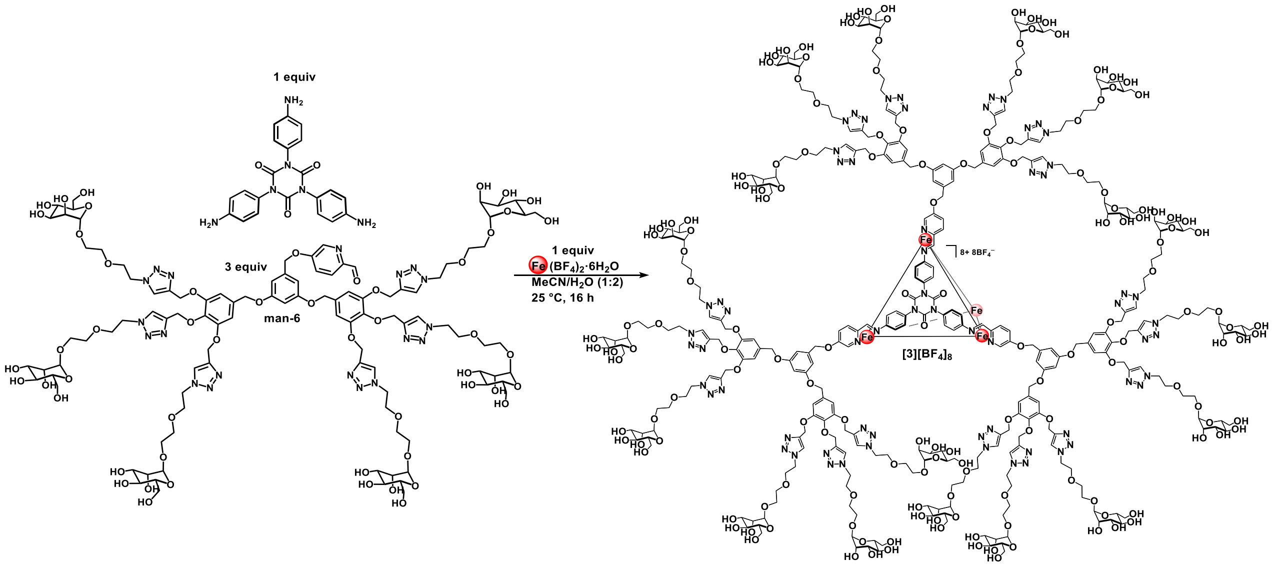

An 8 mL reaction tube was charged with a degassed solution of man-6 (53 mg, 0.021 mmol, 3.0 equiv) in H_2O (1.0 mL). To this solution was added 1,3,5-tris(4-aminophenyl)-1,3,5-triazinane-2,4,6-trione (3 mg, 0.007 mmol, 1 equiv) followed by a degassed solution of MeCN (0.5 mL), and the pale orange solution was allowed to stir at ambient temperature for 10 min under a flow of N_2. To the solution was added $Fe(BF_4)_2$•$6H_2O$ (2 mg, 0.007 mmol, 1 equiv) as a solid, which resulted in an immediate color change to magenta. The reaction mixture was sparged with N_2 for 15 min and was then allowed to stir at 25 °C for 16 h, at which point the magenta solution was filtered through a piece of glass microfiber filter paper and all volatiles were removed under reduced pressure. The pink residue was then dissolved in H_2O (5 mL), and the solution was transferred to the sample reservoir of a Amicon® Ultra Centrifugal Filter (10 kDa MWCO, cellulose membrane). The device was centrifuged at 7500 x g for 75 min, at which point the filtrate in the collection tube was discarded and fresh H_2O (5 mL) was added to the sample reservoir. The device was centrifuged again at 7500 x g for 75 min, and this process was repeated for a total of three centrifuge cycles. After the third cycle, the solution in the sample reservoir was removed and lyophilized overnight to afford the product, [**3**][BF_4]$_8$, as a magenta powder (yield: 15 mg, 0.37 µmol, 26%).^{1}H NMR (400 MHz, 45 °C, D_2O) δ: 8.77 (br s, 1H, *H*C=N), 8.42 (br s, 1H, py-*H*-3), 7.96 (br s, 5H, py-*H*-4, N-C*H* signals overlapping), 7.77 (br s, 4H, *o*-Ar-*H*, N-C*H* signals overlapping), 7.39 (br s, 1H, Ar-*H*), 6.95 (br s, 1H, pyr-*H*-6), 6.69 (br s, 5H, *o*-Ar-*H*, Ar-*H* signals overlapping), 6.42 (br s, 1H, *p*-Ar-*H*), 5.93 (br s, 1H, Ar-*H*), 4.81 (br s, 6H, man-*H*-1), 4.79 (br s, 1H, Ar-*H* overlapping with residual solvent peak), 4.68 (m, 8H, –CH_2–), 4.47-4.39 (m, 16H, man-H-6$_a$, –CH_2– signals overlapping), 3.81-3.73 (m, 32H, man-H-6$_b$, man-*H*-2, –CH_2– signals overlapping), 3.68-3.56 (m, 30H, man-*H*-3, man-*H*-4, man-*H*-5, –CH_2– signals overlapping), 3.77–3.68 (m, 16H, –CH_2–) ppm. UV-vis [ε] (H_2O, 50 µM): λ_{max} 505 [22,000 $M^{-1}cm^{-1}$], 550 [25,000 $M^{-1}cm^{-1}$] nm. ATR-IR (ν): 3347 (OH), 2922, 1707 (C=O), 1595, 1558 (C=N), 1498, 1435, 1316, 1230, 1051, 977, 805 cm^{-1}.

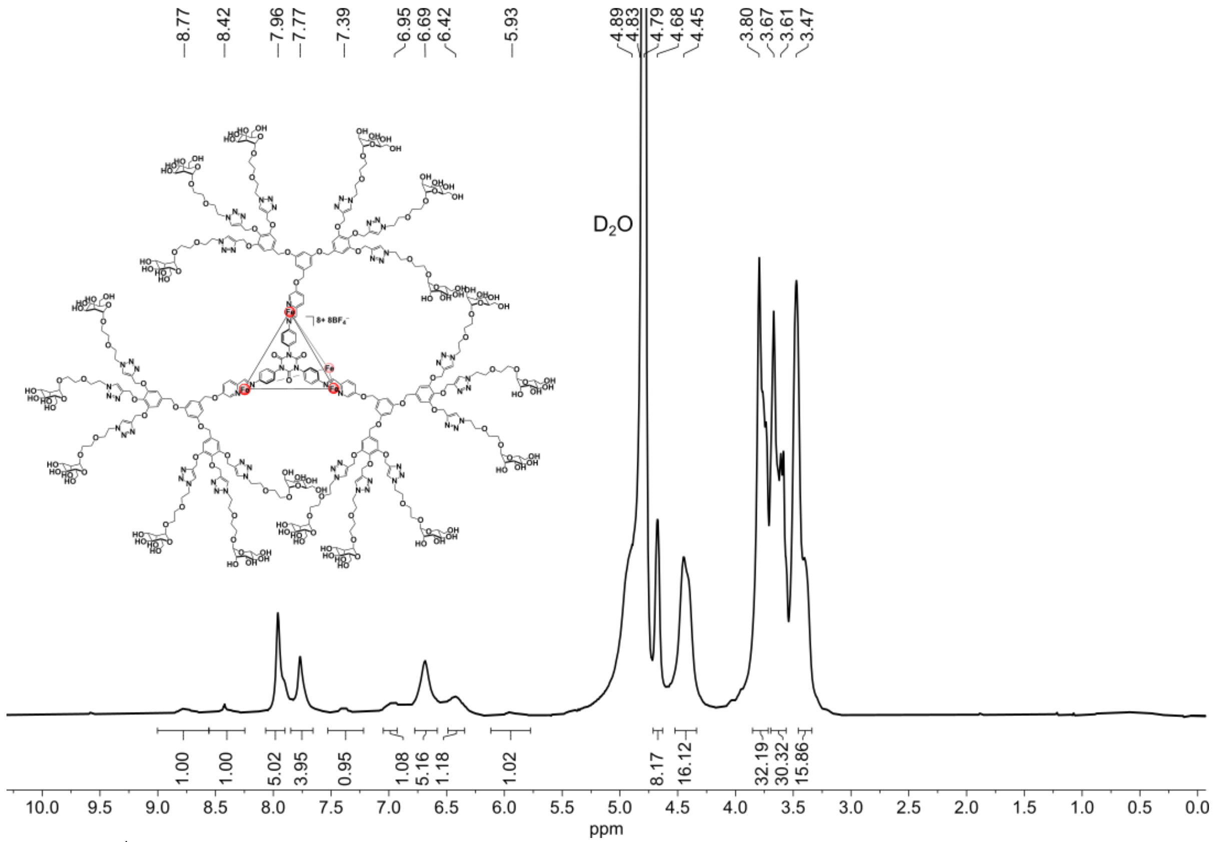

Figure S50. ^{1}H NMR spectrum of [**3**][BF_4]$_8$ (D_2O, 400 MHz, 45 °C).

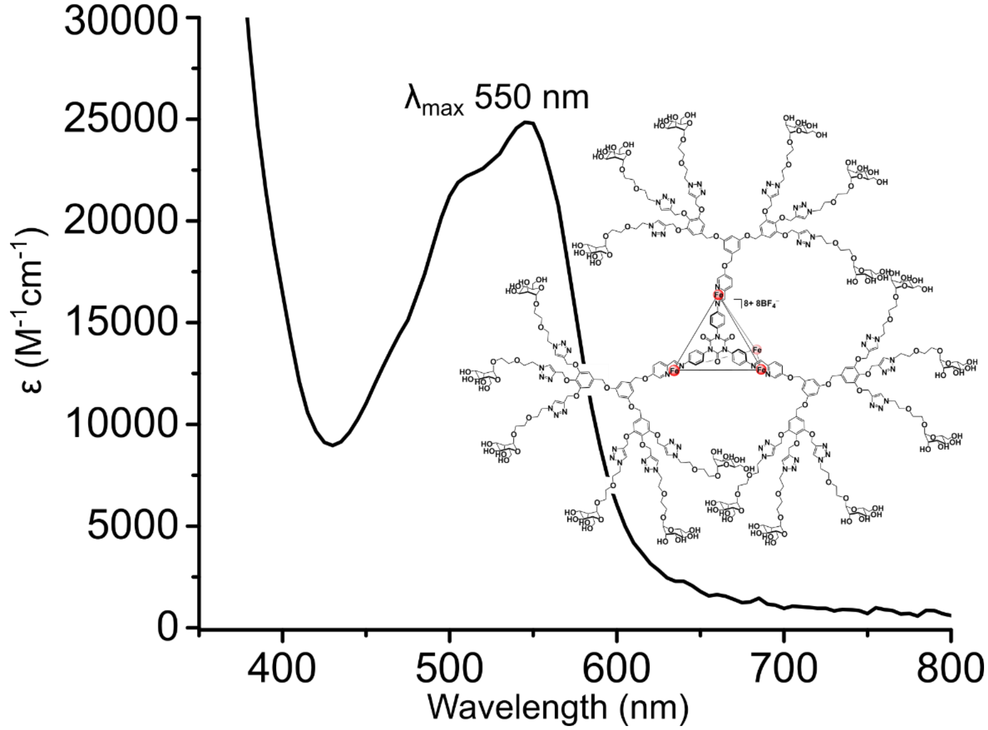

Figure S51. UV-vis spectrum of [**3**][BF_4]$_8$ (H_2O, 50 μM).

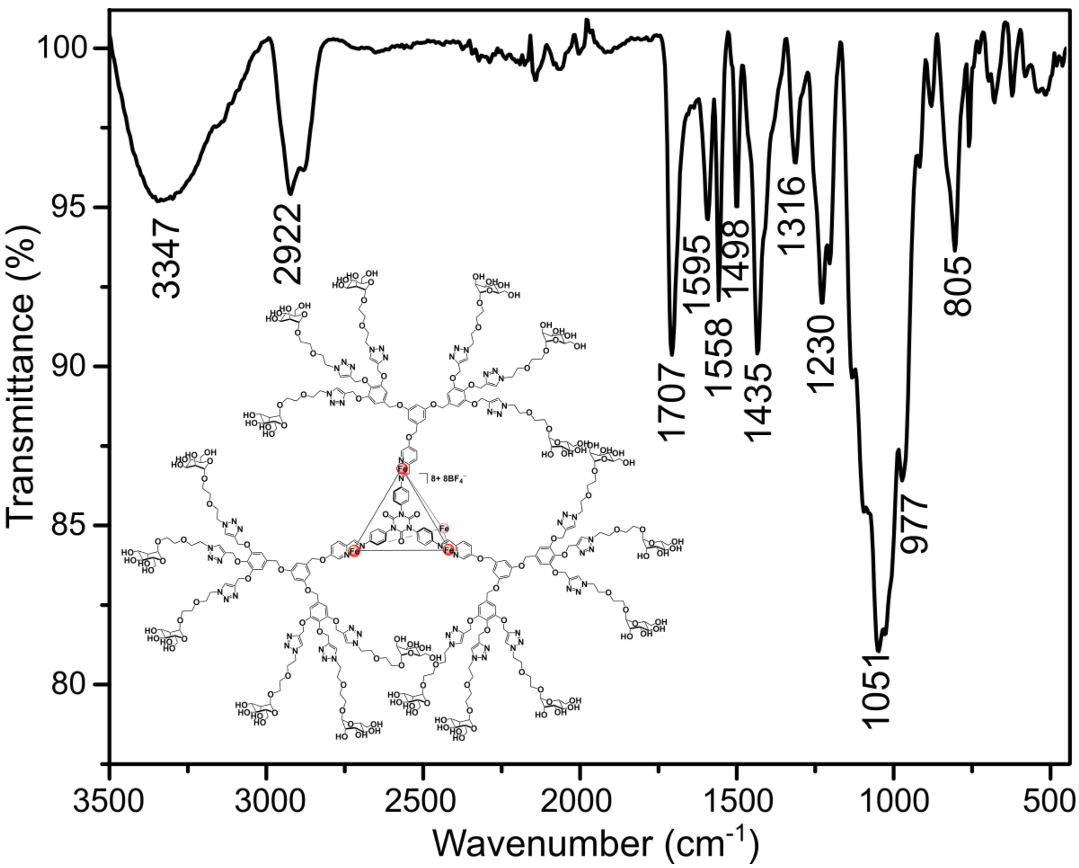

Figure S52. ATR-IR spectrum of [**3**][BF_4]$_8$.

S3. Dynamic Light Scattering (DLS)

All measurements were conducted using a 384-well plate with individual sample volumes of 30 µL. Solutions of man-2, man-3, and man-6 were freshly prepared in H_2O (1 mM). For the glycoassembly analyses, two solution conditions were used: samples prepared in H_2O (5 µM), and samples prepared in mixed DMSO/H_2O solutions (85% v/v, 50 µM). The concentration of each glycoassembly solution was determined by absorption spectroscopy according to ε values ($M^{-1}cm^{-1}$) reported in Section S2.2. All sample solutions were filtered through a 0.22 µm PTFE syringe filter before being added to a well. The sample plate was then centrifuged using a Fisherbrand Mini Plate Spinner Centrifuge at 2500 rpm for 20 sec, and measurements were immediately collected at 25 °C. Before data collection, images were taken of each well to confirm the absence of bubbles or visible dust particles that affect the DLS measurements. The hydrodynamic diameter (D_h) values represent the mean ± standard deviation (σ) of the hydrodynamic size averaged for three (1 sec) scans and all data were analyzed using DYNAMICS (Wyatt) software.

S3.1. Man-2

S3.1.1. Measurement in H_2O (1 mM)

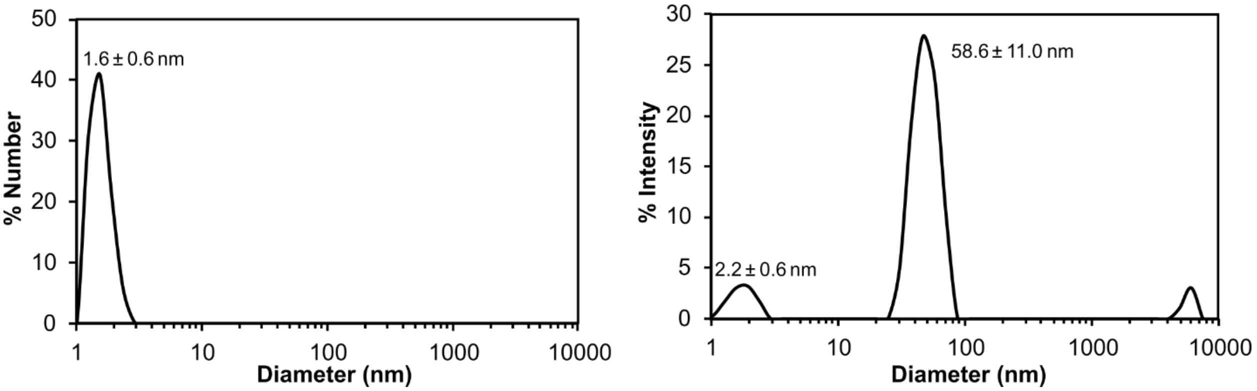

Figure S53. DLS measurements of man-2 (H_2O, 1 mM).

S3.2. Man-3

S3.2.1. Measurement in H_2O (1 mM)

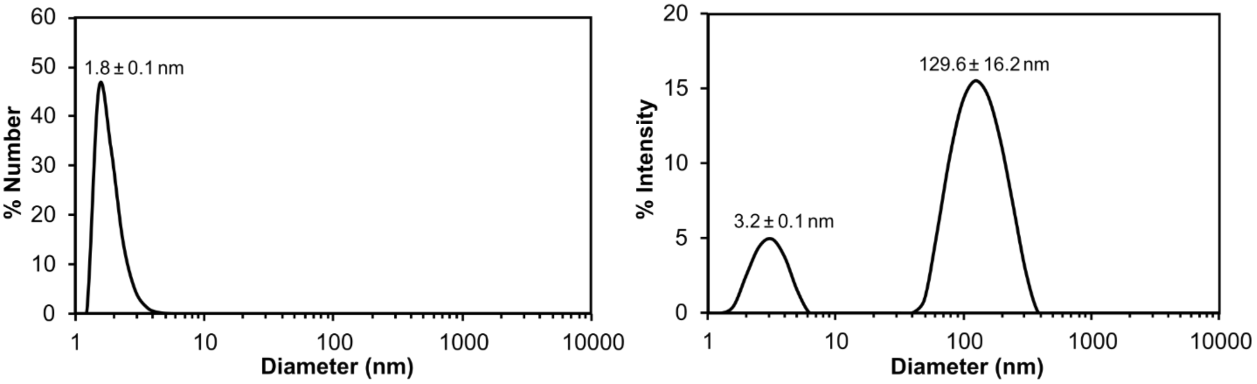

Figure S54. DLS measurements of man-3 (H_2O, 1 mM).

S3.3. Man-6

S3.3.1. Measurement in H_2O (1 mM)

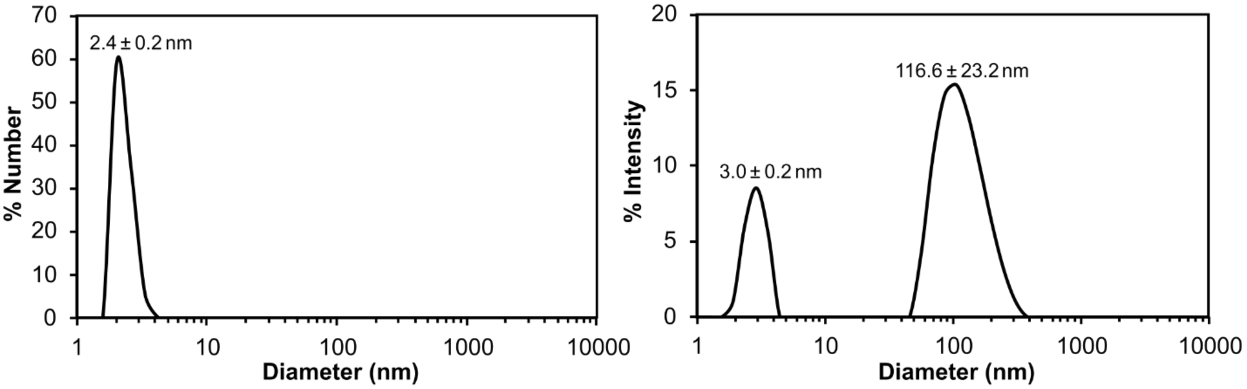

Figure S55. DLS measurements of man-6 (H_2O, 1 mM).

S3.4. [1][BF_4]$_8$

S3.4.1. Measurement in H_2O (5 µM)

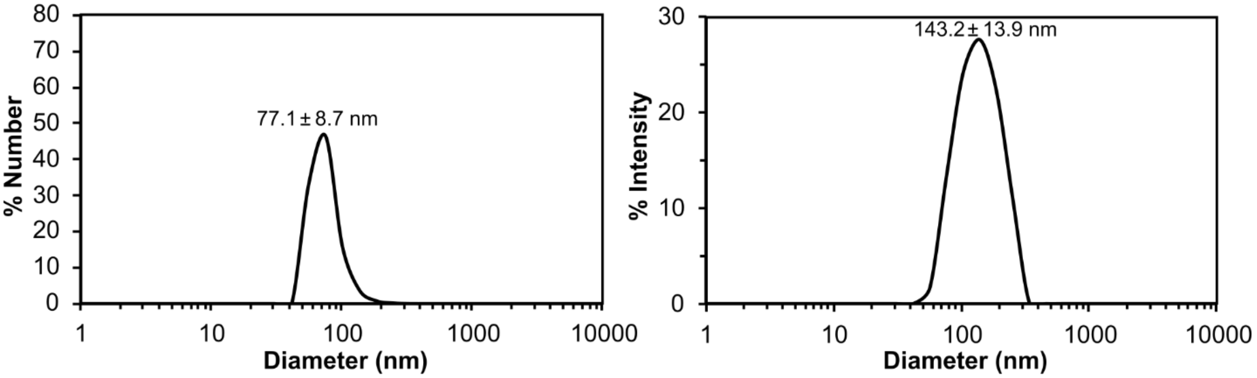

Figure S56. DLS measurements of [**1**][BF_4]$_8$ (H_2O, 5 µM).

S3.4.2. Measurement in DMSO/H_2O (85% v/v, 50 µM)

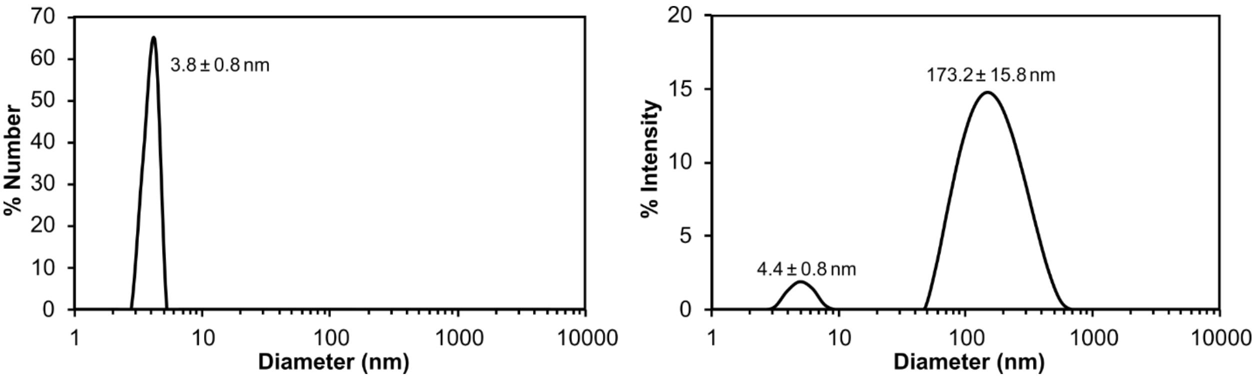

Figure S57. DLS measurements of [**1**][BF_4]$_8$ (DMSO/H_2O 85% v/v, 50 µM).

S3.5. [2][BF_4]$_8$

S3.5.1. Measurement in H_2O (5 µM)

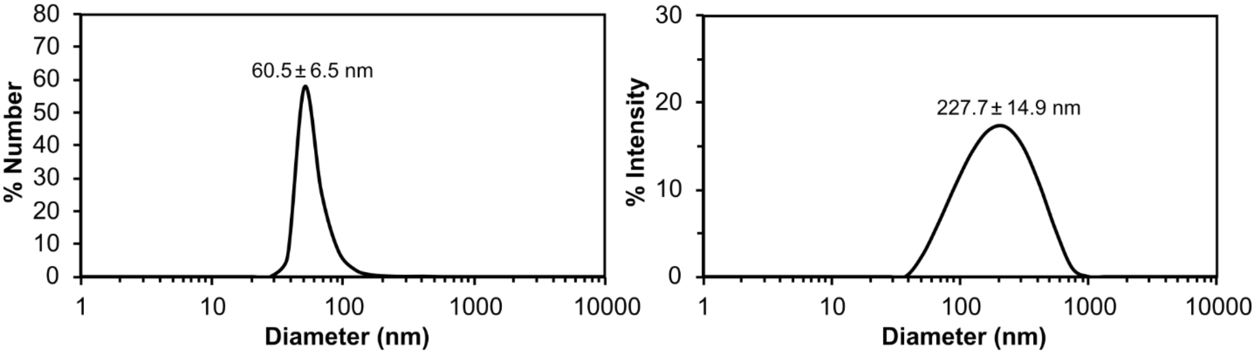

Figure S58. DLS measurements of [**2**][BF_4]$_8$ (H_2O, 50 µM).

S3.5.2. Measurement in DMSO/H_2O (85% v/v, 50 µM)

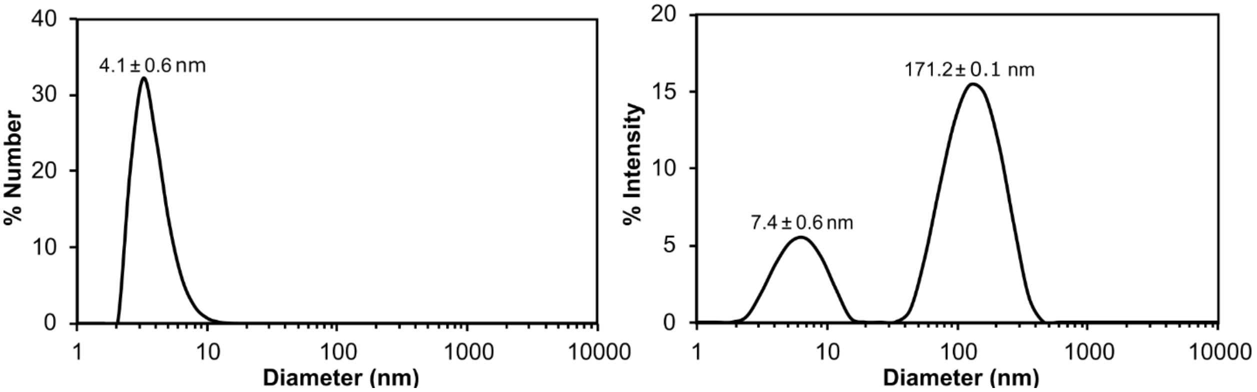

Figure S59. DLS measurements of [**2**][BF_4]$_8$ (DMSO/H_2O 85% v/v, 50 µM).

S3.6. [3][BF_4]$_8$

S3.6.1. Measurement in H_2O (5 µM)

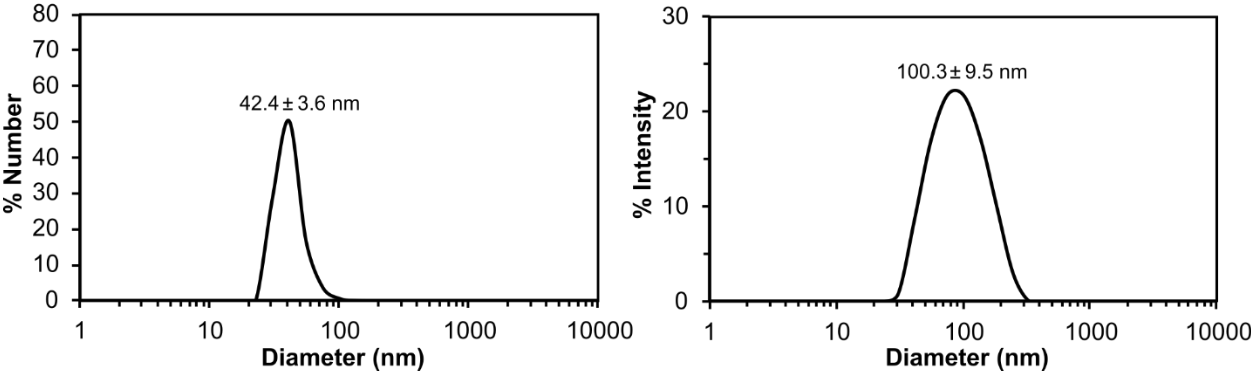

Figure S60. DLS measurements of [**3**][BF_4]$_8$ (H_2O, 5 µM).

S3.6.2. Measurement in DMSO/H_2O (85% v/v, 50 µM)

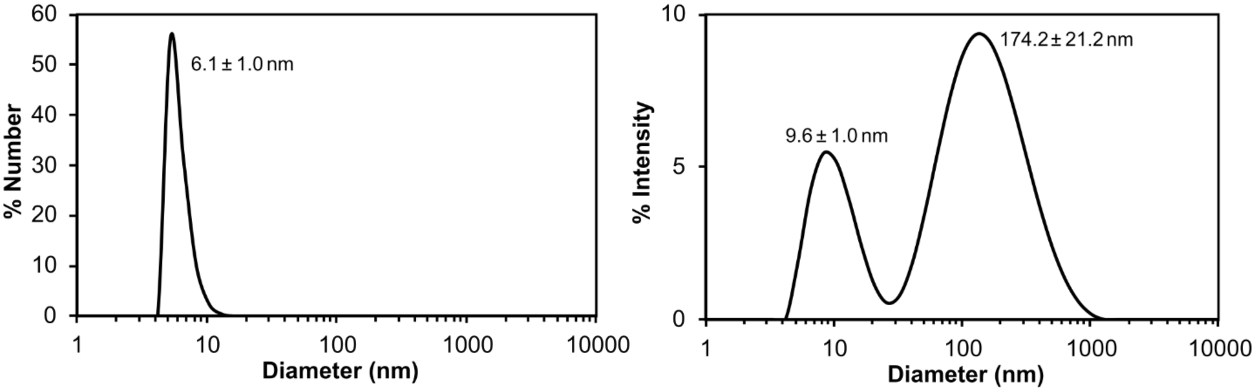

Figure S61. DLS measurements of [**3**][BF_4]$_8$ (DMSO/H_2O 85% v/v, 50 µM).

S4. Transmission Electron Microscopy (TEM)

A PELCO easiGlow system was used to increase the hydrophilicity of the carbon-coated copper grids (400-mesh, Electron Microscopy Sciences) before loading the samples. Aqueous solutions of the samples (5 μL of 0.2 μM solutions) were prepared and then applied to the grids, which were allowed to stand for 5 min. After excess solution was removed, the grids were washed with H_2O and allowed to dry for 20 min. The grids were then negatively stained by applying a 2% solution of uranyl acetate in H_2O. The staining solution was removed after 1 min and the grid was allowed to dry for at least 24 h. TEM micrographs were imaged at 80 kV with a JEOL 1400 Plus transmission electron microscope equipped with a bottom-mount Gatan OneView (4k x 4k) camera. Data were processed with Fiji-ImageJ v1.54f software.

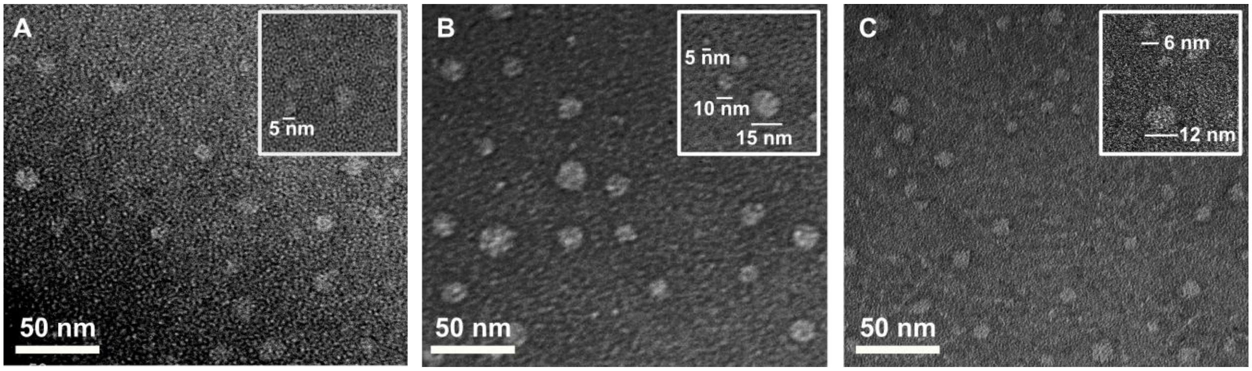

Figure S62. Negative-contrast electron micrographs (uranyl acetate) of A) [**1**]$[BF_4]_8$, B) [**2**]$[BF_4]_8$, and C) [**3**]$[BF_4]_8$.

S5. Atomic Force Microscopy (AFM)

AFM samples were prepared by drop casting glycoassembly solutions (2 µL of 0.2 µM solution in H_2O) onto freshly cleaved mica (Ted Pella). The H_2O was then removed under reduced pressure in a vacuum desiccator. Images were scanned with a tapping amplitude in the range of 15-25 nm and processed using Gwyddion v2.67 software.

S5.1. [1][BF_4]$_8$

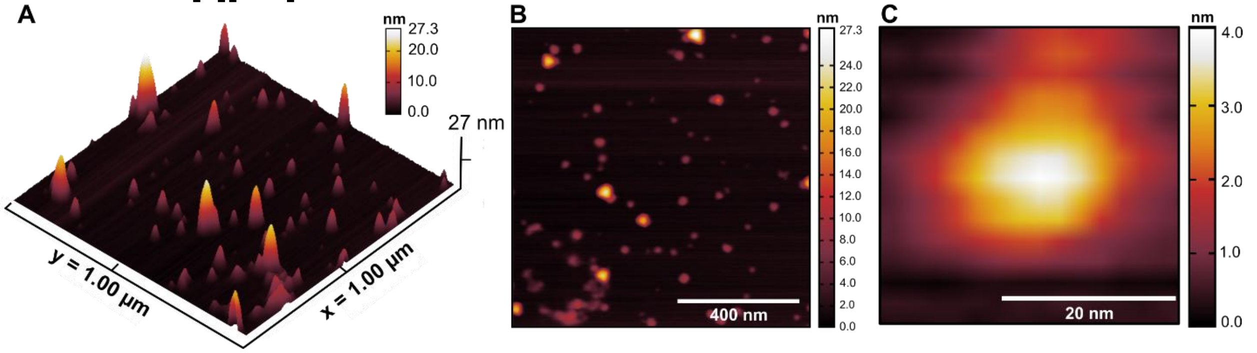

Figure S63. AFM images and height profiles of [**1**][BF_4]$_8$ prepared in H_2O (0.2 µM). The brightness of the AFM height images corresponds to a vertical range of 27.3 nm (A, B), and 4.0 nm (C).

S5.2. [2][BF_4]$_8$

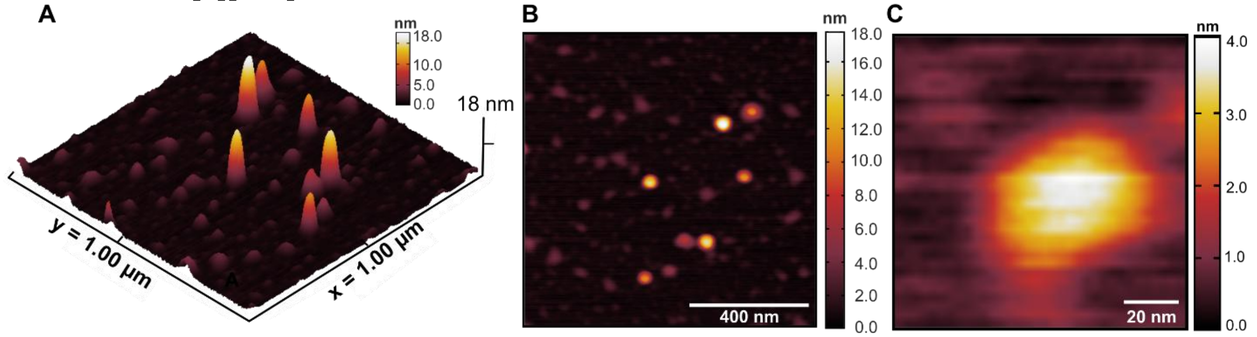

Figure S64. AFM images and height profiles of [**2**][BF_4]$_8$ prepared in H_2O (0.2 µM). The brightness of the AFM height images corresponds to a vertical range of 18.0 nm (A, B), and 4.0 nm (C).

S5.3. [3][BF_4]$_8$

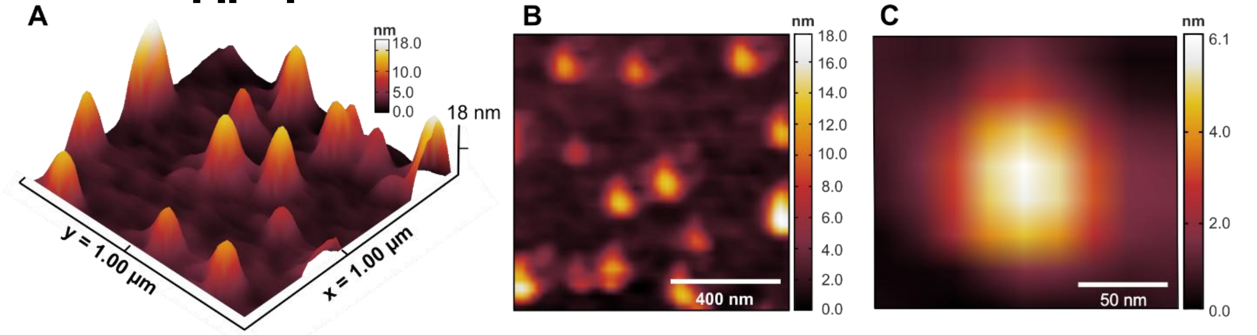

Figure S65. AFM images and height profiles of [**3**][BF_4]$_8$ prepared in H_2O (0.2 µM). The brightness of the AFM height images corresponds to a vertical range of 18.0 nm (A, B), and 6.1 nm (C).

S6. Stability Studies of [3][BF_4]$_8$ to various buffers, and pH values.

General procedure for all stability studies: A stock solution of [**3**][BF_4]$_8$ (1 mM) was prepared in DI H_2O. An aliquot (50 µL) was diluted in the following solutions to 50 µM concentration to reach a total volume of 1 mL, and an initial UV-vis spectrum was collected. The solutions were allowed to stand at the indicated temperature for up to 24 h, and UV-vis spectra were collected at the indicated time-points.

S6.2. DMEM cell medium, 10% fetal bovine serum at 37 °C

This experiment was carried out in DMEM cell medium 10% FBS at 37 °C.

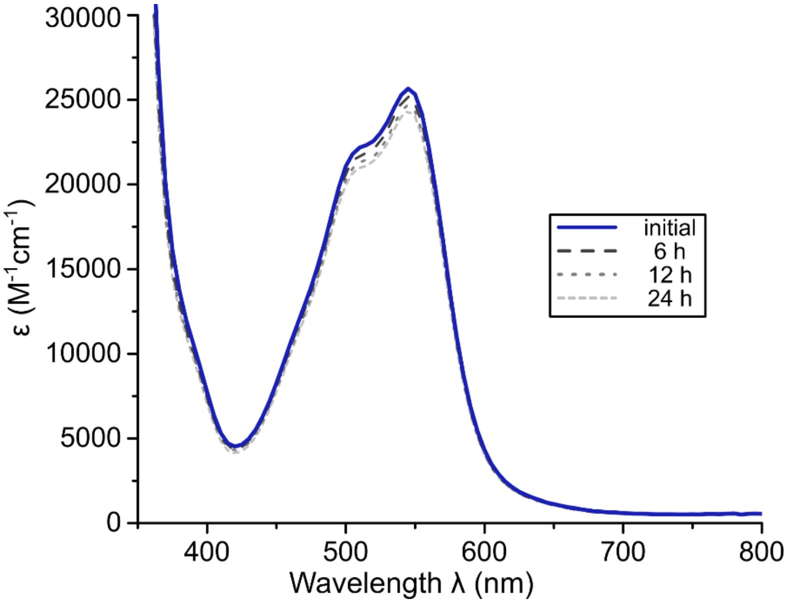

Figure S66. UV-vis spectra of [**3**][BF_4]$_8$ collected in DMEM 10% FBS over the course of 24 h at 37 °C.

S6.3. Carbonate buffer, pH 10

This experiment was carried out in carbonate buffer ($NaHCO_3$/Na_2CO_3, 10 mM) at pH 10.0 and at 25 °C.

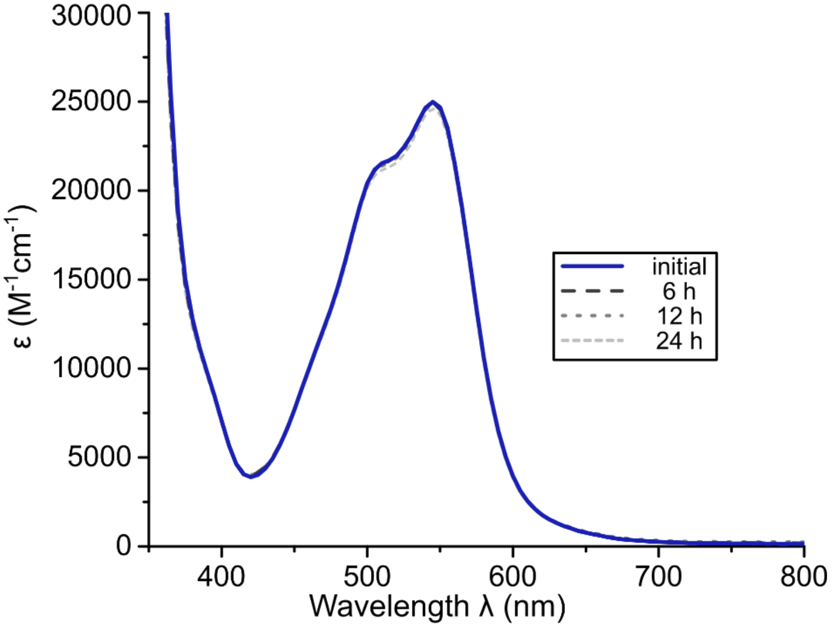

Figure S67. UV-vis spectra of [**3**][BF_4]$_8$ collected in carbonate buffer (10 mM) at pH 10.0 over the course of 24 h at 25 °C.

S6.4. PBS buffer, pH 7.4

This experiment was carried out in PBS buffer (10 mM) at pH 7.4 and at 25 °C.

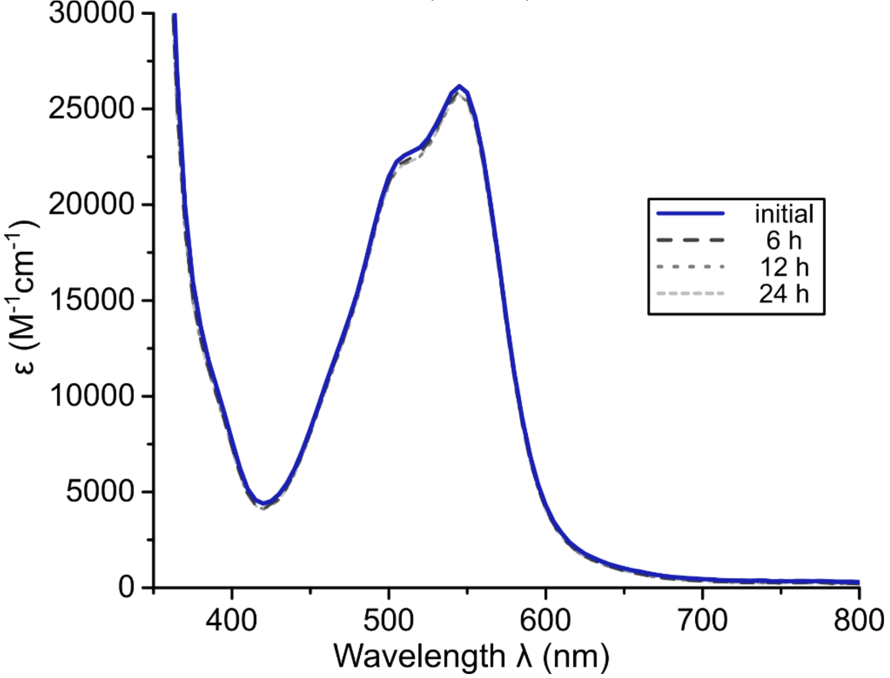

Figure S68. UV-vis spectra of [**3**][BF_4]$_8$ collected in PBS buffer (10 mM) at pH 7.4 over the course of 24 h at 25 °C.

S6.5. Tris buffer, pH 7.4

This experiment was carried out in Tris buffer (10 mM) at pH 7.4 and at 25 °C.

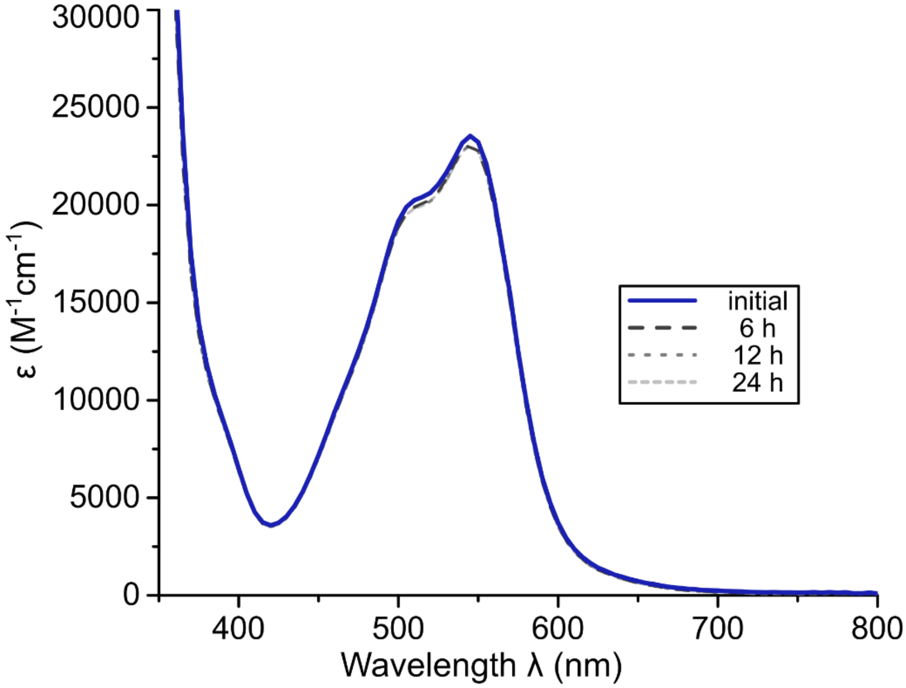

Figure S69. UV-vis spectra of [**3**][BF_4]$_8$ collected in Tris buffer (10 mM) at pH 7.4 over the course of 24 h at 25 °C.

S6.6. Acetate buffer, pH 5.0

This experiment was carried out in acetate buffer (10 mM) at pH 5.0 and at 25 °C.

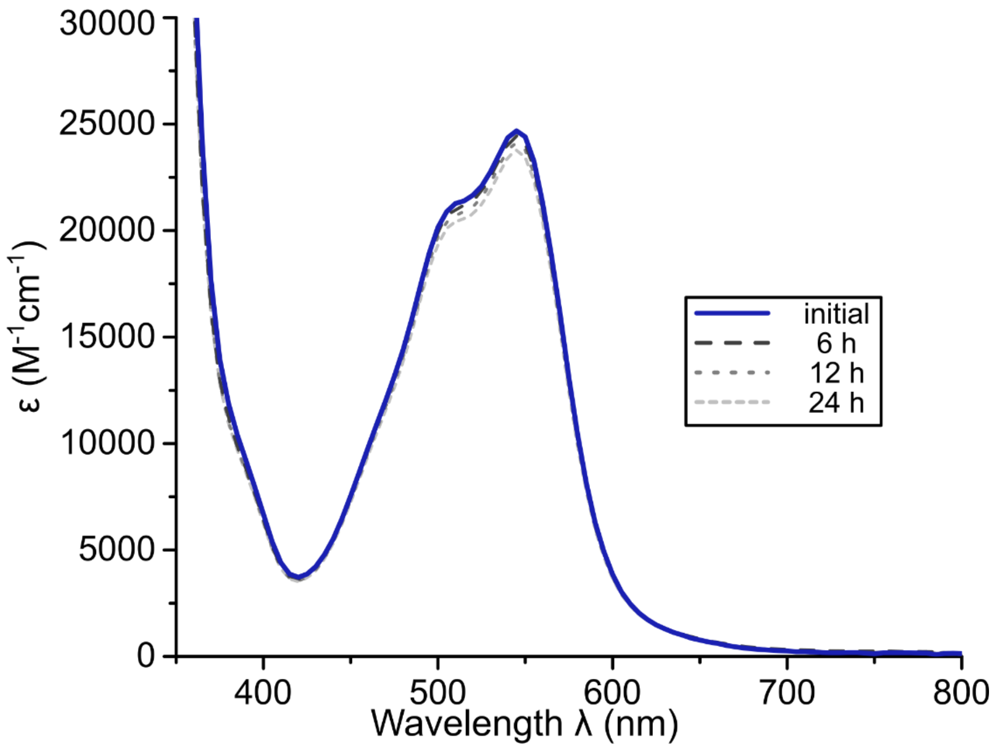

Figure S70. UV-vis spectra of [**3**][BF_4]$_8$ collected in acetate buffer (10 mM) at pH 5.0 over the course of 24 h at 25 °C.

S7. ITC binding studies

All ITC experiments were performed using a VP-ITC (Microcal Inc.) instrument. Injections of solutions containing the glycoassembly dissolved in the same buffer as the protein were added from a computer controlled 300 µL syringe at an interval of 280 sec into the sample solution of protein (cell volume 1.459 mL) with a stirring rate of 307 rpm. The first of 26 total injections was performed at 2.0 µL volume and was discarded from all data sets in order to remove the effect of titrant diffusion across the syringe tip during the equilibration process. The following 25 injections were performed at 10.0 µL volume. The glycoassembly concentrations were determined by UV-vis spectroscopy by measuring the absorbance at $\lambda_{550\ nm}$ (ε values reported for all complexes in Section S2.2). To eliminate any unspecific enthalpic contributions (heat of dilution), control experiments were performed by titration of the glycoassembly into the buffer solution in the absence of protein. The data obtained for the glycoassembly dilution blank were subtracted from the data obtained for glycoassembly/protein titration experiments. The experimental data were fitted to a one-site binding model using OriginPro7 software v7.0383, with ΔH (enthalpy change in kJ/mol), K_d (dissociation constant in M), and n (stoichiometry of binding) as adjustable parameters. Thermodynamic parameters were calculated according to the Gibbs−Helmholtz equation, and all data sets were collected in triplicate.

S7.1. Concanavalin A (Con A)

Titrations were conducted in an acetate buffer (100 mM) adjusted to pH 4.8 to generate Con A in its dimeric form and in the presence of $MnCl_2$ (0.1 mM), $CaCl_2$ (0.1 mM), and NaCl (10 mM). The buffer solution was prepared in Milli-Q water and filtered through a Corning® 0.22 µm filter prior to use. The concentration of Con A used for all titrations (80-82 µM) was recorded with respect to the monomer (M_r = 25,600 kDa), and glycoassembly concentrations ranged from 3-115 µM. The exact concentration of Con A was determined by UV-vis spectroscopy by measuring the absorbance at λ 280 nm [$E_{1\mathrm{cm}}^{1\%}$ = 12.4].[13]

S7.1.1. [1][BF_4]$_8$

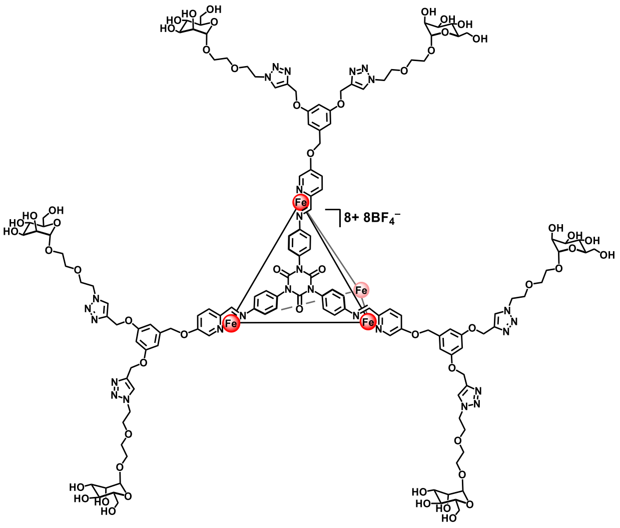

Measurements were conducted with solutions of Con A (82 μM with respect to monomer) and [**1**][BF_4]$_8$ (115 μM).

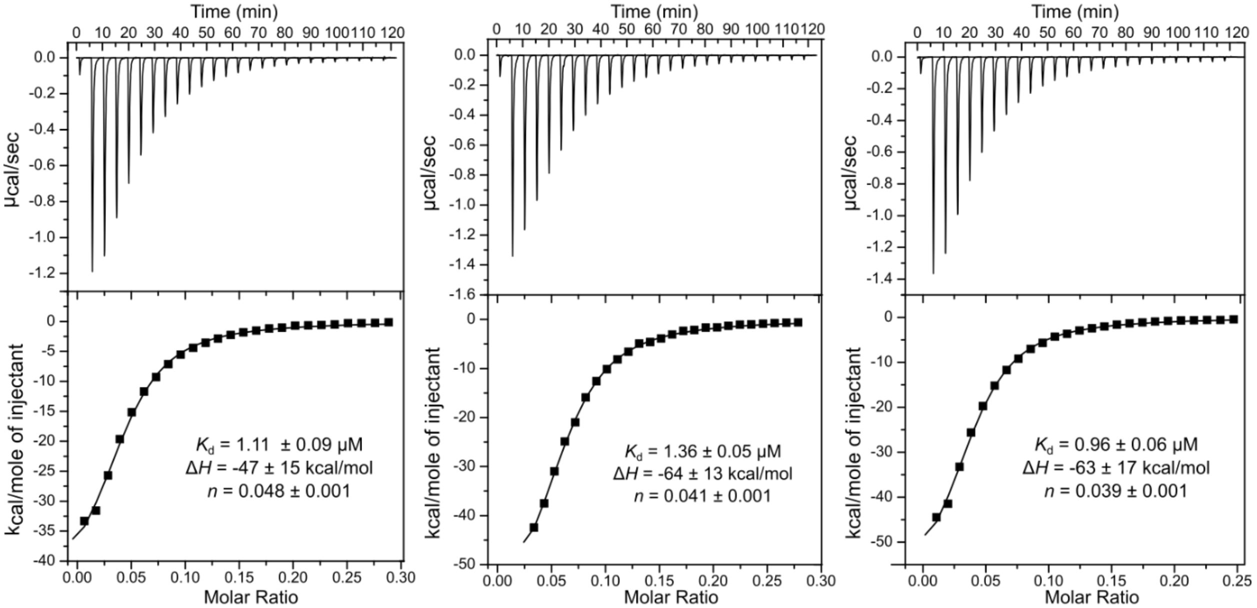

Figure S71. Isothermal titration calorimetry thermograms (top) and fitted binding isotherms (bottom) for the binding of [**1**][BF_4]$_8$ to dimeric Con A for three independent runs. Values of n are reported with respect to the Con A monomer.

S7.1.2. [2][BF$_4$]$_8$

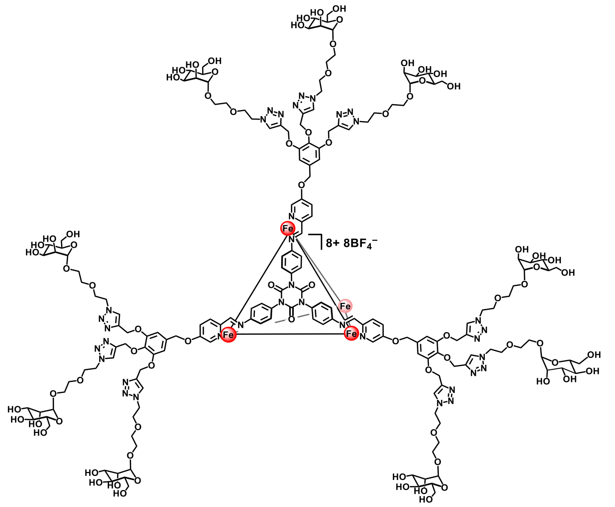

Measurements were conducted with solutions of Con A (80 µM with respect to monomer) and [**2**][BF$_4$]$_8$ (38 µM).

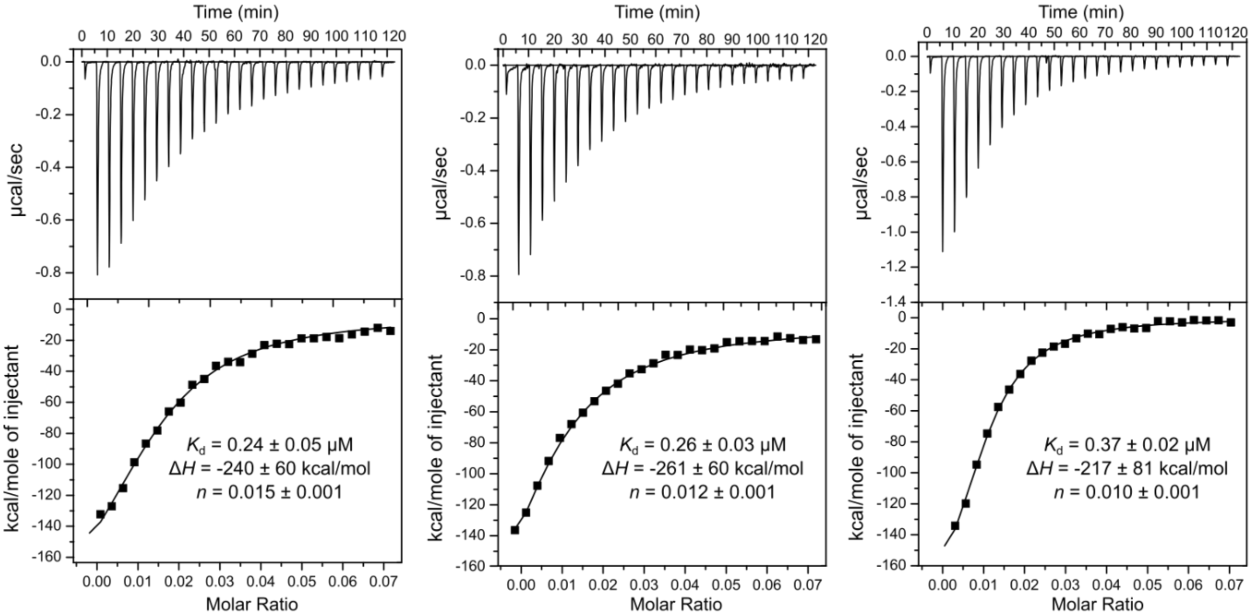

Figure S72. Isothermal titration calorimetry thermograms (top) and fitted binding isotherms (bottom) for the binding of [**2**][BF$_4$]$_8$ to dimeric Con A for three independent runs. Values of *t* are reported with respect to the Con A monomer.

S7.1.3. [3][BF$_4$]$_8$

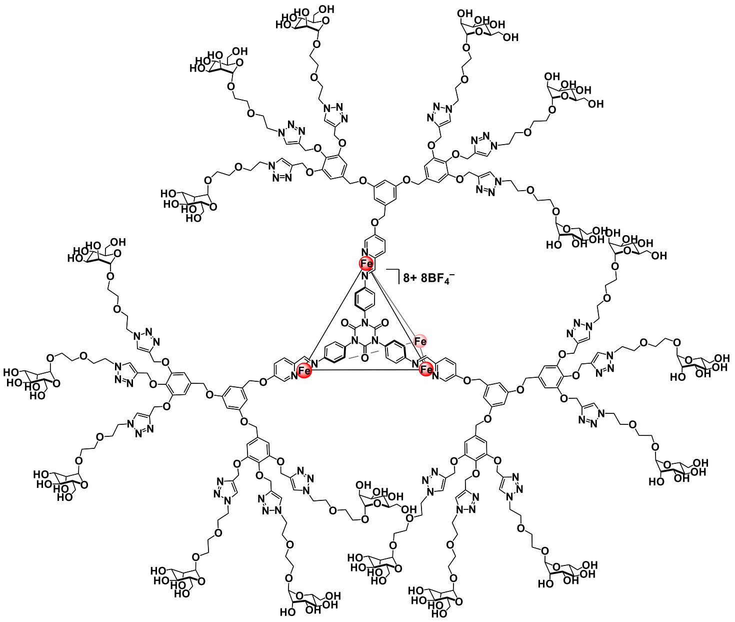

Measurements were conducted with solutions of Con A (82 μM with respect to monomer) and [**3**][BF$_4$]$_8$ (3 μM).

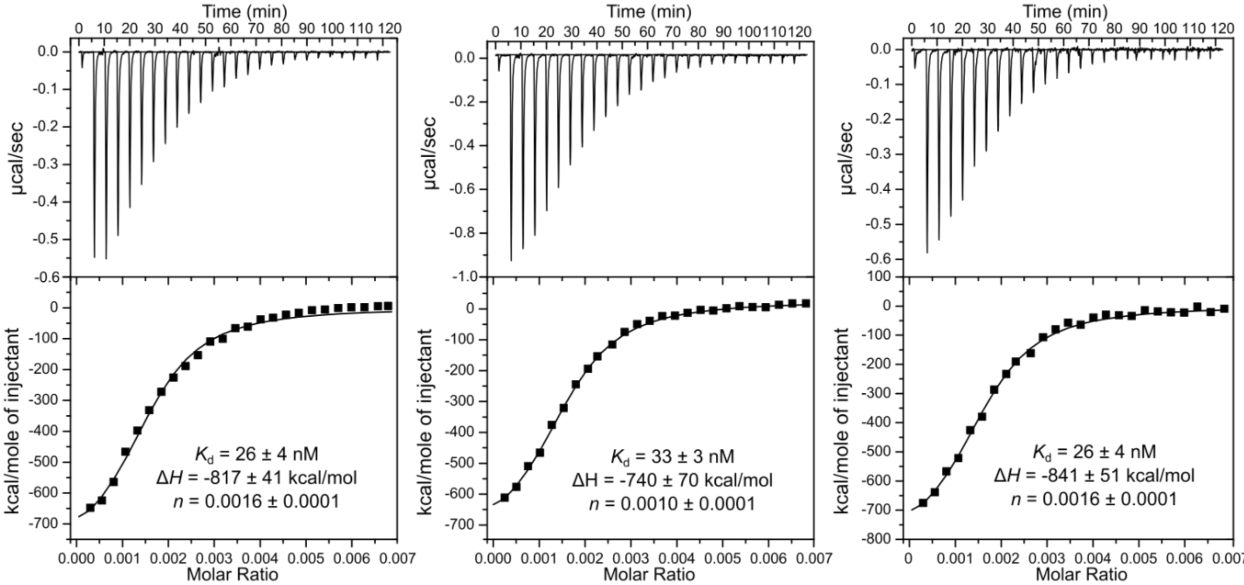

Figure S73. Isothermal titration calorimetry thermograms (top) and fitted binding isotherms (bottom) for the binding of [**3**][BF$_4$]$_8$ to dimeric Con A for three independent runs. Values of *n* are reported with respect to the Con A monomer.

S7.2. Griffithsin (GRFT)

Titrations were conducted in PBS buffer (10 mM) at pH 7.4 with the concentration of monomeric GRFT at 40 µM. The buffer solution was prepared in Milli-Q water and filtered through a Corning® 0.22 µm filter prior to use. Solutions of glycoassemblies were prepared in the same buffer at concentrations ranging from 150-20 µM

S7.2.1. [Fe_4L_4-12][BF_4]$_8$

Measurements were conducted with solutions of [**Fe_4L_4-12**][BF_4]$_8$[1] at 150 µM.

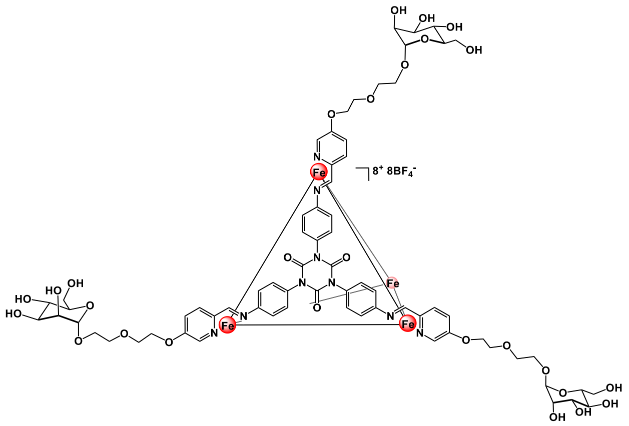

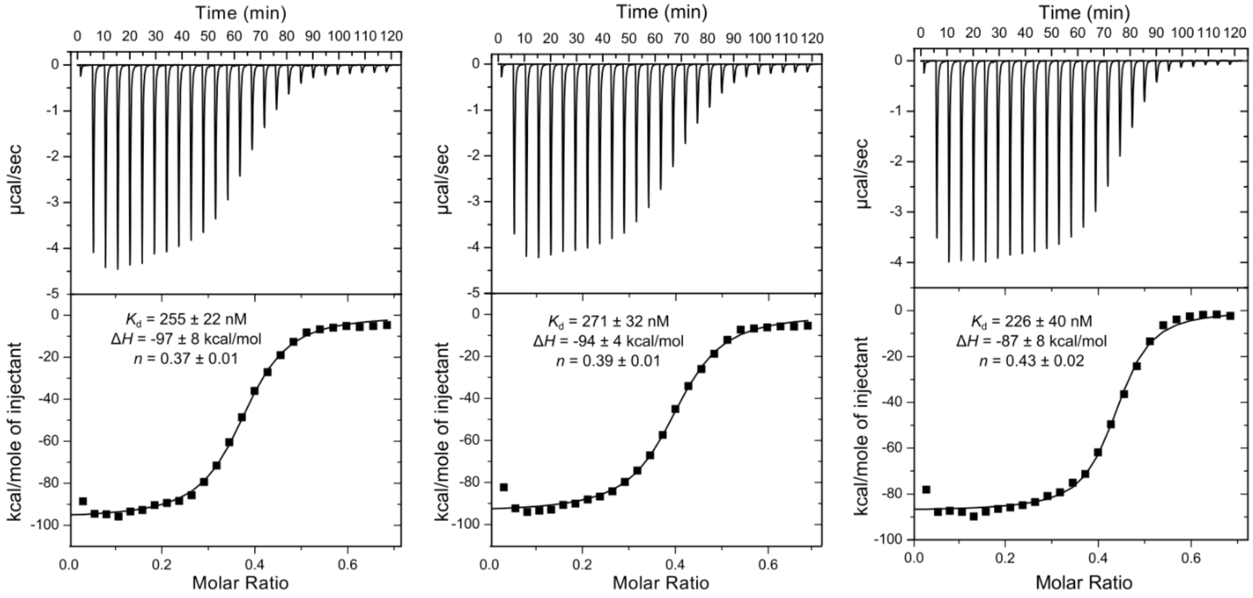

Figure S74. Isothermal titration calorimetry thermograms (top) and fitted binding isotherms (bottom) for the binding of [**Fe_4L_4-12**][BF_4]$_8$ to GRFT for three independent runs.

S7.2.2. [1][BF_4]$_8$

Measurements were conducted with solutions of [**1**][BF_4]$_8$ at 75 μM.

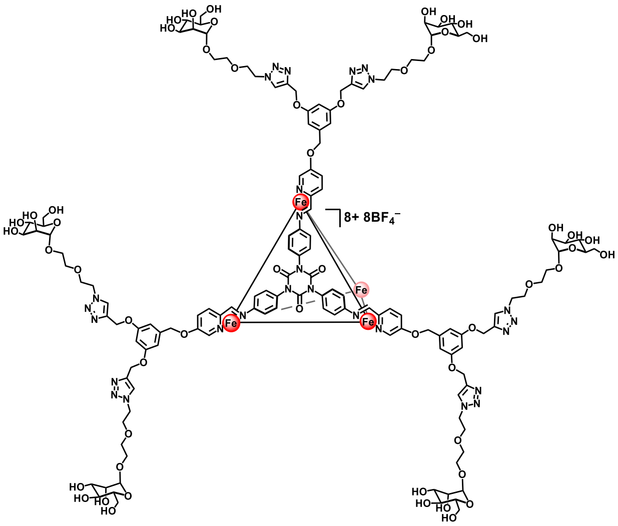

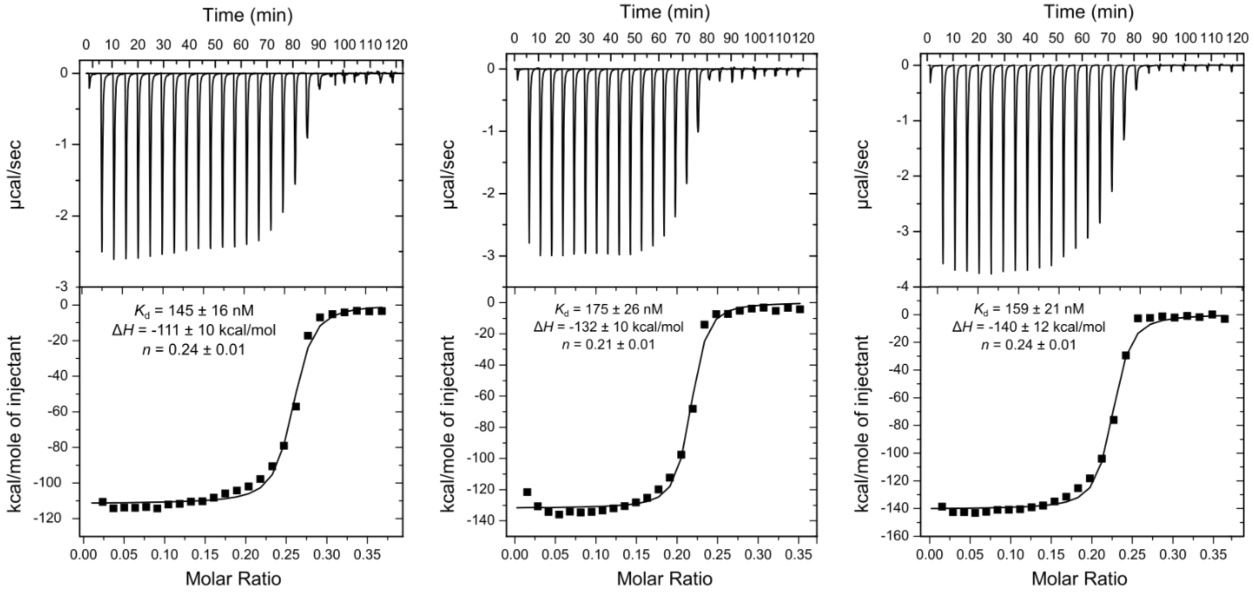

Figure S75. Isothermal titration calorimetry thermograms (top) and fitted binding isotherms (bottom) for the binding of [**1**][BF_4]$_8$ to GRFT for three independent runs.

S7.2.3. [2][BF$_4$]$_8$

Measurements were conducted with solutions of [**2**][BF$_4$]$_8$ at 38 μM.

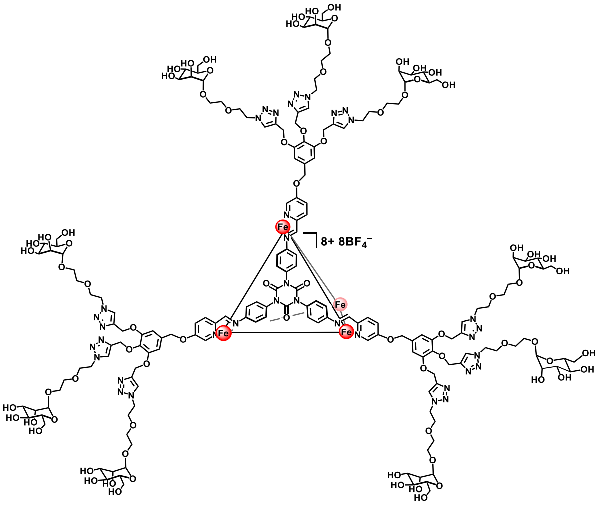

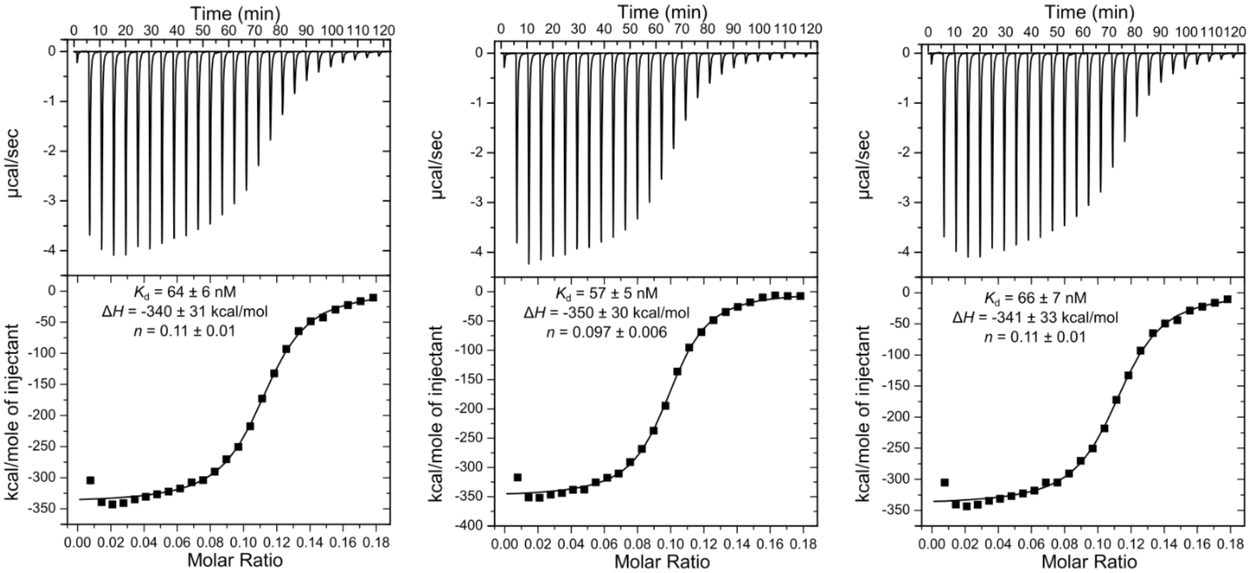

Figure S76. Isothermal titration calorimetry thermograms (top) and fitted binding isotherms (bottom) for the binding of [**2**][BF$_4$]$_8$ to GRFT for three independent runs.

S7.2.4. [3][BF_4]$_8$

Measurements were conducted with solutions of [**3**][BF_4]$_8$ at 20 μM.

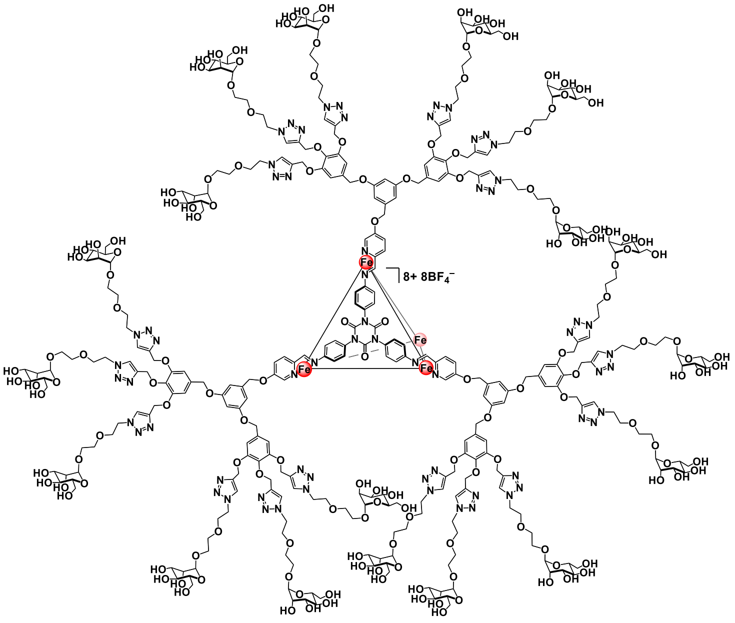

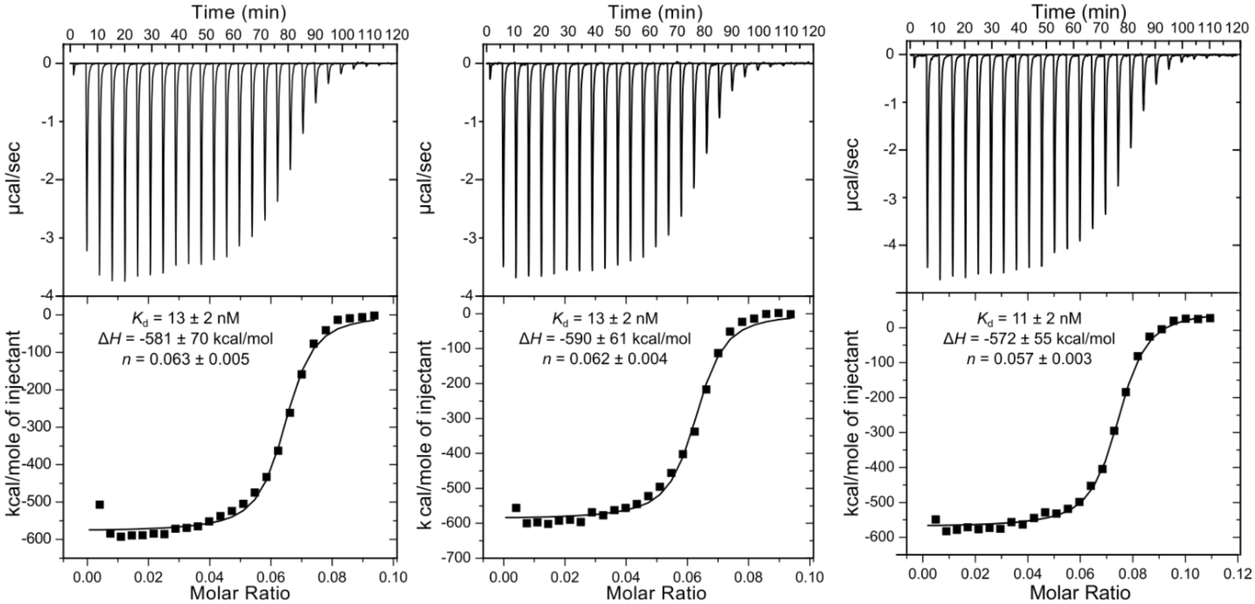

Figure S77. Isothermal titration calorimetry thermograms (top) and fitted binding isotherms (bottom) for the binding of [**3**][BF_4]$_8$ to GRFT for three independent runs.

S8. Griffithsin Protein Expression and Purification

Escherichia coli BL21(DE3) were transformed with a pET28b plasmid containing a N-terminal hexa-his tag followed by a TEV cleavage site and the sequence for monomeric Grifithsin.[14] Single colonies were shaken overnight at 37 °C in Luria-Bertani (LB) broth containing antibiotic. Overnight cultures were diluted 1:50 in LB with antibiotic and shaken at 37 °C until an optical density at 600 nm of 0.6 was reached. Protein expression was induced with isopropyl β-D-1-thiogalactopyranoside (IPTG, 1 mM) and cells were incubated at 18 °C for 16 h. Cells were collected *via* centrifugation at 4,000 x g for 20 min and the pellet was re-suspended in PBS (40 mL of a 10 mM solution) with NaCl (200 mM) and imidazole (50 mM) at pH 7.4. Resuspended pellets were stored at -80 °C until purification.

The cells were thawed and then lysed using sonication and clarified *via* centrifugation at 20,000 x g for 25 min. The supernatant was filtered through a 0.45 μM filter before loading onto a 5 mL Cytiva® HisTrap HP column on a GE AKTA FPLC. The column was washed with 5 CV (10 mM PBS, 200 mM NaCl, 20 mM imidazole at pH 7.4), then eluted using an imidazole gradient from 0 to 100% (10 mM PBS, 200 mM NaCl, 500 mM imidazole at pH 7.4). Fractions were collected and dialyzed against PBS (10 mM) overnight at 4 °C. The sample was diluted 1:2 with Tris buffer (20 mM, pH 7.5), and loaded onto a Cytiva® HiTrap Q anion exchange column. The flow-through was collected and dialyzed against PBS (10 mM). The protein was concentrated using a Sartorius® 10K MWCO concentrator. The purity and identity of mGRFT were assessed *via* SDS-PAGE.

S9. References

1 J. M. Stauber, *Angew. Chemie Int. Ed.*, 2024, **63**, e202408751.

2 J. H. Schwab, J. B. Bailey, M. Gembicky and J. M. Stauber, *Chem. Sci.*, 2023, **14**, 1018–1026.

3 K. Babiuch, A. Dag, J. Zhao, H. Lu and M. H. Stenzel, *Biomacromolecules*, 2015, **16**, 1948–1957.

4 A. Palmioli and B. La Ferla, *Org. Lett.*, 2018, **20**, 3509–3512.

5 N. M. Loim and E. S. Kelbyscheva, *Russ. Chem. Bull.*, 2004, **53**, 2080–2085.

6 T. Watana, Y. Tsunoda, A. Matsuo and A. Hirao, *Polym. Prepr. Japan*, 2006, **55**, 2583–2584.

7 M. Malkoch, K. Schleicher, E. Drockenmuller, C. J. Hawker, T. P. Russell, P. Wu and V. V. Fokin, *Macromolecules*, 2005, **38**, 3663–3678.

8 C. Deng, R. Fang, Y. Guan, J. Jiang, C. Lin and L. Wang, *Chem. Commun.*, 2012, **48**, 7973–7975.

9 F. Amir, Z. Jia and M. J. Monteiro, *J. Am. Chem. Soc.*, 2016, **138**, 16600–16603.

10 A. K. Agrahari, A. S. Singh., R. Mukherjee and V. K. Tiwari, *RSC Adv.*, 2020, **10**, 31553–31562.

11 S. Fujiki, K. Amaike, A. Yagi and K. Itami, *Nat. Commun.*, 2022, **13**, 5358.

12 T. Qin, X. Li, J. Chen, Y. Zeng, T. Yu, G. Yang and Y. Li, *Chem. – An Asian J.*, 2014, **9**, 3641–3649.

13 J. Yariv, A. J. Kalb and A. Levitki, *Biochim. Biophys. Acta*, 1968, **165**, 303–305.

14 T. Moulaei, S. R. Shenoy, B. Giomarelli, C. Thomas, J. B. McMahon, Z. Dauter, B. R. O'Keefe and A. Wlodawer, *Structure*, 2010, **18**, 1104–1115.
